# Nickel-catalyzed aminocarbonylation of aryl chlorides enabled by a newly designed CO source

**DOI:** 10.1039/d5sc07751f

**Published:** 2025-11-06

**Authors:** Zhen-Wei Liu, Yuanrui Wang, Ru-Han A., Xiao-Feng Wu

**Affiliations:** a Dalian National Laboratory for Clean Energy, Dalian Institute of Chemical Physics, Chinese Academy of Sciences Dalian China xwu2020@dicp.ac.cn; b Leibniz-Institut für Katalyse e.V. Rostock Germany Xiao-Feng.Wu@catalysis.de

## Abstract

Transition metal-catalyzed aminocarbonylation of aryl halides represents one of the most direct methods for synthesizing benzamides, a process which plays a pivotal role in a wide range of disciplines including chemistry, biology, medicine, and materials science. While recent advances have achieved more focus on aryl iodides and aryl bromides with palladium as the catalyst, the use of nickel as the catalyst in carbonylation of aryl chlorides remains challenging. Here, we report a nickel-catalyzed aminocarbonylation that overcomes this limitation, including more challenging aryl chlorides substituted with electron-donating groups. The success of this protocol is contingent on the use of inositol hexaformate as a newly developed CO surrogate. Control experiments with CO gas show that inositol hexaformate could release CO gradually in the reaction, avoiding nickel catalytic deactivation. This approach not only accomplishes a series of aminocarbonylation of aryl chlorides, but also provides a new arena for nickel-catalyzed carbonylation.

## Abstract 2

Enabled by a newly developed CO source, a nickel-catalyzed aminocarbonylation of aryl chlorides has been developed.

## Graphical abstract

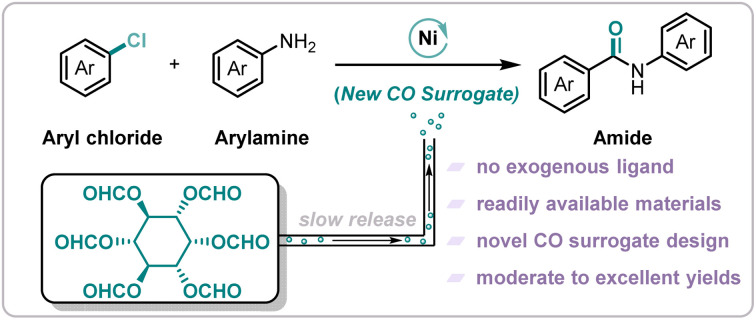

## Introduction

Nickel catalysts have gained considerable attention in catalytic chemistry due to their unique combination of economic and catalytic advantages. As an earth-abundant alternative to precious metals, nickel offers a distinct platform for sustainable synthesis.^1–5^ Positioned above palladium in Group 10 of the periodic table, nickel demonstrates both complementary and divergent catalytic behavior compared to its heavier congener. While capable of facilitating similar transformations to palladium, nickel's smaller atomic radius and accessible oxidation states from Ni(0) to Ni(iv) impart unique mechanistic pathways and selectivity patterns.^6–12^ The catalytic ability of nickel catalysts was proven by the achievements in inert bond activation and then coupling with various nucleophiles.^12^ Since the seminal discovery of Ni(CO)_4_ by Mond through the direct reaction of metallic nickel with carbon monoxide in 1890, nickel catalysts have experienced impressive progress.^13^ The early recognition of nickel's catalytic potential, coupled with its ongoing evolution as a versatile catalyst, underscores its enduring importance in modern synthetic chemistry.

**Fig. 1:**
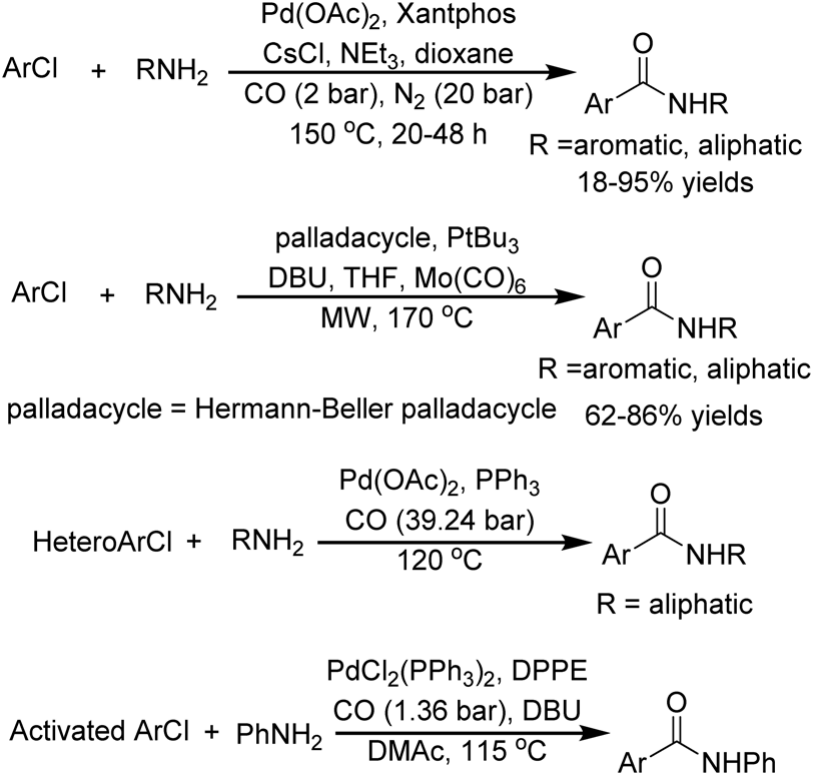
General conditions for previously developed palladium aminocarbonylation systems for ArCl.

**Scheme 1:**
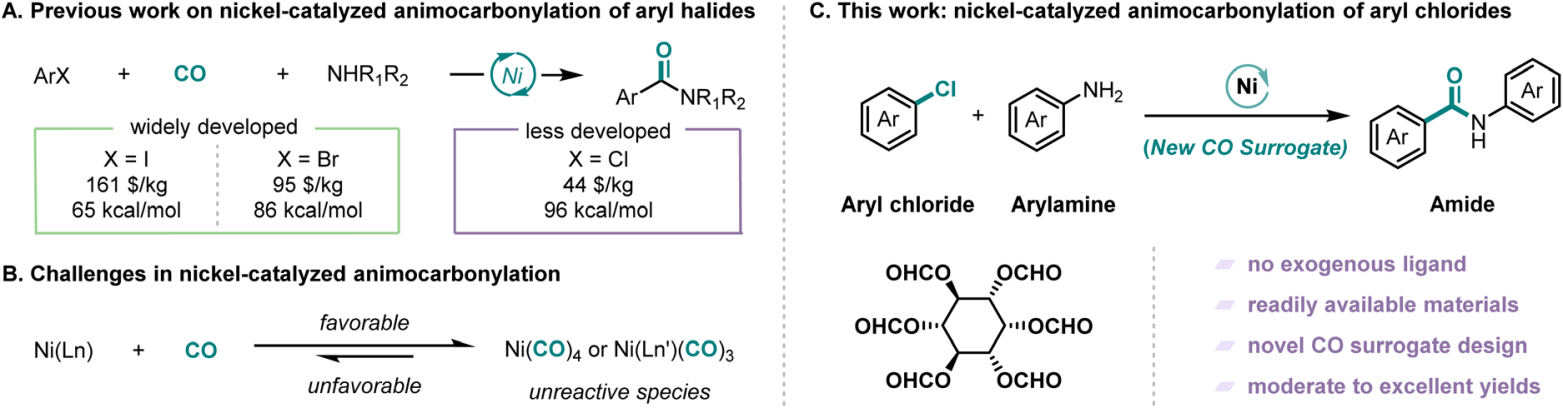
Development of nickel-catalyzed aminocarbonylation of aryl halides.

Benzamides constitute a privileged structural motif of paramount importance across multiple scientific disciplines, including medicinal chemistry and materials science. Their importance is also prompting the development of new and efficient synthetic methodologies.^14,15^ Among these, palladium-catalyzed aminocarbonylation of aryl halides has emerged as a particularly promising strategy, combining economic viability with synthetic efficiency. However, usually harsh reaction conditions (high temperature, high CO pressure, long reaction time, strong base, and high catalyst loading) were required in the case of using aryl chlorides as the substrates (Fig. 1).^16^ A nickel-catalyzed system was first reported by Giannoccaro and Pannacciulli in 1987.^17^ However, this system for iodobenzene and bromobenzene was carried out under relatively harsh reaction conditions (high temperature and high CO pressure) with monodentate triarylphosphine ligands. In 2023, Chen, Wu, and their co-workers were able to realize nickel-catalyzed aminocarbonylation of aryl iodide under atmospheric pressure of CO with xantphos or 1,10-phen as the ligand (Scheme 1A).^18^ However, the transformation of aryl chlorides remains substantially underdeveloped due to their characteristically higher C–Cl bond dissociation energies, approximately 96 kcal mol^−1^*versus* 86 kcal mol^−1^ for C–Br and 65 kcal mol^−1^ for C–I bonds, and consequently lower reactivity.^19^

The most significant challenge associated with nickel-catalyzed carbonylation reactions stems from the strong coordination ability of nickel with carbon monoxide, which readily leads to the formation of less reactive nickel carbonyl species such as Ni(CO)_4_ and Ni(CO)_3_L (Scheme 1B).^20–22^ Early studies on nickel-related carbonylation usually employed stoichiometric amounts of preformed Ni(CO)_4_, which also implies its low activity.^23,24^ To circumvent this intrinsic limitation, contemporary research has focused on two principal strategies: (i) the rational design of strongly coordinating, sterically demanding tridentate pincer ligands that effectively sequester the nickel center, thereby preventing deleterious over coordination while maintaining the metal in an active catalytic state^25^ and (ii) the development of stable and controllable CO surrogates, which can slowly release carbon monoxide in the system and establish a dynamic equilibrium between carbon monoxide generation and consumption, thus avoiding the generation of low reactive nickel complexes and stabilizing the catalytic cycle.^26^

In recent years, continuous research conducted by chemists has resulted in the development of numerous CO surrogates that demonstrate excellent performance. These include formic acid derivatives,^27–29^ oxalyl chloride,^30^ chloroformic acid derivatives,^31–33^ isonitrile derivatives,^34,35^ carbonyl metals,^36–39^ COgen,^40^ and SilaCOgen.^41^ The development of nickel-catalyzed carbonylation has been greatly facilitated by the introduction of these CO surrogates. However, a significant challenge in nickel-catalyzed carbonylation of inert bonds arises from the inherent requirement for elevated reaction temperatures. This thermal demand creates a fundamental incompatibility with conventional CO surrogates, which typically exhibit rapid decarbonylation under such conditions. The consequent uncontrolled CO release leads to rapid, localized concentration spikes that poison the nickel catalyst through excessive carbonyl complex formation. This critical limitation has driven increasingly stringent performance criteria for novel CO surrogates in modern nickel catalysis. Herein we report a nickel-catalyzed aminocarbonylation of aryl chlorides employing inositol hexaformate as a novel CO surrogate (Scheme 1C). Inositol, an inexpensive and naturally occurring vitamin B derivative abundant in legumes, nuts and grains, possesses a unique cyclohexanehexol structure that renders it particularly suitable for CO surrogate development.^42^ While aryl chlorides have traditionally been challenging substrates for carbonylation due to their low reactivity, their high commercial availability and cost-effectiveness make them attractive targets for catalytic transformations. The coordination of CO with metal decreases its electron density, which is crucial for inert bond activation, hence the reaction temperature was increased. However, high temperature favors decarbonylation and leads to increasing CO pressure to ensure CO insertion. This contradictory situation makes the reaction conditions harsh in a spiral manner and finally leads to high temperature and high CO pressure. Our methodology successfully addresses these challenges through the strategic implementation of the bio-derived inositol-based CO surrogate, which enables the efficient nickel-catalyzed aminocarbonylation of aryl chlorides.

## Results and discussion

We initiated nickel-catalyzed aminocarbonylation studies of aryl chlorides using chlorobenzene and aniline as the template substrates, nickel acetylacetonate as the pre-catalyst, phenylsilane as the reducing agent, inositol hexaformate (HFI) as the carbon monoxide source, 1,8-diazabicyclo[5.4.0]undec-7-ene (DBU) as the base and *N*-methyl-2-pyrrolidone (NMP) as the solvent, and the reaction was carried out at 140 °C for 24 h (Table 1). Benzamide 1 was obtained in 82% GC yield and 81% isolated yield even at an enlarged scale (Table 1, entry 1). The yield of benzamide decreased when bis(cyclooctadiene)nickel(0) was tested as the catalyst (Table 1, entry 2). Zinc metal, a common reducing agent in nickel-catalyzed carbonylation, was not suitable in this reaction system (Table 1, entry 3). Surprisingly, 66% yield of the target product was obtained when bis(pinacolato)diboron was used as the reducing agent, a result that might indicate that the organic reducing agent favors the reaction (Table 1, entry 4). Unfortunately, when an organic weak base or an inorganic base was used, the reaction essentially did not occur (Table 1, entries 5 and 6). When the solvent was replaced with the commonly used acetonitrile or the low-polar tetrahydrofuran, the yield of the desired amide decreased considerably, suggesting that solvent effects have a significant impact on the reaction (Table 1, entries 7 and 8). The amount of carbon monoxide source (HFI) also affected the reaction results, either by decreasing the standard amount or increasing it, which indicates that the concentration of carbon monoxide in the reaction system is critical (Table 1, entries 9 and 10), and precise control of the carbon monoxide concentration is not available with conventional CO gas. When using molybdenum hexacarbonyl with the same carbon monoxide loading, similar results to HFI could not be obtained (Table 1, entry 11). When one atmosphere of CO gas was used, the reaction essentially did not proceed (Table 1, entry 12), which again suggests that precise modulation of the carbon monoxide content is the key to obtaining high yield for this reaction. Additionally, HCOOH + Ac_2_O and phenyl formate were also checked as a CO source. However, no desired product was detected due to their fast release of CO and the interaction with aniline to give formanilide. When phenylsilane was removed, the reaction did not proceed properly (Table 1, entry 13), indicating that the addition of a catalytic amount of phenylsilane promotes the reaction. When nickel acetylacetonate or HFI was removed, the reaction did not occur (Table 1, entries 14 and 15), suggesting that nickel and HFI are essential for the reaction.

**Table 1:** Reaction optimization^a^

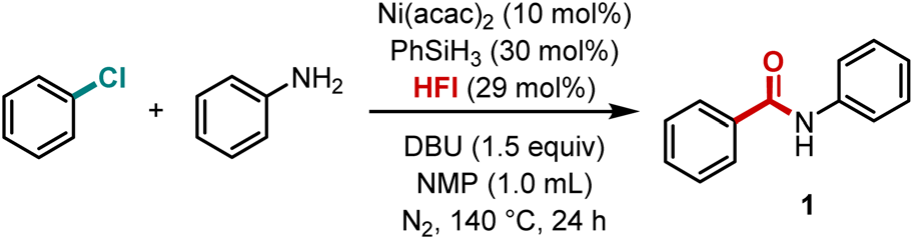		
Entry	Variation from standard conditions	1^b^ (%)
1	None	82 (81)^c^
2	Ni(cod)_2_	66
3	Zn	37
4	B_2_pin_2_	66
5	TEA	4
6	Na_2_CO_3_	7
7	MeCN	23
8	THF	8
9	HFI (25 mol%)	65
10	HFI (33 mol%)	43
11	Mo(CO)_6_	40
12	CO gas	8
13	w/o PhSiH_3_	10
14	w/o Ni(acac)_2_	0
15	w/o HFI	0

a Reactions were performed on a 0.2 mmol scale.

b Yields were determined by GC-MS analysis of the crude product using *n*-dodecane as the internal standard.

c Isolated yield on a 1 mmol scale.

**Scheme 2:**
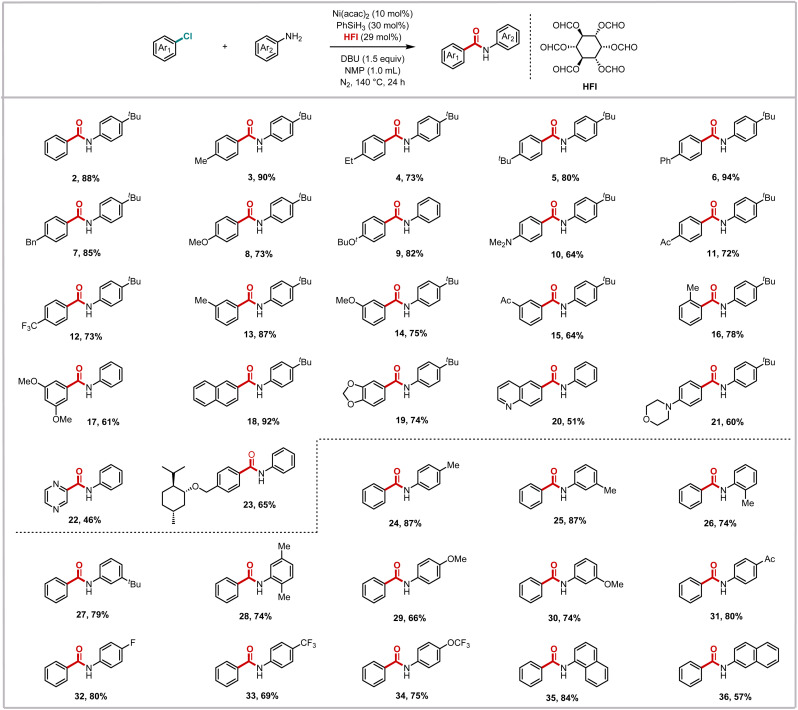
Nickel-catalyzed aminocarbonylation of aryl chlorides.

Building upon the optimized reaction conditions, we subsequently explored the substrate scope of this nickel-catalyzed aminocarbonylation system with respect to aryl chlorides (Scheme 2). The methodology demonstrated remarkable generality, accommodating a diverse range of substituted aryl chlorides that reacted smoothly with aryl amines to afford the corresponding benzamide derivatives in moderate to excellent yields. It is noteworthy that the system demonstrated remarkable compatibility with both electron-deficient substrates and challenging electron-rich substrates. *para*-Substituted chlorobenzenes bearing electron-donating groups, such as methyl (3), ethyl (4), *tert*-butyl (5), phenyl (6), and benzyl (7), or strongly donating groups, including alkoxy (8 and 9) and *N*,*N*-dimethyl (10), exhibited efficient conversion. This efficiency was also observed in substrates with electron-withdrawing substituents, such as acetyl (11) and trifluoromethyl (12). This consistent performance across substrates with varying electronic properties suggests that the reaction is relatively insensitive to the electronic effects of substituents.

The robustness of the system was further demonstrated by its successful application to more sterically demanding substrates. While *meta*-substituted chlorobenzenes reacted efficiently regardless of their electronic properties (13–16), *ortho*-substituted variants exhibited slightly diminished yields, likely due to steric hindrance around the reactive center. Notably, even highly substituted systems such as dimethoxychlorobenzene participated effectively (17), albeit with somewhat reduced efficiency. The efficacy of the methodology was also demonstrated in the context of extended π-systems, with chloronaphthalene being a pertinent example (18), in addition to heteroaromatic chlorides (19–22). Most remarkably, the system's mild conditions and functional group tolerance enabled the successful modification of complex natural product derivatives (23), highlighting its potential for late-stage functionalization in bioactive molecule synthesis.

We then evaluated the scope of aryl amines in this nickel-catalyzed aminocarbonylation system. It was determined that the corresponding amide products could be obtained in excellent yields, irrespective of the methyl group in the *para*-, *meta*- and *ortho*-positions of the aniline (24–26). The yields for *tert*-butyl-substituted aniline (27) and polymethyl-substituted aniline (28) were not negligible, suggesting that the steric effect has a negligible influence on this reaction system. When the anilines with a methoxy substituent in the *para*- and *meta*-positions were subjected to the reaction, the target products were obtained in 66% and 74% yields, respectively (29 and 30). It is evident that the reaction of aniline with an acetyl group is more favorable, which suggests that the electron-withdrawing group was conducive to the reaction (31). Following the incorporation of a fluorine atom into the molecule, an enhancement was observed in the physical and chemical properties of the molecule. Anilines containing varying fluorine-containing groups were thus examined, and it was ascertained that all of them reacted in a satisfactory manner (32–34). Moreover, it has been demonstrated that a series of naphthylamines were compatible with this reaction mode (35 and 36). However, no desired product was detected when aliphatic amines, heteroaryl amines, alcohols, or carbon nucleophiles were tested under our standard conditions.

**Scheme 3:**
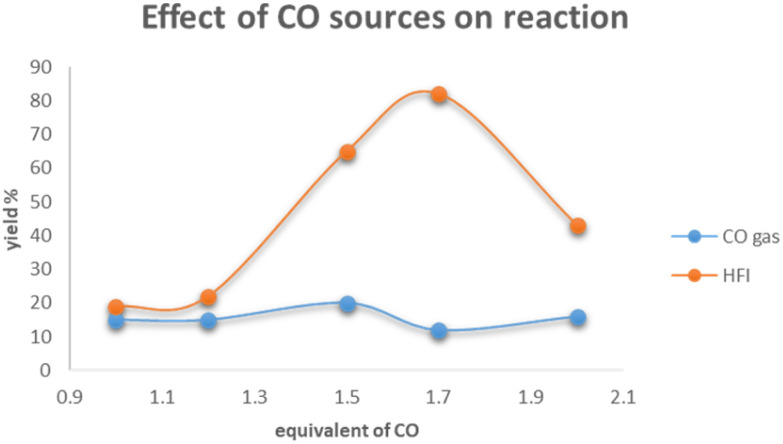
Effect of CO sources on the aminocarbonylation reaction.

In order to ascertain the key factors for the success of nickel-catalyzed aminocarbonylation of aryl chlorides, a set of controlled experiments was performed (Scheme 3). As demonstrated in the figure, the utilization of CO gas in conjunction with varying equivalence gradients generates a yield that oscillates within the range of 10% to 20%, exhibiting minimal variance. Conversely, the employment of HFI in conjunction with different equivalence gradients results in an initial increase in yield, followed by a precipitous decline once the equivalence reaches 2.0. The results of the control experiment led to the formulation of a hypothesis concerning the rate of carbon monoxide (CO) release by HFI during the reaction. The hypothesis posited that HFI did not release CO rapidly; rather, it was hypothesized that HFI released CO continuously and steadily. Furthermore, it was postulated that the rate of CO release by HFI was comparable^43^ to the rate at which the reaction consumed CO. It was thus concluded that these conditions were conducive to the successful progression of the reaction.^44^

**Scheme 4:**
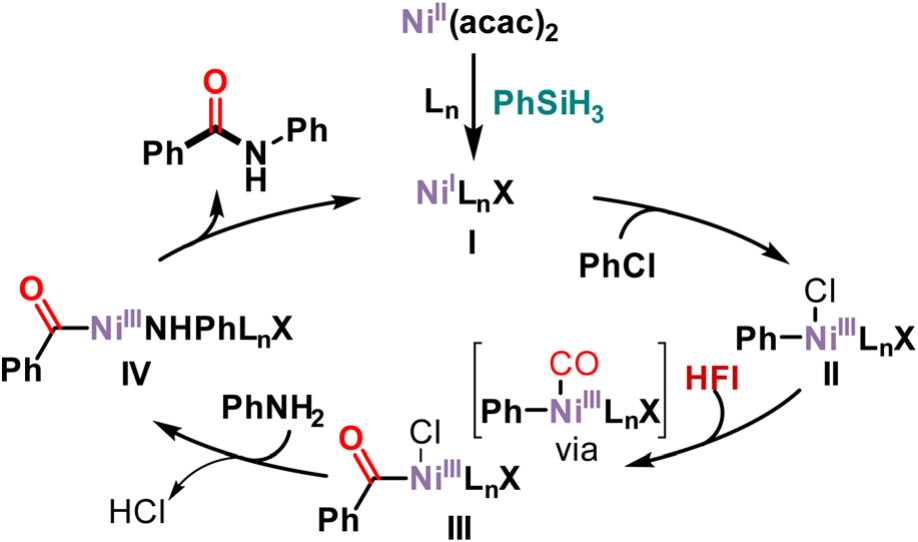
Proposed mechanism.

Based on our results and the literature,^45–47^ a possible mechanism is presented in Scheme 4. First, nickel acetylacetonate was reduced in the presence of phenylsilane to Ni(i) complex I. Subsequently, chlorobenzene undergoes oxidative addition with Ni(i) complex I to form Ni(iii) complex II. HFI releases CO at high temperature or in the presence of DBU. The released CO then coordinated with nickel and then migrated and inserted into Ni(iii) complex II to form acyl-Ni(iii) complex III. Anionic exchange with aniline occurs to form Ni(iii) complex IV. Then an anion exchange with aniline produces Ni(iii) complex IV. Finally, reductive elimination of Ni(iii) complex IV yields the amide along with the active species Ni(i) complex I to complete the catalytic cycle.

## Conclusions

In summary, we have developed a nickel-catalyzed aminocarbonylation of aryl chlorides, and the key to the success lies in the development of a novel and efficient inositol hexaformate as the CO surrogate. Control experiments with CO gas indicate that inositol hexafluoride acts as a slow-release CO surrogate, preventing nickel catalyst deactivation during the reaction. The reaction applies to a range of aryl chlorides, including the challenging aryl chlorides with electron-donating groups attached. Our approach extends the range of substrates for benzamide synthesis and offers the possibility of reducing the cost of benzamides.

## Author contributions

Z. W. L. performed all the experiments and prepared the manuscript and SI. Y. W. and R. H. A prepared some substrates. X. F. W. conceived the project, supervised the research, and revised the manuscript.

## Conflicts of interest

There are no conflicts to declare.

## Supplementary Material

SC-016-D5SC07751F-s001

SC-016-D5SC07751F-s002

## Data Availability

CCDC 2393156 contains the supplementary crystallographic data for this paper.^48^ The data supporting this article have been included as part of the supplementary information (SI). Supplementary information: general comments, general procedure, analytical data, and NMR spectra. See DOI: https://doi.org/10.1039/d5sc07751f.
