# Supplementary material for: Nickel-catalyzed aminocarbonylation of aryl chlorides enabled by a newly designed CO source
Source: Chem Sci. 2025 Nov 6;16(48):23315–20. doi: 10.1039/d5sc07751f (PMC12591056; doi:10.1039/d5sc07751f)
Supplement: SC-016-D5SC07751F-s001 [file SC-016-D5SC07751F-s001.pdf]

Supplementary Information (SI) for Chemical Science.
This journal is © The Royal Society of Chemistry 2025

Supporting Information

Table of Contents

General Information **1**
Safety Considerations **2**
Synthesis of Inositol hexaformate (HFI) **3**
Optimization of conditions **4**
Table S1. The results of the nickel catalyst 4
Table S2. The results of the base 5
Table S3. The results of the ligand 6
Table S4. The results of the HFI 7
Table S5. The results of the solvent 8
Table S6. The results of the $Ni(acac)_2$ 9
Table S7. The results of the $PhSiH_3$ 9
Table S8. The results of the room temperature and reaction temperature 10
Table S9. The results of the PhCl and $PhNH_2$ 10
Table S10. The results of the DBU 11
Experimental procedures and data **12**
General Procedure for Nickel-Catalyzed Aminocarbonylation of Aryl chlorides 12
Analytical data for Products 13
X-ray Crystallographic Data **30**
Table S10. Crystal data and structure refinement for Inositol hexaformate. 30
NMR Data **32**

General Information

Unless otherwise noted, all reactions were performed under positive pressure of nitrogen atmosphere in oven-dried flasks. All commercially available reagents were purchased from commercial vendors Sigma-Aldrich, Adamas, or Energy Chemical and used without further purification. Reactions were monitored by thin layer chromatography (TLC) (HaiYang, QingDao, China), visualized by UV (254 nm) and phosphomolybdic acid (PMA) staining. Flash column chromatography was performed on silica gel (200 ~ 300 mesh) purchased from Haiyang (Qingdao, China) and using petroleum ether (b.p. 60-90 ^{o}C) and ethyl acetate as the eluents.

Gas chromatography (**GC**) analyses were performed on an Agilent HP-7890A instrument with an FID detector and HP-5 capillary column (polydimethylsiloxane with 5% phenyl groups, 30 m, 0.32 mm i.d. 0.25 μm film thickness) using argon as carrier gas. Gas chromatography mass spectrometer (**GC-MS**) analyses were performed on a Shimadzu QP2020 NX instrument. **NMR** spectra were recorded on Bruker AVANCE III 400 MHz and Bruker AVANCE III 700 MHz. Chemical shifts (δ) were reported in ppm relative to residual solvent peak or tetramethylsilane as internal standard ($(CD_3)_2SO$: 2.50 ppm for ^{1}H NMR, 39.52 ppm for ^{13}C NMR). Multiplicity and qualifier abbreviations are as follows: s = singlet, d = doublet, t = triplet, q = quartets, dd = doublet of doublets, ddd = doublet of doublet of doublets, dddd = doublet of doublet of doublet of doublets, dt = doublet of triplets, dq = doublet of quartets, ddq = doublet of doublet of quartets, td = triplet of doublets, qd = quartet of doublets, m = multiplet. Highresolution mass spectral analysis (**HRMS**) data were determined on an Agilent 8890-7250 and Agilent Q-TOF 6540 spectrometer by means of ESI technique.

Safety Considerations

Carbon monoxide:

Carbon monoxide is highly toxic. The material should be handled by trained and experienced researchers, utilizing physical control (fume hoods) and safety measures (gas sensors). Experiments involving CO were conducted with a personal monitor worn on the researcher's lab coat at all times and an additional monitor placed near the regulator of the CO cylinder.

Nickel tetracarbonyl:

Extremely toxic and volatile (b.p. 43 ºC) $Ni(CO)_4$ could be potentially generated during nickel-catalyzed carbonylation reactions. Reactors which may contain $Ni(CO)_4$ should be handled by trained and experienced researchers, utilizing physical controls (fume hoods, chemically-resistant gloves) and safety measures (gas sensors). Any gas and solution that could potentially contain $Ni(CO)_4$ was quenched with a solution of iodine in acetone. After the desired reaction time, the reactors were allowed to cool to approximately 0 °C in an ice bath, after which 1.0 mL solutions of 20 wt% iodine in acetone were added (to quench any $Ni(CO)_4$). The mixture should then be stirred until it has warmed to room temperature. The generation of carbon monoxide (CO) has been observed to occur during this specific period.

Synthesis of Inositol hexaformate (HFI)

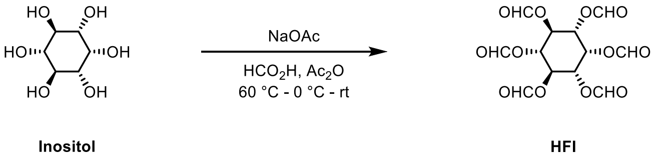

To a 250 mL flask with a magnetic stir bar and a multi-layer balloon was added 23 mL formic acid and 45 mL acetic anhydride, the mixture was stirred at 60 °C for 1 h under Air. Then the reaction was cooled to approximately 0 °C in an ice bath, after which 1.8 g inositol and 9.8 g sodium acetate were added **(Notice: A lot of carbon monoxide is produced at this point)**. After stirring the mixture for 24 hours, 100 mL of water was added to quench it, and then the reaction system was extracted with dichloromethane until the aqueous phase became clear. The collected organic phase was dried with anhydrous sodium sulfate, and finally concentrated by rotary evaporation and vacuum drying to obtain 3.4g of white solid.

(1R,2r,3S,4R,5s,6S)-cyclohexane-1,2,3,4,5,6-hexayl hexaformate

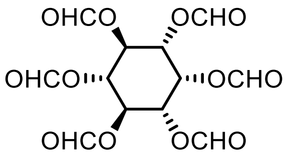

3.4 g, white solid, yield: 98%.

^{1}H NMR (400 MHz, DMSO-d_6) δ 8.54 – 8.48 (m, 1H), 8.32 (dd, *J* = 14.2, 0.7 Hz, 3H), 8.23 (d, *J* = 0.7 Hz, 2H), 5.78 – 5.61 (m, 4H), 5.47 (t, *J* = 9.8 Hz, 2H).

^{13}C NMR (101 MHz, DMSO-d_6) δ 161.3, 161.2, 160.8, 160.7, 68.5, 68.3, 67.6, 67.1.

Optimization of conditions

Table S1. The results of the nickel catalyst

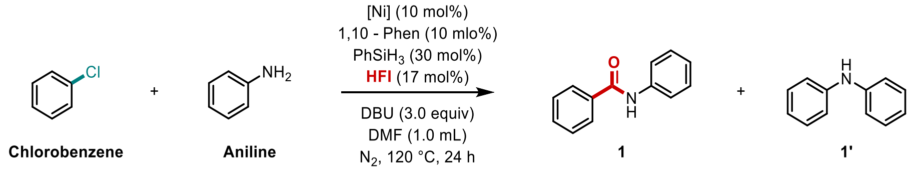

entry	catalyst	1 (%)	1′(%)	entry	catalyst	1 (%)	1′(%)
1	$NiCl_2$	42	15	11	$Ni(CF_3SO_3)_2$	35	18
2	$NiBr_2$	42	12	12	$Ni(acac)_2$	43	5
3	NiI_2	39	14	13	$Ni(HFacac)_2$	39	11
4	$NiCl_2·6H_2O$	40	12	14	$NiCl_2$·DME	39	8
5	$NiBr_2·3H_2O$	42	14	15	$NiBr_2$·DME	40	11
6	$NiI_2·6H_2O$	43	12	16	$NiCl_2·2PCy_3$	14	4
7	$Ni(NO_3)_2·6H_2O$	43	11	17	$NiCl_2·2PPh_3$	35	9
8	$Ni(CLO_4)_2·6H_2O$	39	13	18	$NiCl_2$·dppe	36	5
9	$Ni(cod)_2$	40	7	19	$NiCl_2$·dppp	19	5
10	$Ni(TMHD)_2$	40	11	20	$NiCl_2$·dppf	9	3

[a]Reaction conditions: chlorobenzene (0.2 mmol, 1.0 equiv), aniline (4.0 equiv), **catalyst** (10 mol%), 1,10-Phen (10 mol%), $PhSiH_3$ (30 mol%), HFI (17 mol%), DBU (3.0 equiv), DMF (1.0 mL), 120 °C, 24 h. Yield was determined by GC-MS analysis of the crude product using *n*dodecane as the internal standard.

Table S2. The results of the base

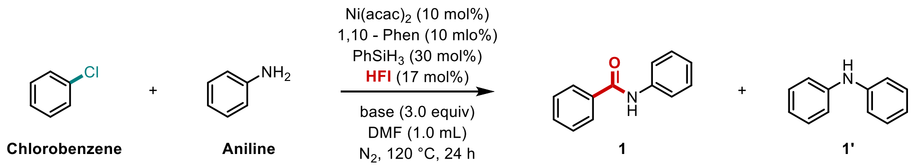

entry	base	1 (%)	1´(%)
1	DBU	43	5
2	DBN	40	6
3	DABCO	6	0
4	TBD	0	0
5	MTBD	12	3
6	TMG	11	4
7	^{t}Bu-TMG	8	0
8	TEA	9	0
9	DIPEA	6	0
10	Cy_2NMe	0	0
11	DMAP	8	0
12	Quinclidine	13	5
13	K_2CO_3	0	0
14	Na_2CO_3	5	0

^{a}Reaction conditions: chlorobenzene (0.2 mmol, 1.0 equiv), aniline (4.0 equiv), $Ni(acac)_2$ (10 mol%), 1,10-Phen (10 mol%), $PhSiH_3$ (30 mol%), HFI (17 mol%), **base** (3.0 equiv), DMF (1.0 mL), 120 °C, 24 h. Yield was determined by GC-MS analysis of the crude product using ^{n}dodecane as the internal standard.

Table S3. The results of the ligand

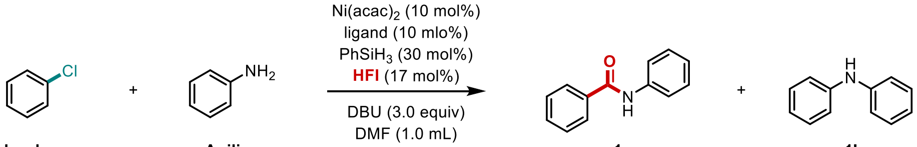

Chlorobenzene + **Aniline** → **1** + **1'**

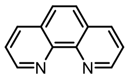	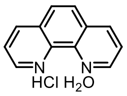	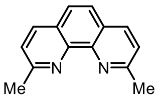	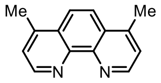	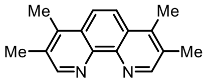
**1, 38%; 1', 6%**	**1, 40%; 1', 5%**	**1, 9%; 1', 3%**	**1, 40%; 1', 10%**	**1, 36%; 1', 14%**
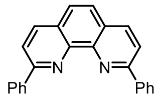	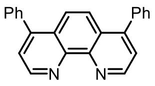	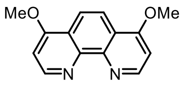	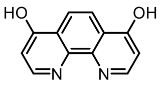	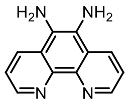
**1, 34%; 1', 28%**	**1, 36%; 1', 8%**	**1, 35%; 1', 11%**	**1, 29%; 1', 8%**	**1, 29%; 1', 5%**
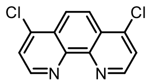	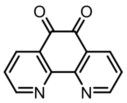	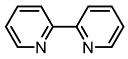	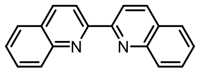	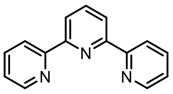
**1, 36%; 1', 9%**	**1, 34%; 1', 5%**	**1, 30%; 1', 9%**	**1, 39%; 1', 5%**	**1, 26%; 1', 3%**
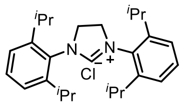	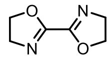	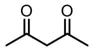	**No ligand**	
**1, 35%; 1', 14%**	**1, 29%; 1', 7%**	**1, 17%; 1', 6%**	**1, 42%; 1', 19%**	

[a]Reaction conditions: chlorobenzene (0.2 mmol, 1.0 equiv), aniline (4.0 equiv), $Ni(acac)_2$ (10 mol%), ligand (10 mol%), $PhSiH_3$ (30 mol%), HFI (17 mol%), DBU (3.0 equiv), DMF (1.0 mL), 120 °C, 24 h. Yield was determined by GC-MS analysis of the crude product using ndodecane as the internal standard.

Table S4. The results of the HFI

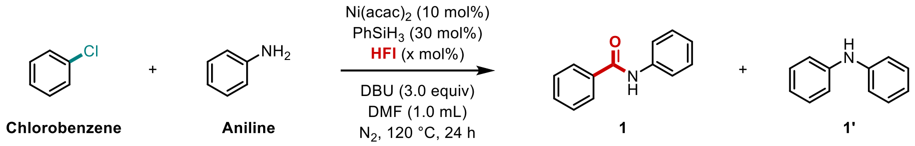

entry	x	1 (%)	1′(%)
1	17	35	14
2	20	43	14
3	25	58	8
4	29	68	8
5	33	5	3

[a]Reaction conditions: chlorobenzene (0.2 mmol, 1.0 equiv), aniline (4.0 equiv), Ni(acac)$_2$ (10 mol%), $PhSiH_3$ (30 mol%), HFI (x mol%), DBU (3.0 equiv), DMF (1.0 mL), 120 °C, 24 h. Yield was determined by GC-MS analysis of the crude product using ^{n}dodecane as the internal standard.

Table S5. The results of the solvent

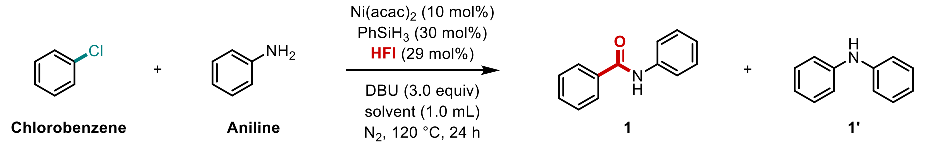

entry	solvent	1 (%)	1′(%)
1	DMSO	12	3
2	DMAc	65	7
3	DMF	61	9
4	NMP	65	5
5	MeCN	33	17
6	THF	27	14
7	Dioxane	4	4
8	Hexane	7	15
9	DCE	0	0
10	Toluene	25	12
11	$PhCF_3$	26	13
12	PhOMe	37	12
13	HFIP	0	0
14	EA	18	9
15	Acetone	7	5
16	DME	42	17

[a]Reaction conditions: chlorobenzene (0.2 mmol, 1.0 equiv), aniline (4.0 equiv), $Ni(acac)_2$ (10 mol%), $PhSiH_3$ (30 mol%), HFI (29 mol%), DBU (3.0 equiv), solvent (1.0 mL), 120 °C, 24 h. Yield was determined by GC-MS analysis of the crude product using [n]dodecane as the internal standard.

Table S6. The results of the Ni(acac)2

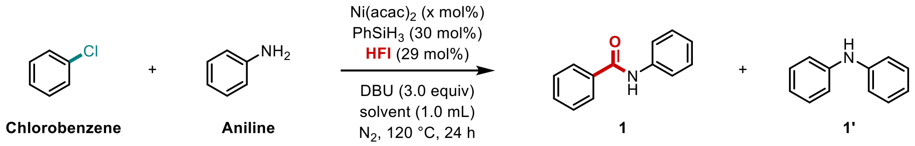

entry	x	1 (%)	1′(%)
1	2.5	8	0
2	5.0	16	0
3	7.5	30	0
4	10.0	75	6
5	12.5	68	8
6	15.0	40	8
7	17.5	23	7
8	20.0	18	6

[a]Reaction conditions: chlorobenzene (0.2 mmol, 1.0 equiv), aniline (4.0 equiv), $Ni(acac)_2$ (x mol%), $PhSiH_3$ (30 mol%), HFI (29 mol%), DBU (3.0 equiv), NMP (1.0 mL), 120 °C, 24 h. Yield was determined by GC-MS analysis of the crude product using *n*dodecane as the internal standard.

Table S7. The results of the PhSiH3

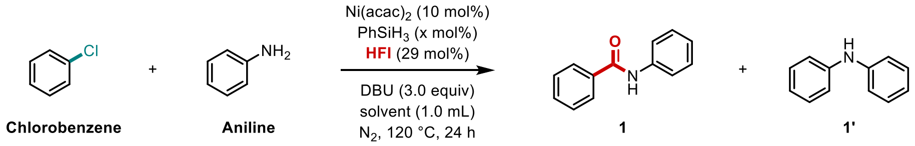

entry	x	1 (%)	1′(%)
1	10	13	5
2	20	20	4
3	30	68	6
4	40	65	12
5	50	72	7

[a]Reaction conditions: chlorobenzene (0.2 mmol, 1.0 equiv), aniline (4.0 equiv), $Ni(acac)_2$ (10 mol%), $PhSiH_3$ (x mol%), HFI (29 mol%), DBU (3.0 equiv), NMP (1.0 mL), 120 °C, 24 h. Yield was determined by GC-MS analysis of the crude product using *n*dodecane as the internal standard.

Table S8. The results of the room temperature and reaction temperature

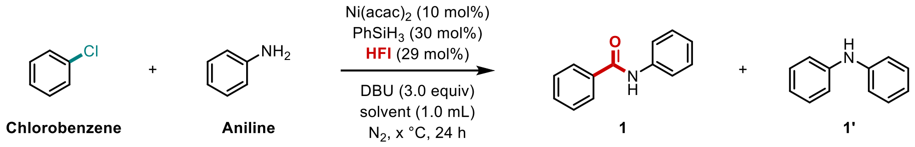

entry	room temperature	reaction temperature	1 (%)	1′(%)
1	< 10 °C	120 °C	68	10
2	> 10 °C	120 °C	16	4
4	> 10 °C	140 °C	73	10

[a]Reaction conditions: chlorobenzene (0.2 mmol, 1.0 equiv), aniline (4.0 equiv), $Ni(acac)_2$ (10 mol%), $PhSiH_3$ (30 mol%), HFI (29 mol%), DBU (3.0 equiv), NMP (1.0 mL), x °C, 24 h. Yield was determined by GC-MS analysis of the crude product using ^{n}dodecane as the internal standard. [b]At a reaction temperature of 130 °C, the yield was unstable regardless of room temperature. [c]In Dalian, Liaoning Province, China, the room temperature was above 10 °C since March.

Table S9. The results of the PhCl and $PhNH_2$

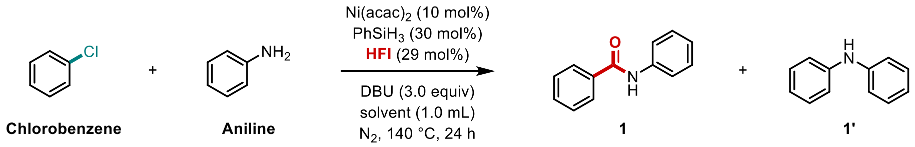

entry	x : y	1 (%)	1′(%)
1	1 : 4	72	8
2	1 : 3	72	10
3	1 : 2	65	9
4	1 : 1.5	61	9
5	1 : 1	49	9
6	1.5 : 1	51	13
7	2 : 1	51	14

[a]Reaction conditions: chlorobenzene (x equiv), aniline (y equiv), $Ni(acac)_2$ (10 mol%), $PhSiH_3$ (30 mol%), HFI (29 mol%), DBU (3.0 equiv), NMP (1.0 mL), 140 °C, 24 h. Yield was determined by GC-MS analysis of the crude product using ^{n}dodecane as the internal standard.

Table S10. The results of the DBU

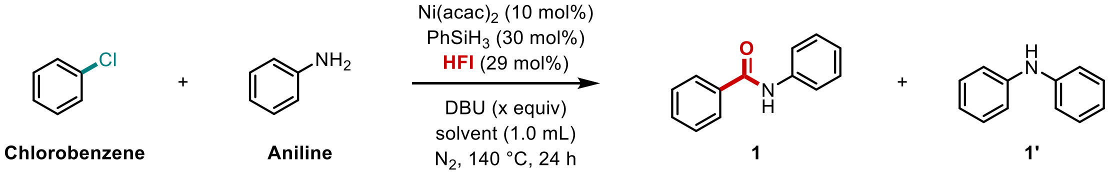

entry	x	1 (%)	1´(%)
1	0.5	35	6
2	1.0	53	11
3	1.5	75	11
4	2.0	75	10
5	2.5	75	9
6	3.0	75	10
7	3.5	75	8

[a]Reaction conditions: chlorobenzene (0.2 mmol, 1.0 equiv), aniline (3.0 equiv), $Ni(acac)_2$ (10 mol%), $PhSiH_3$ (30 mol%), HFI (29 mol%), DBU (x equiv), NMP (1.0 mL), 140 °C, 24 h. Yield was determined by GC-MS analysis of the crude product using *n*dodecane as the internal standard.

Experimental procedures and data

General Procedure for Nickel-Catalyzed Aminocarbonylation of Aryl chlorides

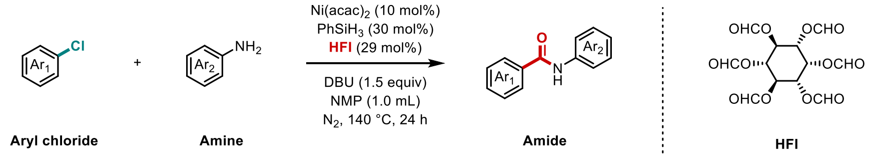

Ni(acac)$_2$ (5.1 mg, 10 mol%) and HFI (20.2 mg, 29 mol%) were added to an oven-dried tube (15 mL) under air. After flushed the tube with nitrogen three times, it was shifted into glovebox. Then NMP (1.0 mL), aryl chloride (0.2 mmol, 1.0 equiv), aniline (3.0 equiv), DBU (45 μL, 1.5 equiv) and PhSiH$_3$ (7μL, 30 mol%) were added to the tube in glovebox. The tube was sealed and taken out of the glove box. The mixture was stirred at 140 °C for 24 h. After the reaction was completed, the reactors were allowed to cool to approximately 0 °C in an ice bath, after which 1.0 mL solutions of 20 wt% iodine in acetone were added (to quench any Ni(CO)$_4$). The mixture should then be stirred until it warmed to room temperature. Ethyl acetate (5.0 mL) was added to it, and the reaction system was washed with 2.0 N aqueous hydrochloric acid (5.0 mL, 3 times) and then the aqueous phase was extracted with ethyl acetate (3 times). The collected organic phase was dried with anhydrous sodium sulfate, and concentrated by rotary evaporation. The crude product was purified by column chromatography on silica gel to afford the corresponding product amide.

Analytical data for Products

***N*-phenylbenzamide**

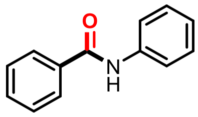

31.9 mg, white solid, yield: 81%.

Rf = 0.3 (silica gel, petroleum ether : ethyl acetate = 6:1)

^{1}H NMR (400 MHz, DMSO-*d_6*) δ 10.25 (s, 1H), 8.04 – 7.92 (m, 2H), 7.86 – 7.73 (m, 2H), 7.65 – 7.48 (m, 3H), 7.36 (dd, *J* = 8.6, 7.4 Hz, 2H), 7.10 (ddd, *J* = 7.3, 6.7, 1.3 Hz, 1H).

^{13}C NMR (101 MHz, DMSO-*d_6*) δ 165.6, 139.2, 135.0, 131.5, 128.6, 128.4, 127.7, 123.7, 120.4.

HRMS (ESI-TOF) m/z: $[M+H]^+$ calcd for $C_{13}H_{12}NO^+$ 198.0913; found: 198.0914.

***N*-(4-(*tert*-butyl)phenyl)benzamide**

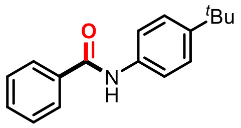

44.7 mg, white solid, yield: 88%.

Rf = 0.3 (silica gel, petroleum ether : ethyl acetate = 8:1)

^{1}H NMR (400 MHz, DMSO-*d_6*) δ 10.19 (s, 1H), 8.04 – 7.91 (m, 2H), 7.71 (d, *J* = 8.8 Hz, 2H), 7.63 – 7.44 (m, 3H), 7.36 (d, *J* = 8.7 Hz, 2H), 1.28 (s, 9H).

^{13}C NMR (101 MHz, DMSO-*d_6*) δ 165.3, 146.0, 136.6, 135.0, 131.4, 128.3, 127.6, 125.2, 120.1, 34.0, 31.2.

HRMS (ESI-TOF) m/z: $[M+H]^+$ calcd for $C_{17}H_{20}NO^+$ 254.1539; found: 254.1546.

***N*-(4-(*tert*-butyl)phenyl)-4-methylbenzamide**

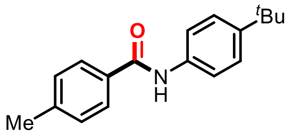

48.0 mg, white solid, yield: 90%.

Rf = 0.3 (silica gel, petroleum ether : ethyl acetate = 10:1)

^{1}H NMR (400 MHz, DMSO-*d₆*) δ 10.09 (s, 1H), 7.88 (d, *J* = 8.3 Hz, 2H), 7.70 (d, *J* = 8.8 Hz, 2H), 7.34 (dd, *J* = 11.7, 8.6 Hz, 4H), 2.38 (s, 3H), 1.28 (s, 9H).

^{13}C NMR (101 MHz, DMSO-d_6) δ 197.7, 164.5, 146.3, 138.8, 138.8, 136.3, 128.2, 128.0, 125.3, 120.2, 34.1, 31.2, 27.0.

HRMS (ESI-TOF) m/z: $[M+H]^+$ calcd for $C_{18}H_{22}NO^+$ 268.1696; found: 268.1694.

***N*-(4-(*tert*-butyl)phenyl)-4-ethylbenzamide**

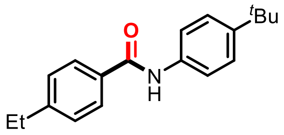

38.1 mg, white solid, yield: 68%.

Rf = 0.3 (silica gel, petroleum ether : ethyl acetate = 12:1)

^{1}H NMR (400 MHz, DMSO-d_6) δ 10.09 (s, 1H), 7.89 (d, *J* = 8.4 Hz, 2H), 7.70 (d, *J* = 8.8 Hz, 2H), 7.35 (dd, *J* = 8.4, 1.6 Hz, 4H), 2.68 (q, *J* = 7.6 Hz, 2H), 1.28 (s, 9H), 1.21 (t, *J* = 7.6 Hz, 3H).

^{13}C NMR (101 MHz, DMSO-d_6) δ 165.2, 147.6, 145.8, 136.7, 132.5, 127.7, 127.7, 125.2, 120.1, 34.0, 31.2, 28.1, 15.4.

HRMS (ESI-TOF) m/z: $[M+H]^+$ calcd for $C_{19}H_{24}NO^+$ 282.1852; found: 282.1857.

4-(*tert*-butyl)-*N*-(4-(*tert*-butyl)phenyl)benzamide

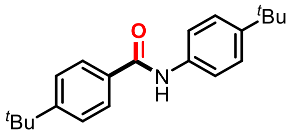

49.7 mg, colorless oil, yield: 80%.

Rf = 0.3 (silica gel, petroleum ether : ethyl acetate = 15:1)

^{1}H NMR (400 MHz, DMSO-*d$_6$*) δ 10.10 (s, 1H), 7.89 (d, *J* = 8.4 Hz, 2H), 7.69 (d, *J* = 8.7 Hz, 2H), 7.53 (d, *J* = 8.4 Hz, 2H), 7.35 (d, *J* = 8.8 Hz, 2H), 1.30 (d, *J* = 15.9 Hz, 18H).

^{13}C NMR (101 MHz, DMSO-*d$_6$*) δ 165.3, 154.3, 145.8, 136.7, 132.3, 127.5, 125.2, 125.1, 120.0, 34.7, 34.0, 31.2, 30.9.

HRMS (ESI-TOF) m/z: $[M+Na]^+$ calcd for $C_{21}H_{28}NO^+$ 310.2165; found: 310.2163.

***N*-(4-(*tert*-butyl)phenyl)-[1,1'-biphenyl]-4-carboxamide**

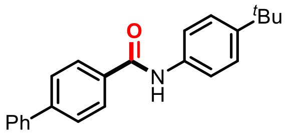

61.7 mg, white solid, yield: 94%.

Rf = 0.3 (silica gel, petroleum ether : ethyl acetate : triethylamine = 10:1:0.01)

^{1}H NMR (400 MHz, DMSO-*d$_6$*) δ 10.23 (s, 1H), 8.07 (d, *J* = 8.6 Hz, 2H), 7.83 (d, *J* = 8.6 Hz, 2H), 7.79 – 7.69 (m, 4H), 7.56 – 7.48 (m, 2H), 7.46 – 7.33 (m, 3H), 1.29 (s, 9H).

^{13}C NMR (101 MHz, DMSO-*d$_6$*) δ 164.9, 146.0, 143.0, 139.1, 136.6, 133.8, 129.1, 128.3, 126.9, 126.5, 125.2, 120.1, 34.1, 31.2.

HRMS (ESI-TOF) m/z: $[M+H]^+$ calcd for $C_{23}H_{24}NO^+$ 330.1852; found: 330.1857.

4-benzyl-*N*-(4-(*tert*-butyl)phenyl)benzamide

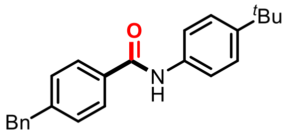

58.6 mg, colorless oil, yield: 85%.

Rf = 0.3 (silica gel, petroleum ether : ethyl acetate = 10:1)

^{1}H NMR (400 MHz, DMSO-*d$_6$*) δ 10.09 (s, 1H), 7.88 (d, *J* = 8.3 Hz, 2H), 7.67 (d, *J* = 8.8 Hz, 2H), 7.41 – 7.17 (m, 9H), 4.02 (s, 2H), 1.27 (s, 9H).

^{13}C NMR (101 MHz, DMSO-*d$_6$*) δ 165.2, 145.9, 145.0, 140.8, 136.6, 132.8, 128.7, 128.6, 128.5, 127.8, 126.1, 125.2, 120.1, 40.9, 34.0, 31.2.

HRMS (ESI-TOF) m/z: $[M+H]^+$ calcd for $C_{24}H_{26}NO^+$ 344.2009; found: 344.2020.

***N*-(4-(*tert*-butyl)phenyl)-4-methoxybenzamide**

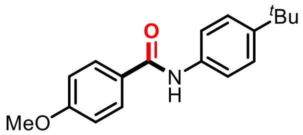

41.2 mg, white solid, yield: 73%.

Rf = 0.3 (silica gel, petroleum ether : ethyl acetate = 6:1)

^{1}H NMR (400 MHz, DMSO-*d$_6$*) δ 10.01 (s, 1H), 7.96 (d, *J* = 8.9 Hz, 2H), 7.68 (d, *J* = 8.8 Hz, 2H), 7.35 (d, *J* = 8.8 Hz, 2H), 7.05 (d, *J* = 8.9 Hz, 2H), 3.83 (s, 3H), 1.28 (s, 9H).

^{13}C NMR (101 MHz, DMSO-*d$_6$*) δ 164.7, 161.8, 145.7, 136.8, 129.5, 127.0, 125.1, 120.1, 113.5, 55.4, 34.0, 31.2.

HRMS (ESI-TOF) m/z: $[M+H]^+$ calcd for $C_{17}H_{13}O_2^+$ 284.1645; found: 284.1650.

4-(*tert*-butoxy)-*N*-(4-(*tert*-butyl)phenyl)benzamide

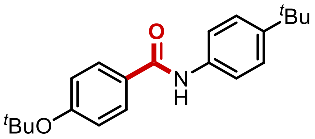

48.6 mg, colorless oil, yield: 75%.

Rf = 0.3 (silica gel, petroleum ether : ethyl acetate = 10:1)

^{1}H NMR (400 MHz, DMSO-*d₆*) δ 10.07 (s, 1H), 7.90 (d, *J* = 8.7 Hz, 2H), 7.68 (d, *J* = 8.8 Hz, 2H), 7.35 (d, *J* = 8.8 Hz, 2H), 7.09 (d, *J* = 8.7 Hz, 2H), 1.36 (s, 9H), 1.28 (s, 9H).

^{13}C NMR (101 MHz, DMSO-d_6) δ 164.9, 158.2, 145.8, 136.7, 129.2, 128.9, 125.2, 122.3, 120.1, 78.8, 34.0, 31.2, 28.5.

HRMS (ESI-TOF) m/z: $[M+H]^+$ calcd for $C_{17}H_{20}NO_2^+$ 270.1489; found: 270.1495.

***N*-(4-(*tert*-butyl)phenyl)-4-(dimethylamino)benzamide**

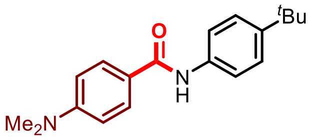

37.8 mg, white solid, yield: 64%.

Rf = 0.3 (silica gel, petroleum ether : ethyl acetate = 4:1)

^{1}H NMR (400 MHz, DMSO-d_6) δ 9.79 (s, 1H), 7.87 (d, *J* = 9.0 Hz, 2H), 7.68 (d, *J* = 8.8 Hz, 2H), 7.33 (d, *J* = 8.8 Hz, 2H), 6.75 (d, *J* = 9.2 Hz, 2H), 2.99 (s, 6H), 1.27 (s, 9H).

^{13}C NMR (101 MHz, DMSO-d_6) δ 165.0, 152.3, 145.2, 137.1, 129.1, 125.0, 121.2, 120.0, 110.7, 39.7, 34.0, 31.2.

HRMS (ESI-TOF) m/z: $[M+H]^+$ calcd for $C_{19}H_{25}N_2O^+$ 297.1961; found: 297.1973.

4-acetyl-*N*-(4-(*tert*-butyl)phenyl)benzamide

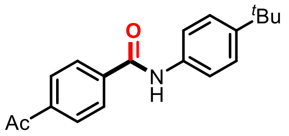

42.3 mg, white solid, yield: 72%.

Rf = 0.3 (silica gel, petroleum ether : ethyl acetate = 3:1)

^{1}H NMR (400 MHz, DMSO-*d$_6$*) δ 10.35 (s, 1H), 8.08 (s, 4H), 7.70 (d, *J* = 8.8 Hz, 2H), 7.38 (d, *J* = 8.8 Hz, 2H), 2.64 (s, 3H), 1.28 (s, 9H).

^{13}C NMR (101 MHz, DMSO-*d$_6$*) δ 197.7, 164.5, 146.3, 138.8, 138.8, 136.3, 128.2, 128.0, 125.3, 120.2, 34.1, 31.2, 27.0.

HRMS (ESI-TOF) m/z: $[M+H]^+$ calcd for $C_{19}H_{22}NO_2^+$ 296.1645; found: 296.1653.

***N*-(4-(*tert*-butyl)phenyl)-4-(trifluoromethyl)benzamide**

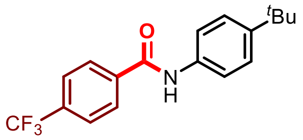

46.7 mg, white solid, yield: 73%.

Rf = 0.3 (silica gel, petroleum ether : ethyl acetate = 15:1)

^{1}H NMR (400 MHz, DMSO-*d$_6$*) δ 10.40 (s, 1H), 8.18 – 8.11 (m, 2H), 7.90 (d, *J* = 8.1 Hz, 2H), 7.70 (d, *J* = 8.8 Hz, 2H), 7.38 (d, *J* = 8.8 Hz, 2H), 1.28 (s, 9H).

^{13}C NMR (101 MHz, DMSO-*d$_6$*) δ 164.2, 146.4, 138.8, 136.3, 131.3 (q, J_{C-F} = 31.9 Hz), 128.6, 125.4 (q, J_{C-F} = 3.7 Hz), 125.3, 124.0 (q, J_{C-F} = 272.2 Hz), 120.2, 34.1, 31.2.

^{19}F NMR (376 MHz, DMSO-*d$_6$*) δ -61.34.

HRMS (ESI-TOF) m/z: $[M+H]^+$ calcd for $C_{18}H_{19}F_3NO^+$ 322.1413; found: 322.1414.

***N*-(4-(*tert*-butyl)phenyl)-3-methylbenzamide**

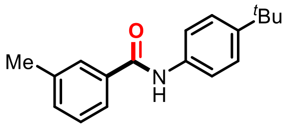

46.7 mg, colorless oil, yield: 87%.

Rf = 0.4 (silica gel, petroleum ether : ethyl acetate = 10:1)

^{1}H NMR (400 MHz, DMSO-*d*$_6$) δ 10.13 (s, 1H), 7.80 – 7.65 (m, 4H), 7.44 – 7.31 (m, 4H), 2.40 (s, 3H), 1.28 (s, 9H).

^{13}C NMR (101 MHz, DMSO-*d*$_6$) δ 165.4, 145.9, 137.6, 136.6, 135.0, 132.0, 128.2, 128.1, 125.2, 124.8, 120.1, 34.0, 31.2, 21.0.

HRMS (ESI-TOF) m/z: $[M+H]^+$ calcd for $C_{18}H_{22}NO^+$ 268.1696; found: 268.1704.

***N*-(4-(*tert*-butyl)phenyl)-3-methoxybenzamide**

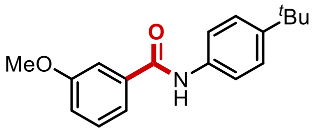

42.6 mg, yellow oil, yield: 75%.

Rf = 0.3 (silica gel, petroleum ether : ethyl acetate = 10:1)

^{1}H NMR (400 MHz, DMSO-*d*$_6$) δ 10.15 (s, 1H), 7.69 (d, *J* = 8.8 Hz, 2H), 7.54 (dt, *J* = 7.7, 1.3 Hz, 1H), 7.49 (dd, *J* = 2.7, 1.6 Hz, 1H), 7.44 (t, *J* = 7.9 Hz, 1H), 7.36 (d, *J* = 8.8 Hz, 2H), 7.15 (ddd, *J* = 8.2, 2.6, 1.0 Hz, 1H), 3.84 (s, 3H), 1.28 (s, 9H).

^{13}C NMR (101 MHz, DMSO-*d*$_6$) δ 165.0, 159.2, 146.0, 136.5, 136.4, 129.5, 125.2, 120.2, 119.8, 117.2, 112.9, 55.3, 34.1, 31.2.

HRMS (ESI-TOF) m/z: $[M+H]^+$ calcd for $C_{18}H_{22}NO_2^+$ 284.1645; found: 284.1652.

3-acetyl-*N*-(4-(*tert*-butyl)phenyl)benzamide

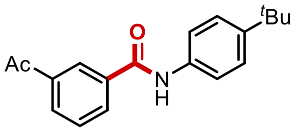

37.7 mg, white solid, yield: 64%.

Rf = 0.3 (silica gel, petroleum ether : ethyl acetate = 4:1)

^{1}H NMR (400 MHz, DMSO-*d$_6$*) δ 10.37 (s, 1H), 8.50 (s, 1H), 8.26 – 8.12 (m, 2H), 7.79 – 7.63 (m, 3H), 7.38 (d, *J* = 8.8 Hz, 2H), 2.67 (s, 3H), 1.29 (s, 9H).

^{13}C NMR (101 MHz, DMSO-*d$_6$*) δ 197.6, 164.6, 146.2, 136.8, 136.4, 135.4, 132.2, 131.0, 128.9, 127.2, 125.2, 120.3, 34.1, 31.2, 26.9.

HRMS (ESI-TOF) m/z: $[M+H]^+$ calcd for $C_{19}H_{22}NO_2^+$ 296.1645; found: 296.1651.

N-(4-(tert-butyl)phenyl)-2-methylbenzamide

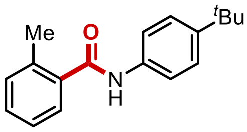

41.9 mg, colorless oil, yield: 78%.

Rf = 0.3 (silica gel, petroleum ether : ethyl acetate = 12:1)

^{1}H NMR (400 MHz, DMSO-*d$_6$*) δ 10.21 (s, 1H), 7.67 (d, *J* = 8.7 Hz, 2H), 7.47 – 7.41 (m, 1H), 7.41 – 7.32 (m, 3H), 7.32 – 7.25 (m, 2H), 2.38 (s, 3H), 1.28 (s, 9H).

^{13}C NMR (101 MHz, DMSO-*d$_6$*) δ 167.7, 145.8, 137.4, 136.7, 135.1, 130.5, 129.5, 127.1, 125.6, 125.2, 119.4, 34.0, 31.2, 19.3.

HRMS (ESI-TOF) m/z: $[M+H]^+$ calcd for $C_{18}H_{22}NO^+$ 268.1696; found: 268.1700.

3,5-dimethoxy-*N*-phenylbenzamide

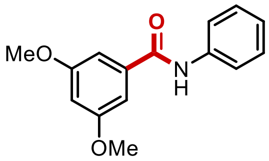

31.6 mg, yellow oil, yield: 61%.

Rf = 0.3 (silica gel, petroleum ether : ethyl acetate = 7:1)

^{1}H NMR (400 MHz, DMSO-*d$_6$*) δ 10.17 (s, 1H), 7.85 – 7.70 (m, 2H), 7.35 (dd, *J* = 8.6, 7.4 Hz, 2H), 7.11 (d, *J* = 2.4 Hz, 3H), 6.72 (s, 1H), 3.82 (s, 6H).

^{13}C NMR (101 MHz, DMSO-*d$_6$*) δ 165.1, 160.4, 139.0, 137.0, 128.6, 123.8, 120.5, 105.6, 103.3, 55.5.

HRMS (ESI-TOF) m/z: $[M+H]^+$ calcd for $C_{15}H_{16}NO_3^+$ 258.1125; found: 258.1126.

***N*-(4-(*tert*-butyl)phenyl)-2-naphthamide**

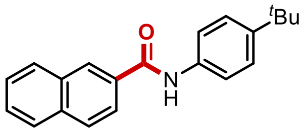

55.6 mg, colorless oil, yield: 92%.

Rf = 0.3 (silica gel, petroleum ether : ethyl acetate = 10:1)

^{1}H NMR (400 MHz, DMSO-*d$_6$*) δ 10.37 (s, 1H), 8.58 (d, *J* = 1.2 Hz, 1H), 8.14 – 7.98 (m, 4H), 7.75 (d, *J* = 8.7 Hz, 2H), 7.72 – 7.58 (m, 2H), 7.39 (d, *J* = 8.8 Hz, 2H), 1.29 (s, 9H).

^{13}C NMR (101 MHz, DMSO-*d$_6$*) δ 165.8, 146.5, 137.1, 134.7, 132.8, 132.6, 129.4, 128.4, 128.4, 128.2, 128.1, 127.3, 125.7, 124.9, 120.6, 34.5, 31.7.

HRMS (ESI-TOF) m/z: $[M+H]^+$ calcd for $C_{21}H_{22}NO^+$ 304.1696; found: 304.1702.

***N*-(4-(*tert*-butyl)phenyl)benzo[*d*][1,3]dioxole-5-carboxamide**

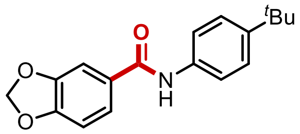

44.1 mg, colorless oil, yield: 74%.

Rf = 0.3 (silica gel, petroleum ether : ethyl acetate = 6:1)

^{1}H NMR (700 MHz, DMSO-*d$_6$*) δ 9.99 (s, 1H), 7.66 (d, *J* = 8.8 Hz, 2H), 7.57 (dd, *J* = 8.1, 1.9 Hz, 1H), 7.51 (d, *J* = 1.9 Hz, 1H), 7.35 (d, *J* = 8.7 Hz, 2H), 7.05 (d, *J* = 8.1 Hz, 1H), 6.13 (s, 2H), 1.27 (s, 9H).

^{13}C NMR (176 MHz, DMSO-*d$_6$*) δ 164.3, 150.0, 147.4, 145.8, 136.6, 128.8, 125.2, 122.8, 120.1, 107.9, 107.7, 101.8, 34.0, 31.2.

HRMS (ESI-TOF) m/z: $[M+H]^+$ calcd for $C_{18}H_{20}NO_3^+$ 298.1438; found: 298.1438.

***N*-phenylquinoline-6-carboxamide**

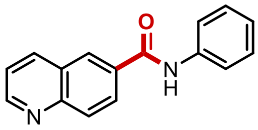

25.5 mg, white solid, yield: 51%.

Rf = 0.3 (silica gel, petroleum ether : ethyl acetate: triethylamine = 1:1:0.01)

^{1}H NMR (700 MHz, DMSO-*d*$_6$) δ 10.51 (s, 1H), 9.02 (dd, *J* = 4.2, 1.7 Hz, 1H), 8.64 (d, *J* = 2.2 Hz, 1H), 8.54 (dt, *J* = 8.3, 1.4 Hz, 1H), 8.26 (dd, *J* = 8.8, 2.1 Hz, 1H), 8.14 (d, *J* = 8.7 Hz, 1H), 7.86 – 7.79 (m, 2H), 7.64 (dd, *J* = 8.2, 4.1 Hz, 1H), 7.39 (dd, *J* = 8.5, 7.4 Hz, 2H), 7.13 (tt, *J* = 7.4, 1.2 Hz, 1H).

^{13}C NMR (176 MHz, DMSO-*d*$_6$) δ 165.1, 152.3, 148.8, 139.1, 137.2, 132.8, 129.1, 128.7, 128.4, 128.1, 127.1, 123.8, 122.3, 120.4.

HRMS (ESI-TOF) m/z: $[M+H]^+$ calcd for $C_{16}H_{13}N_2O^+$ 249.1022; found: 249.1023.

***N*-(4-(*tert*-butyl)phenyl)-4-morpholinobenzamide**

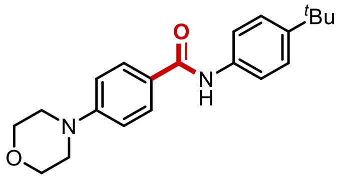

40.9 mg, white solid, yield: 60%.

Rf = 0.3 (silica gel, petroleum ether : ethyl acetate = 3:1)

^{1}H NMR (700 MHz, DMSO-*d*$_6$) δ 9.89 (s, 1H), 7.89 (d, *J* = 9.0 Hz, 2H), 7.67 (d, *J* = 8.7 Hz, 2H), 7.33 (d, *J* = 8.7 Hz, 2H), 7.02 (d, *J* = 9.1 Hz, 2H), 3.75 (t, *J* = 4.9 Hz, 4H), 3.24 (dd, *J* = 5.8, 4.1 Hz, 4H), 1.27 (s, 9H).

^{13}C NMR (176 MHz, DMSO-*d*$_6$) δ 164.8, 153.1, 145.5, 136.9, 129.0, 125.1, 120.0, 113.3, 65.9, 47.3, 34.0, 31.2.

HRMS (ESI-TOF) m/z: $[M+H]^+$ calcd for $C_{21}H_{25}N_2O_2^+$ 339.2067; found: 339.2070.

***N*-phenylpyrazine-2-carboxamide**

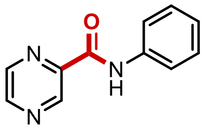

18.4 mg, white solid, yield: 46%.

Rf = 0.3 (silica gel, petroleum ether : ethyl acetate = 3:1)

^{1}H NMR (700 MHz, DMSO-*d*$_6$) δ 9.47 (s, 1H), 8.22 (d, *J* = 1.5 Hz, 1H), 8.11 (dd, *J* = 2.8, 1.5 Hz, 1H), 7.91 (d, *J* = 2.7 Hz, 1H), 7.76 – 7.61 (m, 2H), 7.30 (dd, *J* = 8.7, 7.3 Hz, 2H), 7.01 – 6.90 (m, 1H).

^{13}C NMR (176 MHz, DMSO-*d*$_6$) δ 152.8, 141.5, 141.1, 135.5, 134.0, 129.2, 121.9, 118.7.

HRMS (ESI-TOF) m/z: [M+H]$^+$ calcd for $C_{11}H_{10}N_3O^+$ 200.0818; found: 200.0010.

4-((((1R,2S,5R)-2-isopropyl-5-methylcyclohexyl)oxy)methyl)-*N*-phenylbenzamide

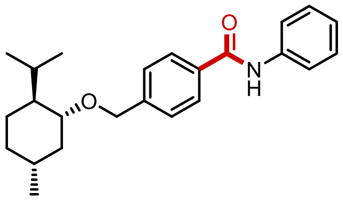

47.5 mg, white solid, yield: 65%.

Rf = 0.3 (silica gel, petroleum ether : ethyl acetate = 8:1)

^{1}H NMR (700 MHz, DMSO-*d*$_6$) δ 10.20 (s, 1H), 7.94 (d, *J* = 8.3 Hz, 2H), 7.79 (d, *J* = 7.4 Hz, 2H), 7.46 (d, *J* = 8.3 Hz, 2H), 7.35 (dd, *J* = 8.5, 7.4 Hz, 2H), 7.13 – 7.06 (m, 1H), 4.69 (d, *J* = 12.5 Hz, 1H), 4.43 (d, *J* = 12.5 Hz, 1H), 3.17 (td, *J* = 10.5, 4.1 Hz, 1H), 2.21 (tt, *J* = 10.5, 2.4 Hz, 2H), 1.60 (ddt, *J* = 40.0, 13.1, 3.3 Hz, 2H), 1.35 (tdq, *J* = 9.8, 6.8, 3.3 Hz, 1H), 1.22 (ddt, *J* = 13.0, 10.2, 3.2 Hz, 1H), 0.98 – 0.89 (m, 4H), 0.88 – 0.81 (m, 5H), 0.70 (d, *J* = 6.9 Hz, 3H).

^{13}C NMR (176 MHz, DMSO-*d*$_6$) δ 165.3, 143.0, 139.2, 133.8, 128.6, 127.6, 127.2, 123.6, 120.3, 78.2, 68.9, 47.9, 39.9, 34.1, 30.9, 25.3, 23.0, 22.3, 20.8, 16.2.

HRMS (ESI-TOF) m/z: [M+Na]$^+$ calcd for $C_{24}H_{32}NO_2^+$ 366.2428; found: 366.2423.

***N*-(*p*-tolyl)benzamide**

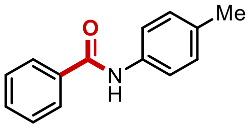

36.9 mg, white solid, yield: 87%.

Rf = 0.3 (silica gel, petroleum ether : ethyl acetate = 8:1)

^{1}H NMR (700 MHz, DMSO-*d*$_6$) δ 10.16 (s, 1H), 7.94 (d, *J* = 7.0 Hz, 2H), 7.66 (d, *J* = 8.5 Hz, 2H), 7.60 – 7.56 (m, 1H), 7.52 (dd, *J* = 8.3, 6.8 Hz, 2H), 7.15 (d, *J* = 8.4 Hz, 2H), 2.28 (s, 3H).

^{13}C NMR (176 MHz, DMSO-*d*$_6$) δ 165.3, 136.7, 135.1, 132.6, 131.5, 129.0, 128.4, 127.6, 120.4, 20.5.

HRMS (ESI-TOF) m/z: $[M+H]^+$ calcd for $C_{14}H_{14}NO^+$ 212.1070; found: 212.1073.

***N*-(*m*-tolyl)benzamide**

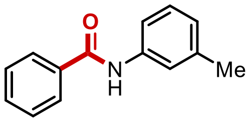

36.7 mg, white solid, yield: 87%.

Rf = 0.3 (silica gel, petroleum ether : ethyl acetate = 8:1)

^{1}H NMR (700 MHz, DMSO-*d*$_6$) δ 10.17 (s, 1H), 7.99 – 7.91 (m, 2H), 7.63 (d, *J* = 2.2 Hz, 1H), 7.60 – 7.56 (m, 2H), 7.53 (dd, *J* = 8.3, 6.8 Hz, 2H), 7.23 (t, *J* = 7.8 Hz, 1H), 6.96 – 6.87 (m, 1H), 2.31 (s, 3H).

^{13}C NMR (176 MHz, DMSO-*d*$_6$) δ 165.5, 139.1, 137.7, 135.0, 131.5, 128.4, 128.4, 127.6, 124.4, 120.9, 117.6, 21.2.

HRMS (ESI-TOF) m/z: $[M+H]^+$ calcd for $C_{14}H_{14}NO^+$ 212.1070; found: 212.1071.

***N*-(*o*-tolyl)benzamide**

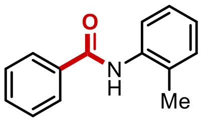

31.2 mg, white solid, yield: 74%.

Rf = 0.3 (silica gel, petroleum ether : ethyl acetate = 7:1)

^{1}H NMR (700 MHz, DMSO-*d*$_6$) δ 9.88 (s, 1H), 8.03 – 7.95 (m, 2H), 7.62 – 7.57 (m,

1H), 7.53 (dd, J = 8.4, 6.9 Hz, 2H), 7.37 – 7.32 (m, 1H), 7.28 (dd, J = 7.5, 1.5 Hz, 1H), 7.22 (td, J = 7.6, 1.8 Hz, 1H), 7.17 (td, J = 7.5, 1.6 Hz, 1H), 2.24 (s, 3H).

^{13}C NMR (176 MHz, DMSO-*d*$_6$) δ 165.3, 136.4, 134.5, 133.7, 131.5, 130.3, 128.4, 127.6, 126.6, 126.0, 17.9.

HRMS (ESI-TOF) m/z: $[M+H]^+$ calcd for $C_{14}H_{14}NO^+$ 212.1070; found: 212.1072.

***N*-(3-(*tert*-butyl)phenyl)benzamide**

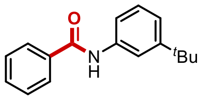

39.8 mg, yellow oil, yield: 79%.

Rf = 0.3 (silica gel, petroleum ether : ethyl acetate = 10:1)

^{1}H NMR (700 MHz, DMSO-*d*$_6$) δ 10.18 (s, 1H), 8.02 – 7.94 (m, 2H), 7.80 (t, J = 2.1 Hz, 1H), 7.69 (dd, J = 8.0, 2.7 Hz, 1H), 7.60 – 7.57 (m, 1H), 7.53 (dd, J = 8.4, 6.8 Hz, 2H), 7.27 (t, J = 7.9 Hz, 1H), 7.17 – 7.11 (m, 1H), 1.29 (s, 9H).

^{13}C NMR (176 MHz, DMSO-*d*$_6$) δ 165.9, 151.6, 139.4, 135.5, 132.0, 128.8, 128.7, 128.1, 121.1, 118.0, 117.9, 34.9, 31.6.

HRMS (ESI-TOF) m/z: $[M+H]^+$ calcd for $C_{17}H_{20}NO^+$ 254.1539; found: 254.1542.

***N*-(2,5-dimethylphenyl)benzamide**

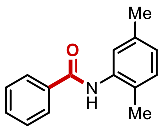

33.5 mg, white solid, yield: 74%.

Rf = 0.3 (silica gel, petroleum ether : ethyl acetate = 9:1)

^{1}H NMR (700 MHz, DMSO-*d*$_6$) δ 9.82 (s, 1H), 8.06 – 7.91 (m, 2H), 7.62 – 7.56 (m, 1H), 7.52 (dd, J = 8.3, 6.8 Hz, 2H), 7.21 – 7.12 (m, 2H), 7.02 – 6.91 (m, 1H), 2.28 (s, 3H), 2.19 (s, 3H).

^{13}C NMR (176 MHz, DMSO-*d*$_6$) δ 165.3, 136.2, 135.0, 134.6, 131.5, 130.5, 130.1, 128.4, 127.6, 127.1, 126.7, 20.5, 17.5.

HRMS (ESI-TOF) m/z: $[M+H]^+$ calcd for $C_{15}H_{16}NO^+$ 226.1226; found: 226.1230.

***N*-(4-methoxyphenyl)benzamide**

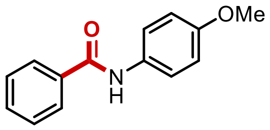

30.0 mg, white solid, yield: 66%.

Rf = 0.3 (silica gel, petroleum ether : ethyl acetate = 4:1)

^{1}H NMR (700 MHz, DMSO-*d*$_{6}$) δ 10.13 (s, 1H), 8.00 – 7.90 (m, 2H), 7.68 (d, *J* = 9.0 Hz, 2H), 7.60 – 7.55 (m, 1H), 7.52 (dd, *J* = 8.2, 6.7 Hz, 2H), 6.93 (d, *J* = 9.1 Hz, 2H), 3.74 (s, 3H).

^{13}C NMR (176 MHz, DMSO-*d*$_{6}$) δ 165.1, 155.6, 135.1, 132.2, 131.4, 128.4, 127.5, 122.0, 113.7, 55.2.

HRMS (ESI-TOF) m/z: $[M+H]^+$ calcd for $C_{14}H_{14}NO_2^+$ 228.1019; found: 228.1970.

***N*-(3-methoxyphenyl)benzamide**

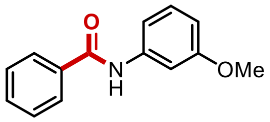

33.6 mg, yellow oil, yield: 74%.

Rf = 0.3 (silica gel, petroleum ether : ethyl acetate = 8:1)

^{1}H NMR (700 MHz, DMSO-*d*$_{6}$) δ 10.22 (s, 1H), 7.99 – 7.90 (m, 2H), 7.61 – 7.57 (m, 1H), 7.53 (dd, *J* = 8.2, 6.8 Hz, 2H), 7.49 (t, *J* = 2.3 Hz, 1H), 7.38 (ddd, *J* = 8.2, 2.0, 0.8 Hz, 1H), 7.25 (t, *J* = 8.1 Hz, 1H), 6.69 (ddd, *J* = 8.2, 2.6, 0.9 Hz, 1H), 3.75 (s, 3H).

^{13}C NMR (176 MHz, DMSO-*d*$_{6}$) δ 165.6, 159.4, 140.4, 135.0, 131.6, 129.4, 128.4, 127.7, 112.6, 109.2, 106.0, 55.0.

HRMS (ESI-TOF) m/z: $[M+H]^+$ calcd for $C_{14}H_{14}NO_2^+$ 228.1019; found: 228.1021.

***N*-(4-acetylphenyl)benzamide**

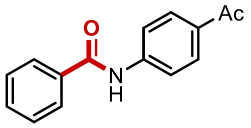

38.1 mg, white solid, yield: 80%.

Rf = 0.3 (silica gel, petroleum ether : ethyl acetate = 3:1)

^{1}H NMR (700 MHz, DMSO-*d*$_6$) δ 10.56 (s, 1H), 8.04 – 7.92 (m, 6H), 7.62 (t, *J* = 7.4 Hz, 1H), 7.55 (t, *J* = 7.7 Hz, 2H), 2.55 (s, 3H).

^{13}C NMR (176 MHz, DMSO-*d*$_6$) δ 196.6, 166.0, 143.6, 134.6, 132.0, 131.9, 129.3, 128.5, 127.8, 119.4, 26.5.

HRMS (ESI-TOF) m/z: $[M+H]^+$ calcd for $C_{15}H_{14}NO_2^+$ 240.1019; found: 240.1022.

_N_-(4-fluorophenyl)benzamide

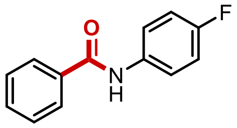

34.2 mg, white solid, yield: 0%.

Rf = 0.3 (silica gel, petroleum ether : ethyl acetate = 8:1)

^{1}H NMR (400 MHz, CDCl$_3$) δ 10.30 (s, 1H), 7.98 – 7.92 (m, 2H), 7.83 – 7.76 (m, 2H), 7.61 – 7.58 (m, 1H), 7.53 (dd, *J* = 8.3, 6.8 Hz, 2H), 7.19 (t, *J* = 8.9 Hz, 2H).

^{13}C NMR (101 MHz, CDCl$_3$) δ 165.5, 158.3 (d, J_{C-F} = 239.9 Hz), 135.5 (d, J_{C-F} = 2.6 Hz), 134.8, 131.6, 128.4, 127.6, 122.2 (d, J_{C-F} = 7.8 Hz), 115.3 (d, J_{C-F} = 22.1 Hz).

^{19}F NMR (376 MHz, CDCl$_3$) δ -118.92.

HRMS (ESI-TOF) m/z: $[M+H]^+$ calcd for $C_{13}H_{11}FNO^+$ 216.0819; found: 216.0820.

_N_-(4-(trifluoromethyl)phenyl)benzamide

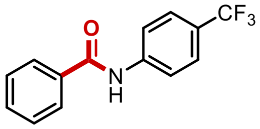

36.5 mg, white solid, yield: 69%.

Rf = 0.3 (silica gel, petroleum ether : dichloromethane = 9:1)

^{1}H NMR (400 MHz, CDCl$_3$) δ 10.58 (s, 1H), 8.02 (d, *J* = 8.7 Hz, 2H), 7.99 – 7.95 (m, 2H), 7.73 (d, *J* = 8.8 Hz, 2H), 7.64 – 7.60 (m, 1H), 7.55 (t, *J* = 7.7 Hz, 2H).

^{13}C NMR (101 MHz, CDCl$_3$) δ 166.5, 143.3, 135.0, 132.4, 128.9, 128.3, 126.4 (q, J_{C-F} = 3.9 Hz), 125.6 (q, J_{C-F} = 271.2 Hz), 124.2 (q, J_{C-F} = 32.7 Hz), 120.6.

^{19}F NMR (376 MHz, CDCl$_3$) δ -60.32.

HRMS (ESI-TOF) m/z: $[M+H]^+$ calcd for $C_{14}H_{11}F_3NO^+$ 266.0787; found: 266.0791.

***N*-(4-(trifluoromethoxy)phenyl)benzamide**

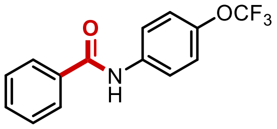

42.1 mg, white solid, yield: 75%.

Rf = 0.3 (silica gel, petroleum ether : dichloromethane = 9:1)

^{1}H NMR (400 MHz, CDCl$_3$) δ 10.44 (s, 1H), 7.96 (dd, *J* = 8.2, 1.3 Hz, 2H), 7.90 (d, *J* = 9.1 Hz, 2H), 7.62 – 7.59 (m, 1H), 7.54 (t, *J* = 7.6 Hz, 2H), 7.36 (d, *J* = 9.0 Hz, 2H).

^{13}C NMR (101 MHz, CDCl$_3$) δ 166.2, 144.3, 138.9, 135.1, 132.2, 128.9, 128.2, 122.1, 121.9, 120.6 (q, $J_{C\text{-}F}$ = 255.5 Hz).

^{19}F NMR (376 MHz, CDCl$_3$) δ -57.03.

HRMS (ESI-TOF) m/z: [M+H]$^+$ calcd for $C_{14}H_{11}F_3NO_2^+$ 282.0736; found: 282.1846.

***N*-(naphthalen-1-yl)benzamide**

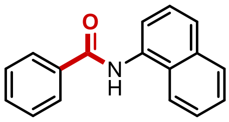

41.4 mg, white solid, yield: 84%.

Rf = 0.3 (silica gel, petroleum ether : ethyl acetate = 8:1)

^{1}H NMR (700 MHz, DMSO-*d*$_6$) δ 10.44 (s, 1H), 8.13 – 8.08 (m, 2H), 8.03 – 7.96 (m, 2H), 7.87 (d, *J* = 8.2 Hz, 1H), 7.65 – 7.60 (m, 2H), 7.59 – 7.52 (m, 5H).

^{13}C NMR (176 MHz, DMSO-*d*$_6$) δ 166.7, 134.9, 134.3, 134.2, 132.1, 129.7, 128.9, 128.6, 128.3, 126.8, 126.5, 126.5, 126.0, 124.4, 123.8.

HRMS (ESI-TOF) m/z: [M+H]$^+$ calcd for $C_{17}H_{14}NO^+$ 248.1070; found: 248.1066.

***N*-(naphthalen-2-yl)benzamide**

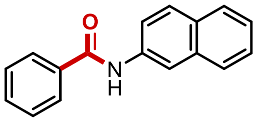

28.0 mg, white solid, yield: 57%.

Rf = 0.3 (silica gel, petroleum ether : ethyl acetate = 8:1)

^{1}H NMR (700 MHz, DMSO-*d*$_6$) δ 10.46 (s, 1H), 8.47 (d, *J* = 2.3 Hz, 1H), 8.04 – 7.99

(m, 2H), 7.90 (d, *J* = 8.8 Hz, 1H), 7.88 – 7.81 (m, 3H), 7.64 – 7.59 (m, 1H), 7.58 – 7.54 (m, 2H), 7.49 (ddd, *J* = 8.2, 6.8, 1.3 Hz, 1H), 7.43 (ddd, *J* = 8.1, 6.8, 1.2 Hz, 1H).

^{13}C NMR (176 MHz, DMSO-*d*$_6$) δ 166.3, 137.3, 135.4, 133.8, 132.1, 130.5, 128.9, 128.6, 128.2, 127.9, 127.9, 126.9, 125.3, 121.4, 117.0.

HRMS (ESI-TOF) m/z: $[M+H]^+$ calcd for $C_{17}H_{14}NO^+$ 248.1070; found: 248.1067.

X-ray Crystallographic Data

Inositol hexaformate: (The crystal structure of **HFI** has been deposited at the Cambridge Crystallographic Data Centre (CCDC 2393156).

Table S10. Crystal data and structure refinement for Inositol hexaformate.

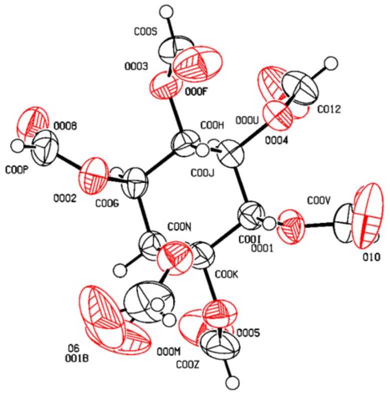

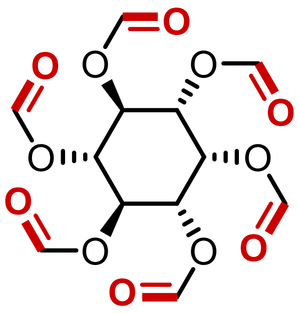

Identification code	LZW
Empirical formula	$C_{12}H_{12}O_{12}$
Formula weight	348.22
Temperature/K	293(2)
Crystal system	monoclinic
Space group	$P2_1/n$
a/Å	15.7617(7)
b/Å	12.2724(5)
c/Å	15.9680(8)
α/°	90
β/°	105.204(5)
γ/°	90
Volume/Å^3	2980.6(2)
Z	8
ρ_{calc}g/cm^3	1.552
μ/mm^{-1}	1.271
F(000)	1440.0
Crystal size/mm^3	? × ? × ?
Radiation	CuKα (λ = 1.54178)
2Θ range for data collection/°	9.212 to 133.97
Index ranges	$-18 \le h \le 18$, $-14 \le k \le 13$, $-17 \le l \le 19$
Reflections collected	19876
Independent reflections	5209 [R_{int} = 0.0303, R_{sigma} = 0.0218]

Data/restraints/parameters	5209/12/470
Goodness-of-fit on F^2	1.071
Final R indexes [I>=2σ (I)]	R_1 = 0.0706, wR_2 = 0.1940
Final R indexes [all data]	R_1 = 0.0824, wR_2 = 0.2057
Largest diff. peak/hole / e $Å^{-3}$	0.39/-0.45

NMR Data

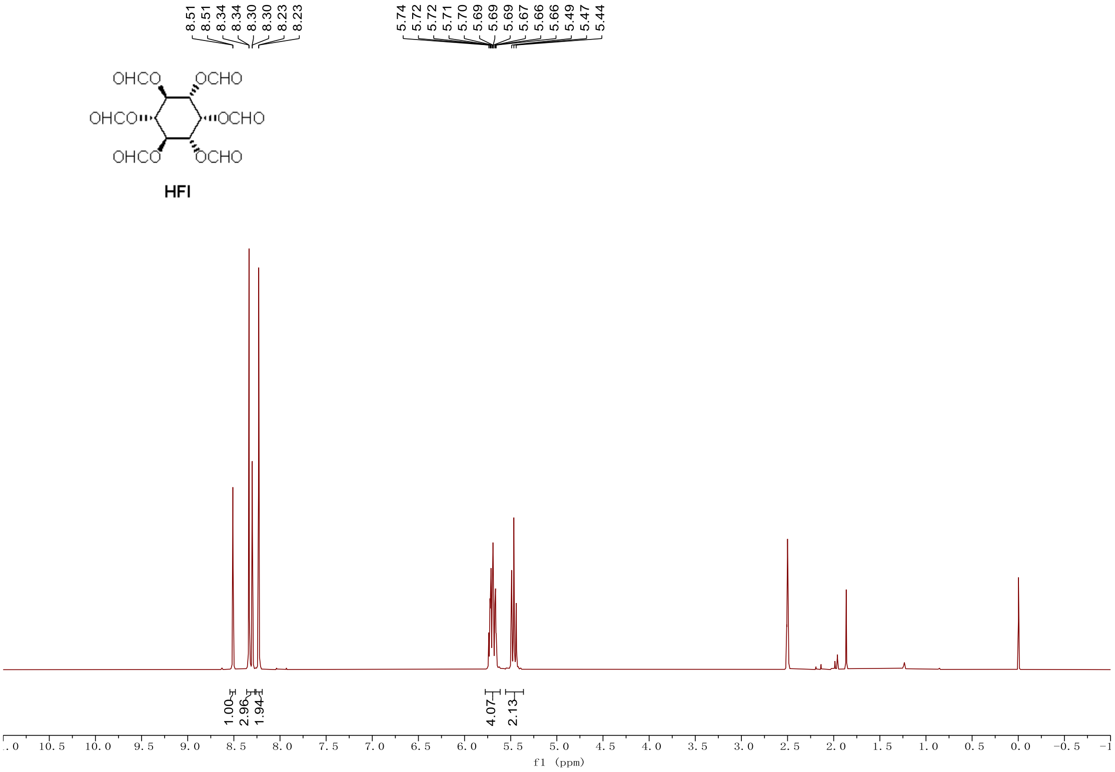

^{1}H NMR spectrum of **HFI** in DMSO-d_6 (400 MHz)

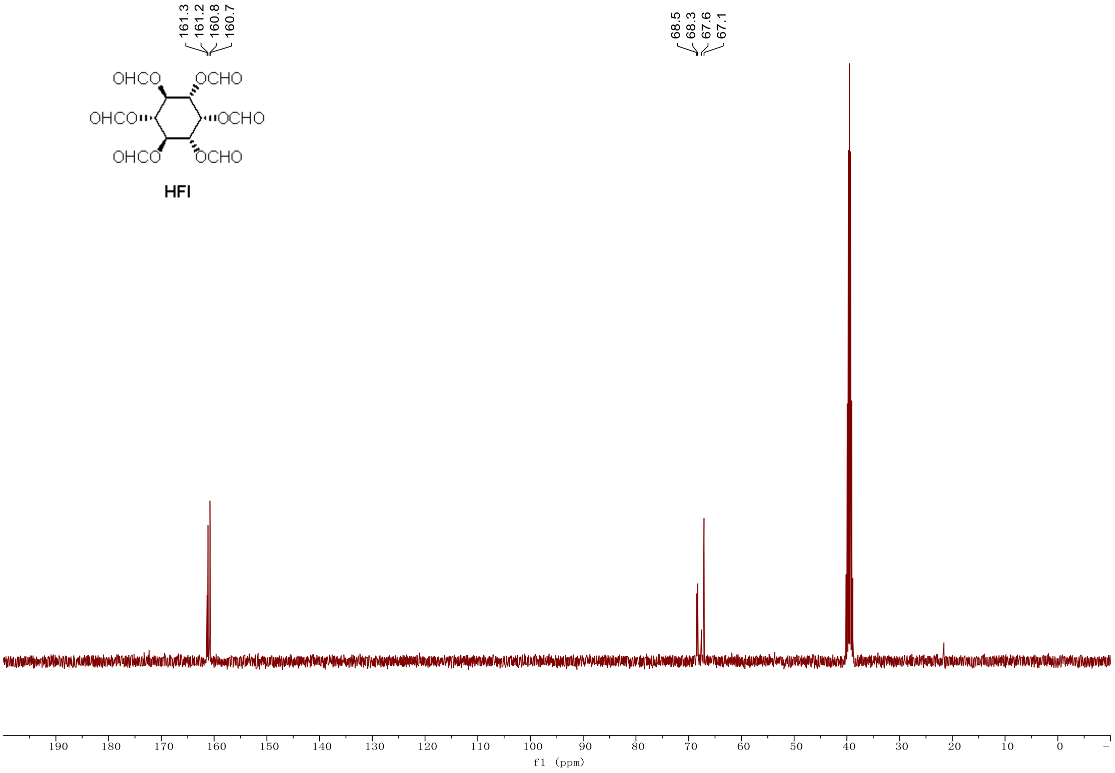

^{13}C NMR spectrum of **HFI** in DMSO-d_6 (101 MHz)

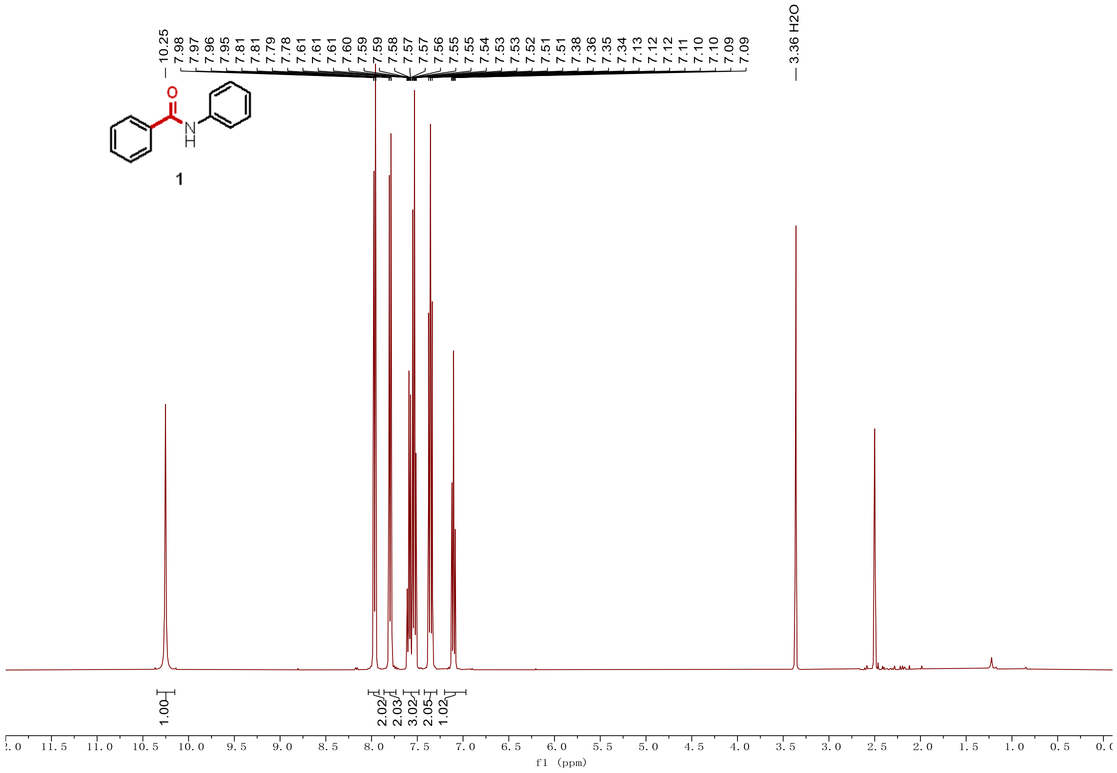

^{1}H NMR spectrum of **1** in DMSO-d_6 (400 MHz)

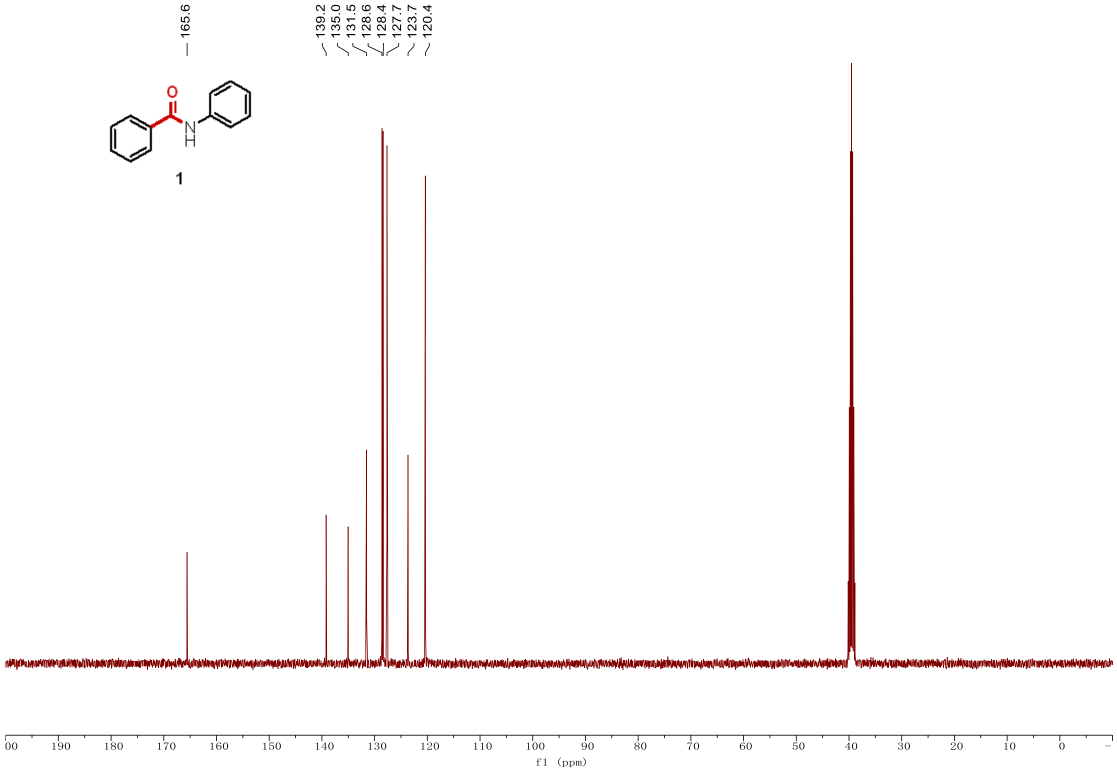

^{13}C NMR spectrum of **1** in DMSO-d_6 (101 MHz)

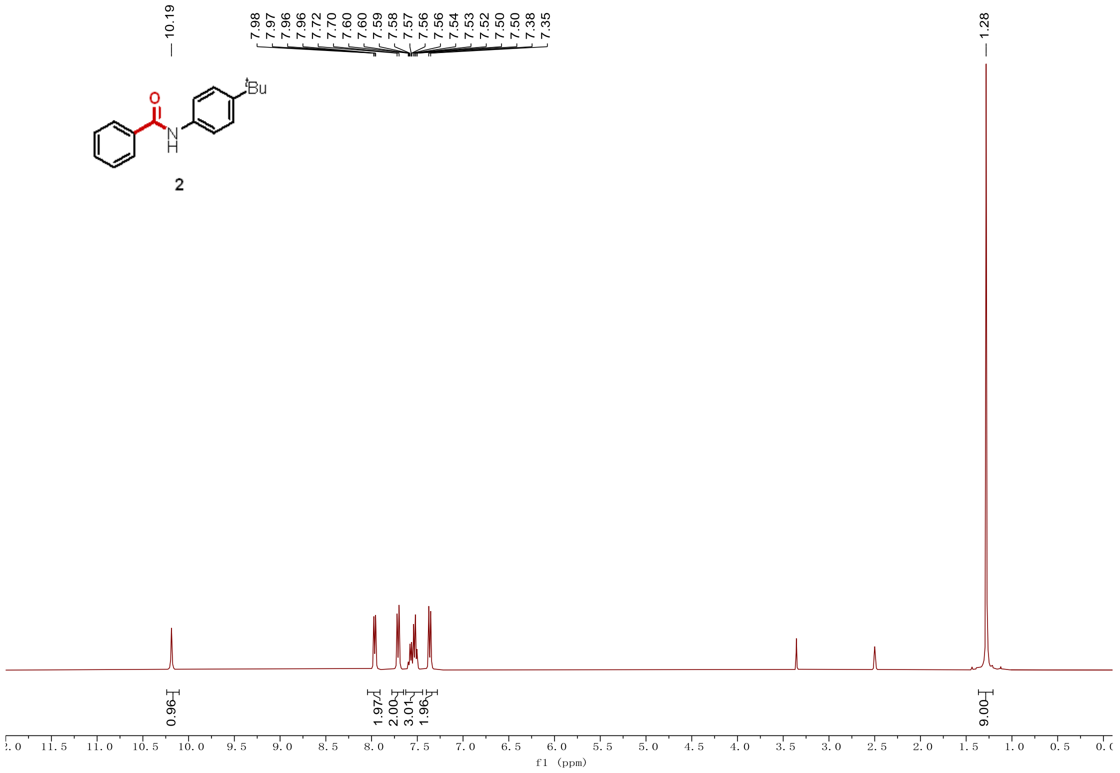

^{1}H NMR spectrum of **2** in DMSO-d_6 (400 MHz)

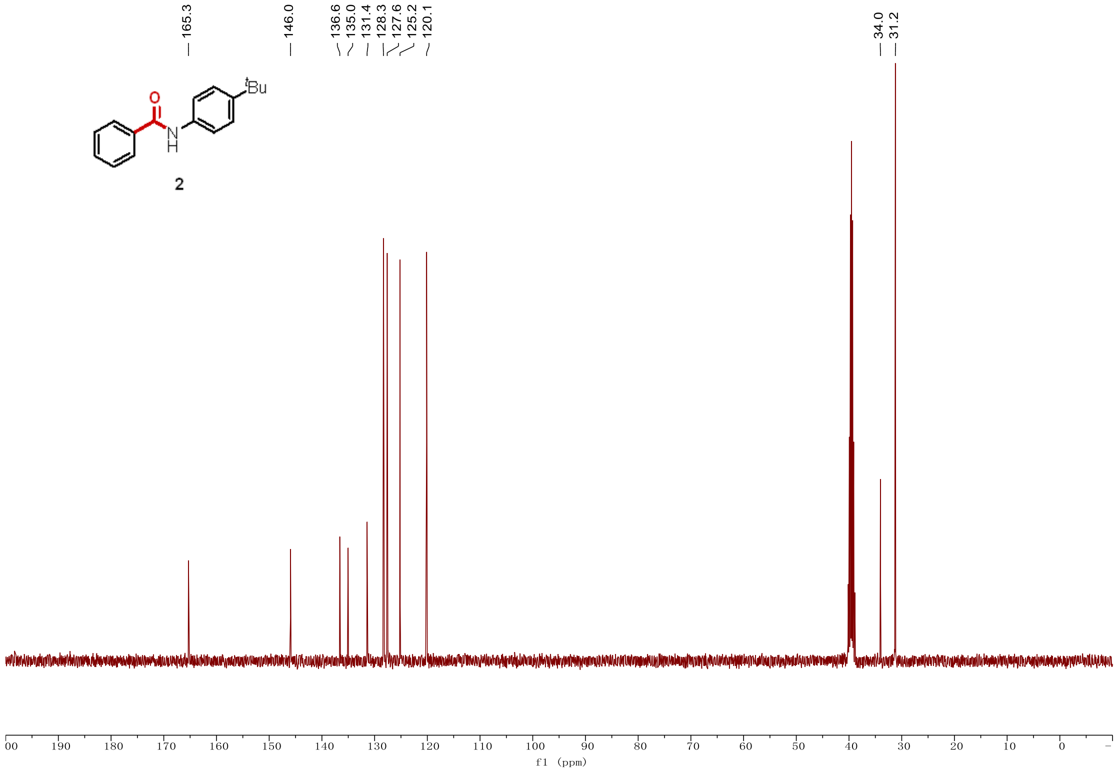

^{13}C NMR spectrum of **2** in DMSO-d_6 (101 MHz)

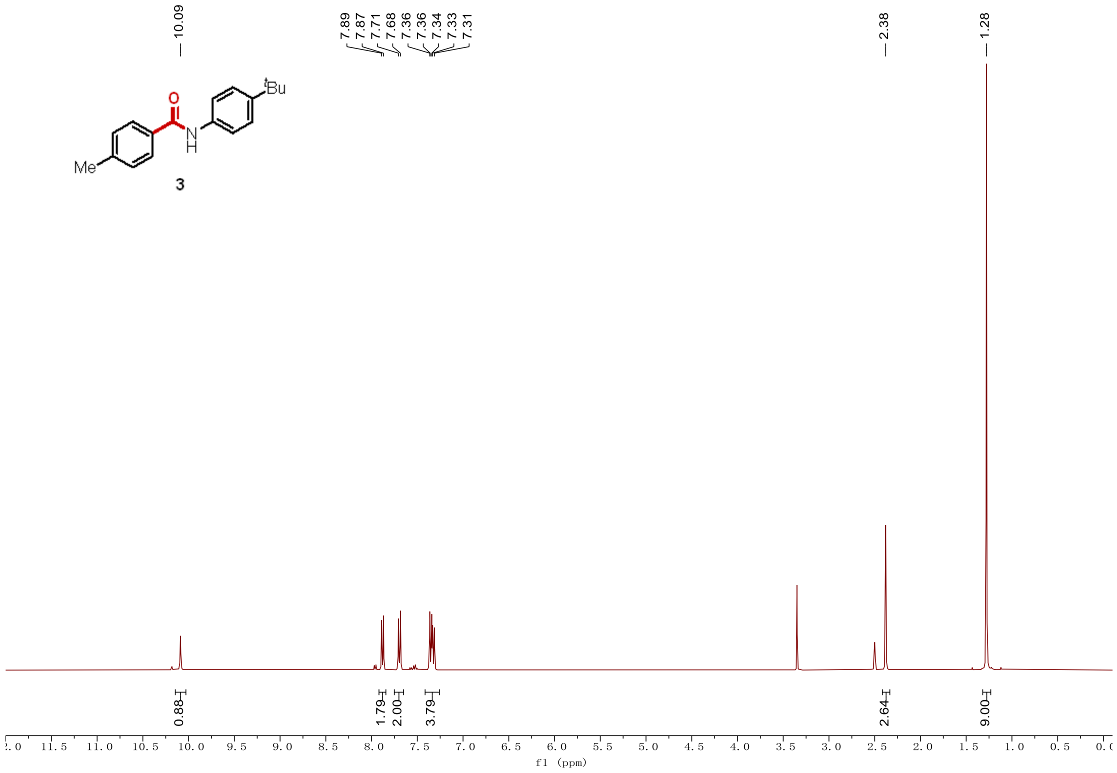

^{1}H NMR spectrum of **3** in DMSO-d_6 (400 MHz)

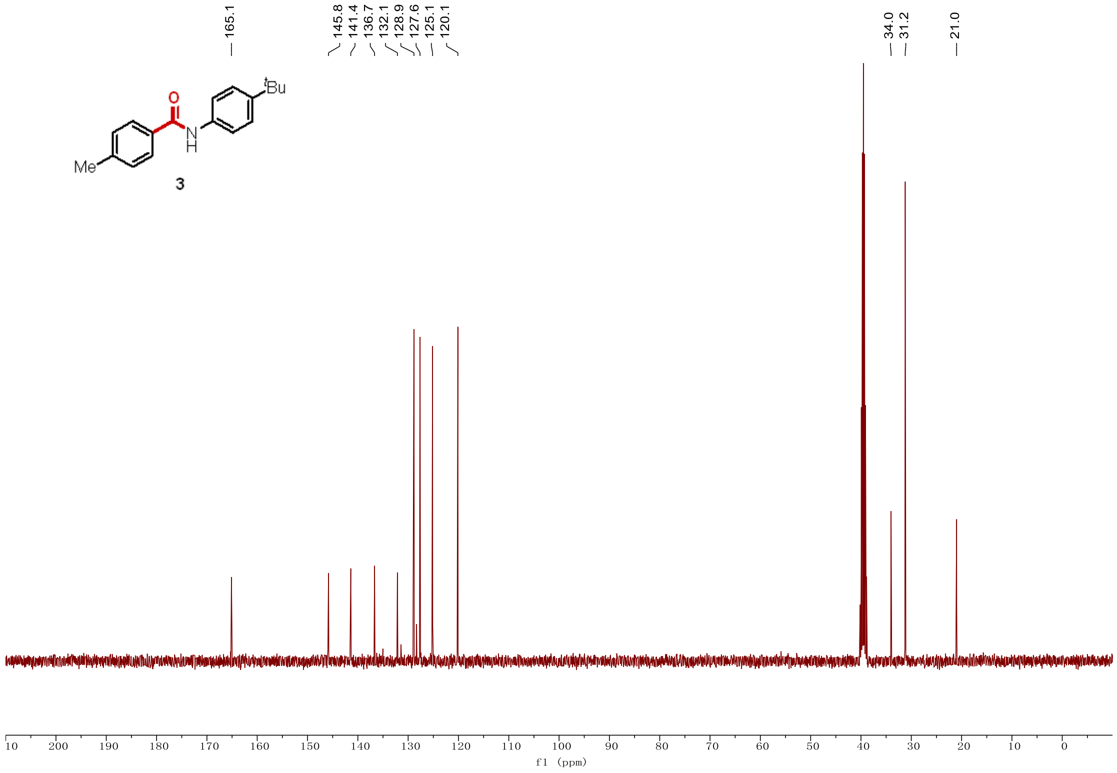

^{13}C NMR spectrum of **3** in DMSO-d_6 (101 MHz)

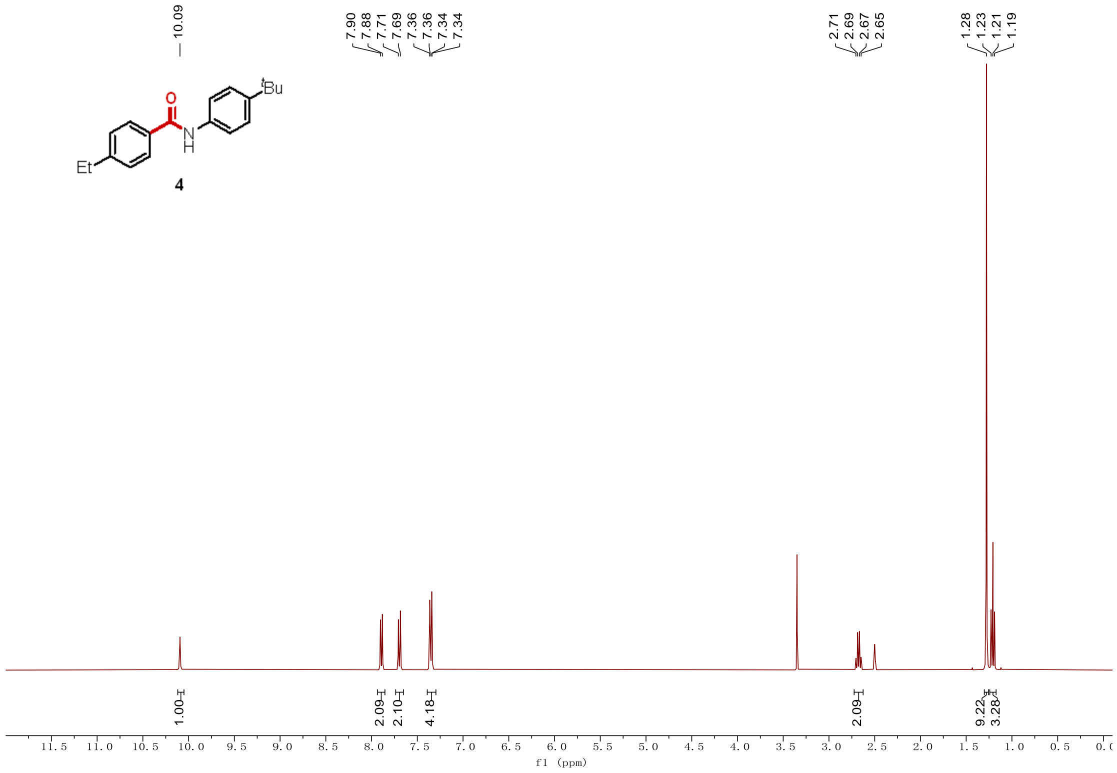

^{1}H NMR spectrum of **4** in DMSO-d_6 (400 MHz)

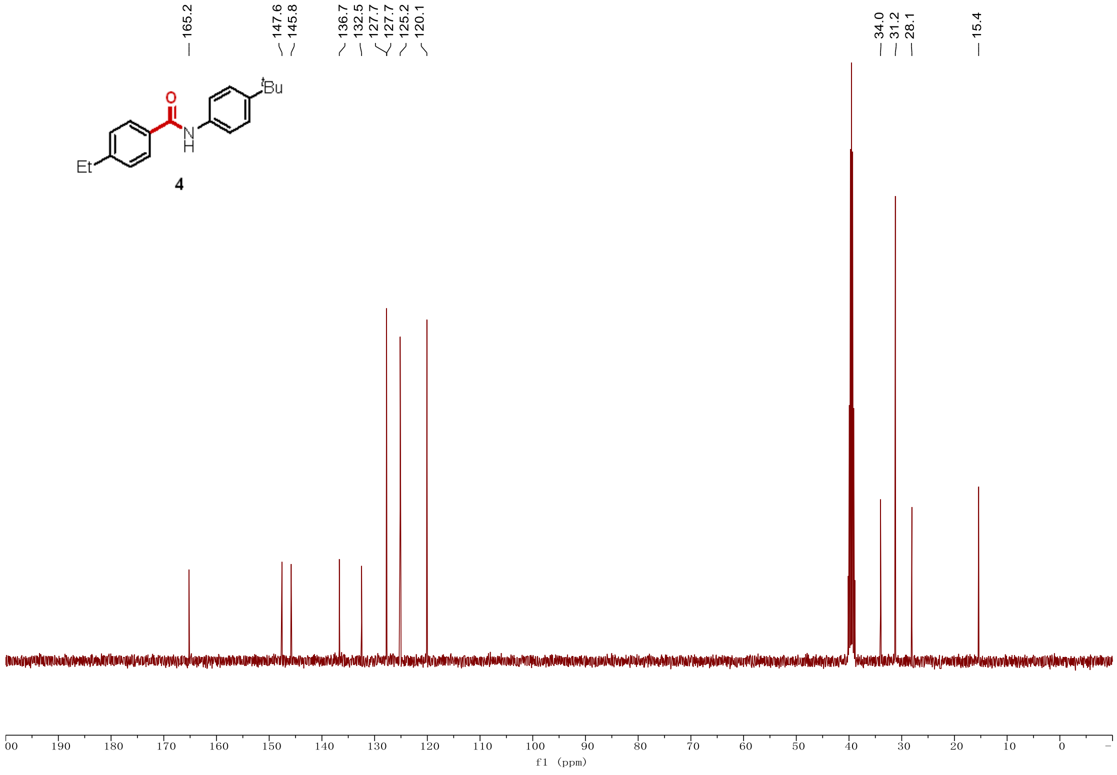

^{13}C NMR spectrum of **4** in DMSO-d_6 (101 MHz)

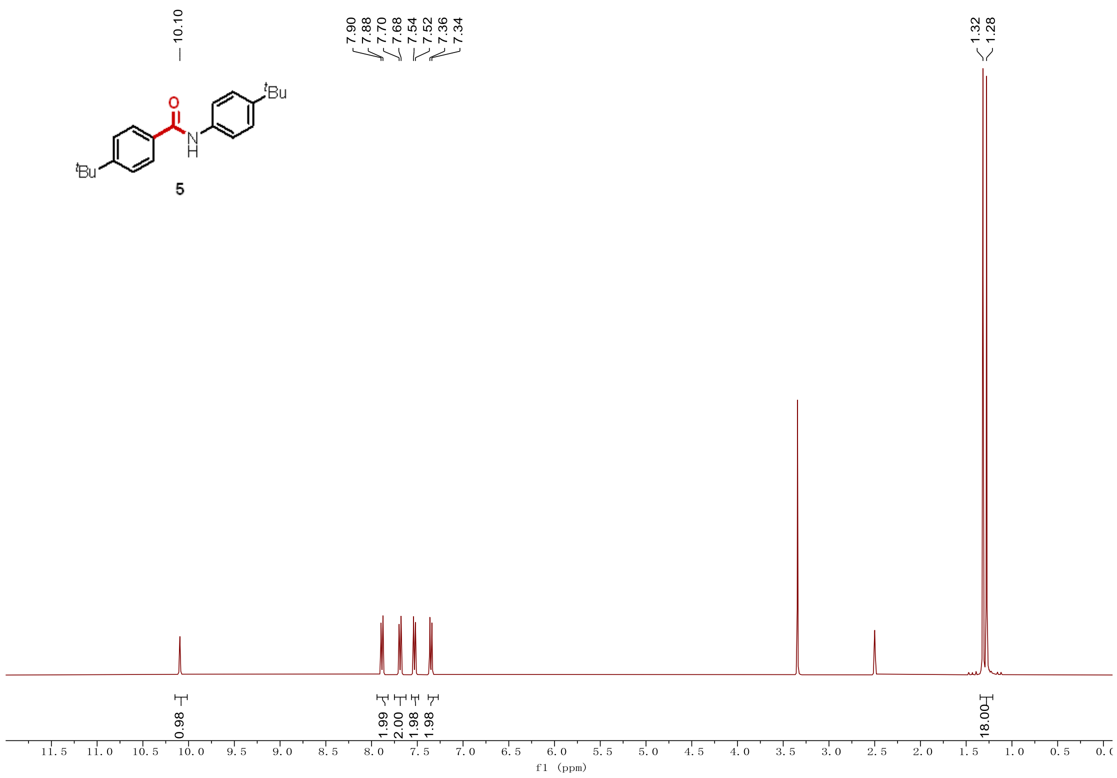

^{1}H NMR spectrum of **5** in DMSO-d_6 (400 MHz)

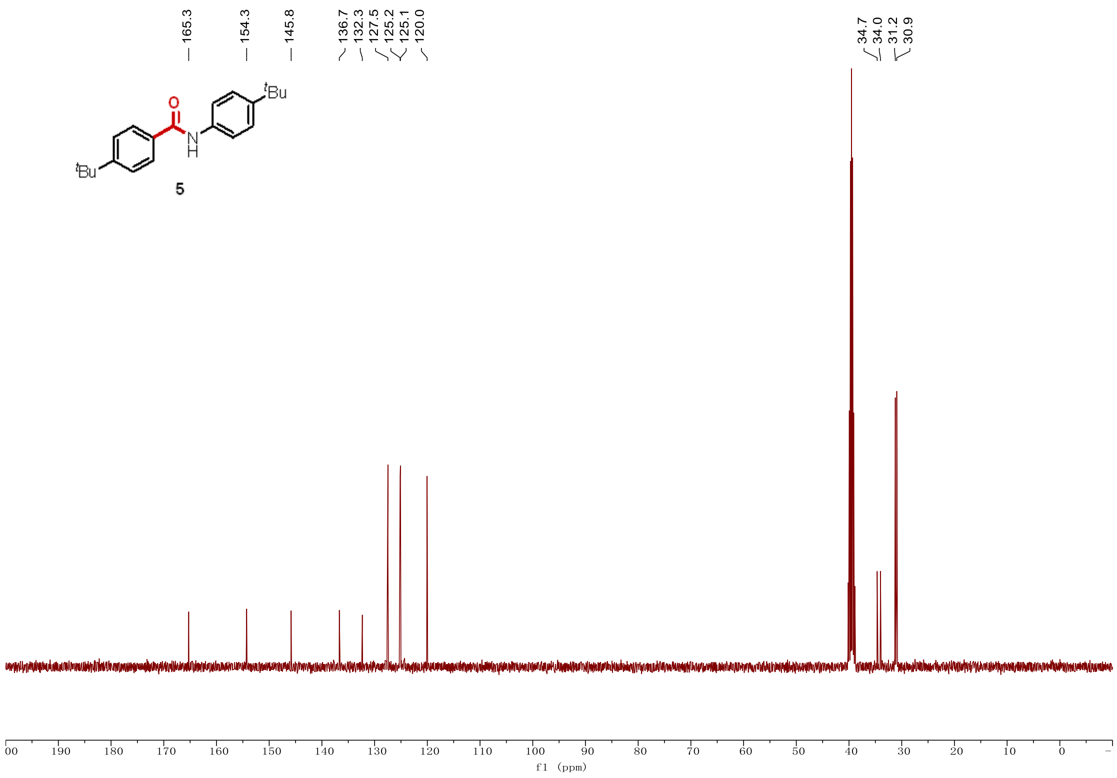

^{13}C NMR spectrum of **5** in DMSO-d_6 (101 MHz)

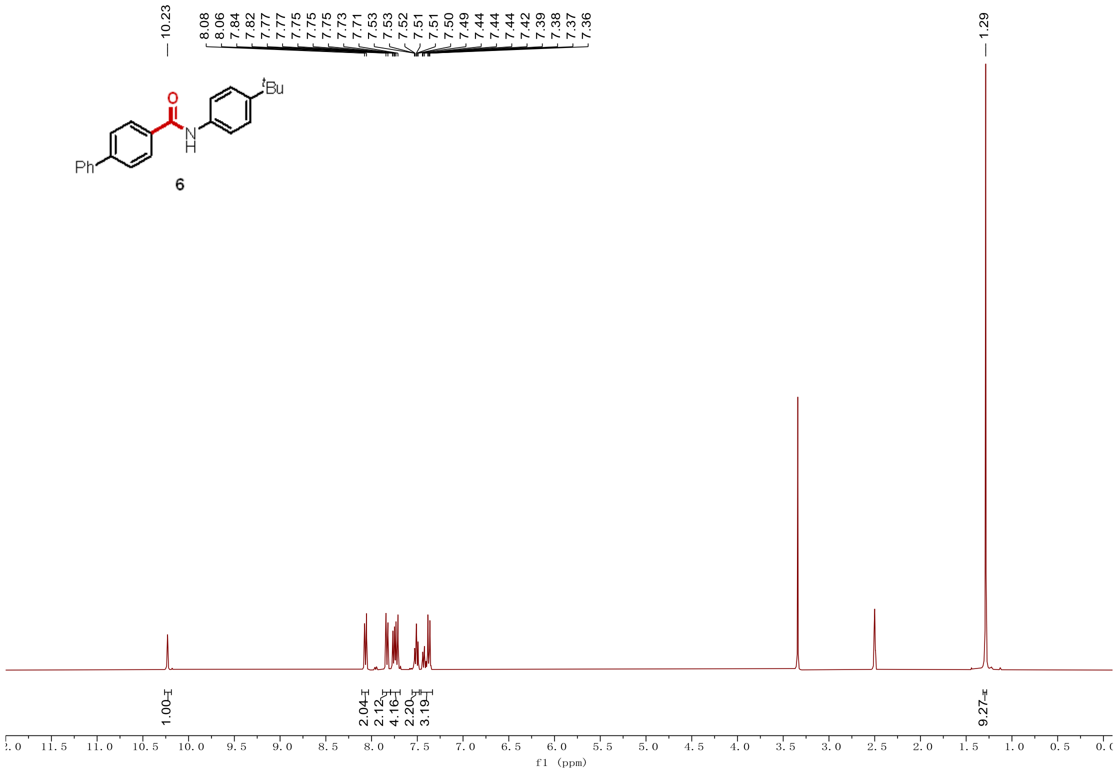

^{1}H NMR spectrum of **6** in DMSO-d_6 (400 MHz)

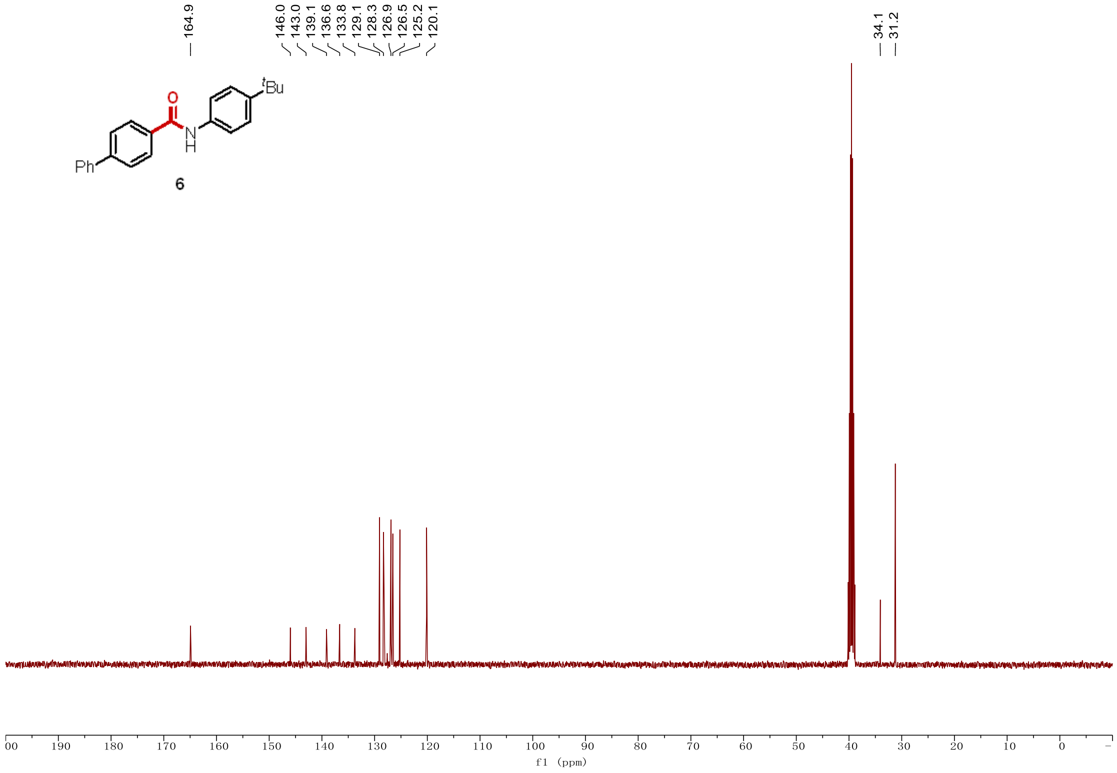

^{13}C NMR spectrum of **6** in DMSO-d_6 (101 MHz)

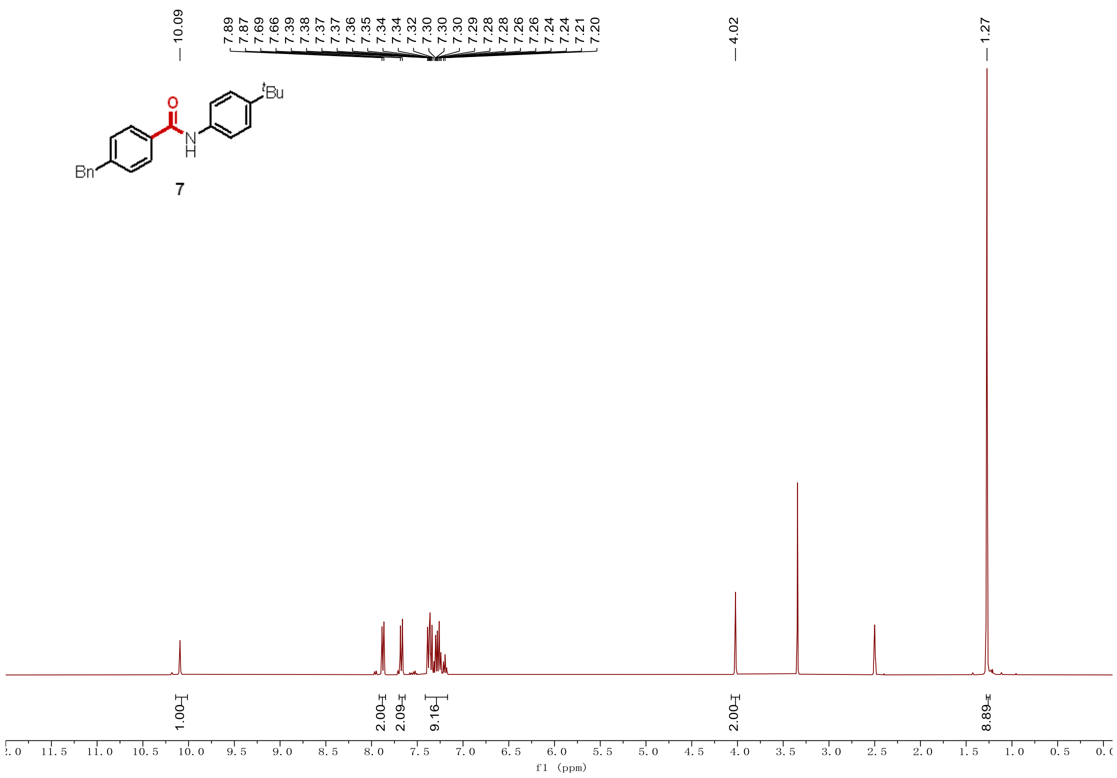

^{1}H NMR spectrum of **7** in DMSO-d_6 (400 MHz)

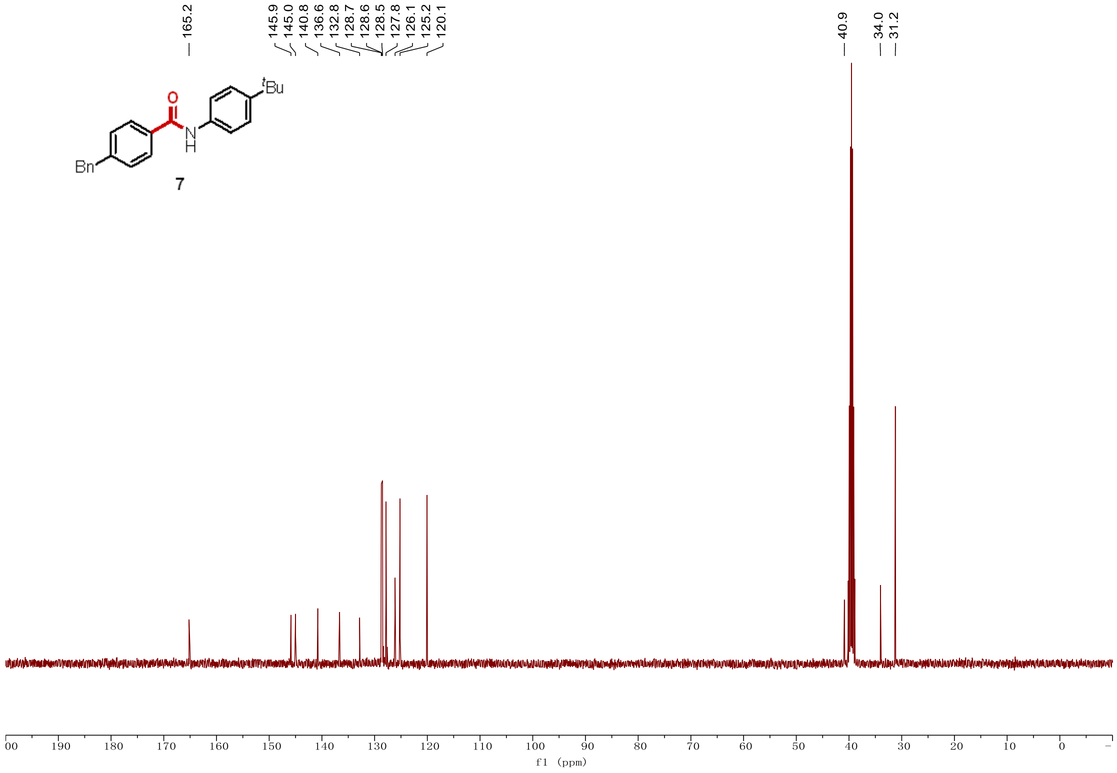

^{13}C NMR spectrum of **7** in DMSO-d_6 (101 MHz)

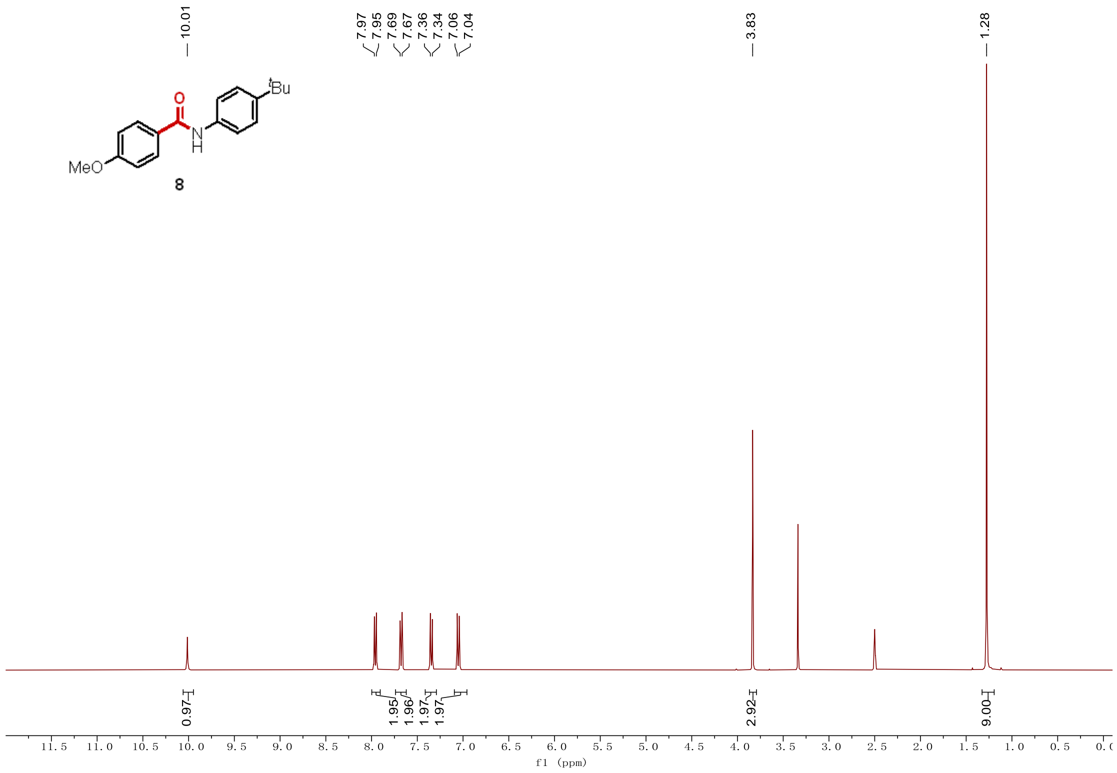

^{1}H NMR spectrum of **8** in DMSO-d_6 (400 MHz)

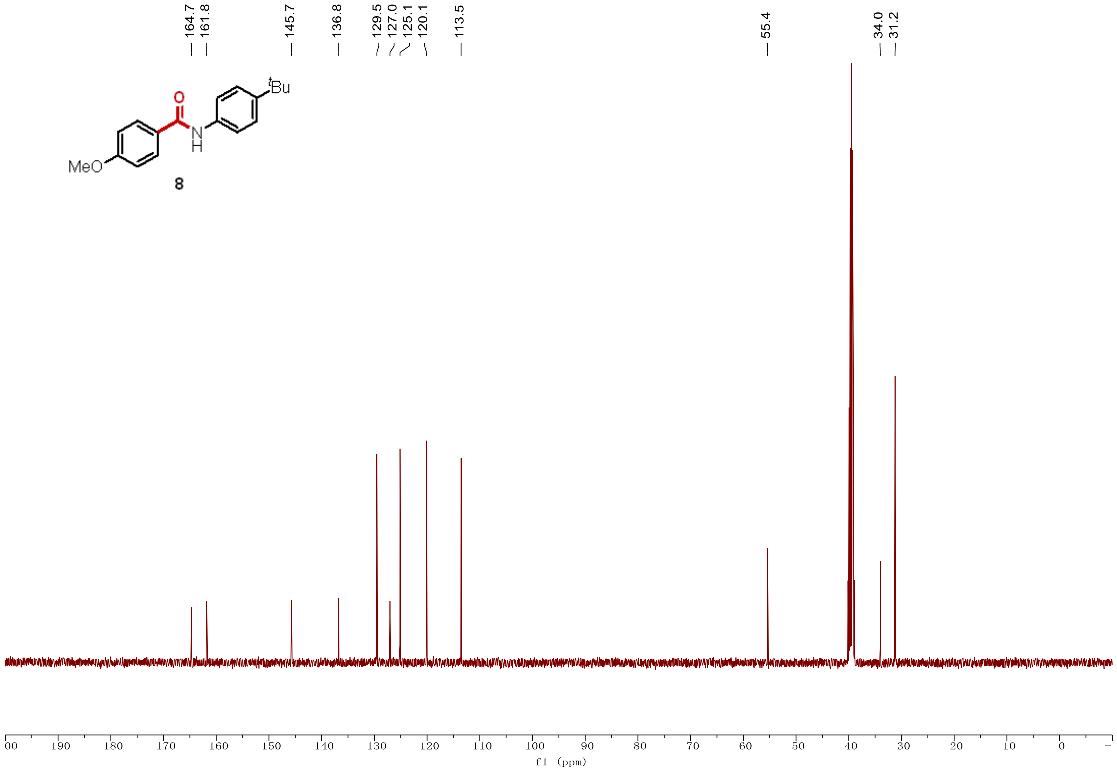

^{13}C NMR spectrum of **8** in DMSO-d_6 (101 MHz)

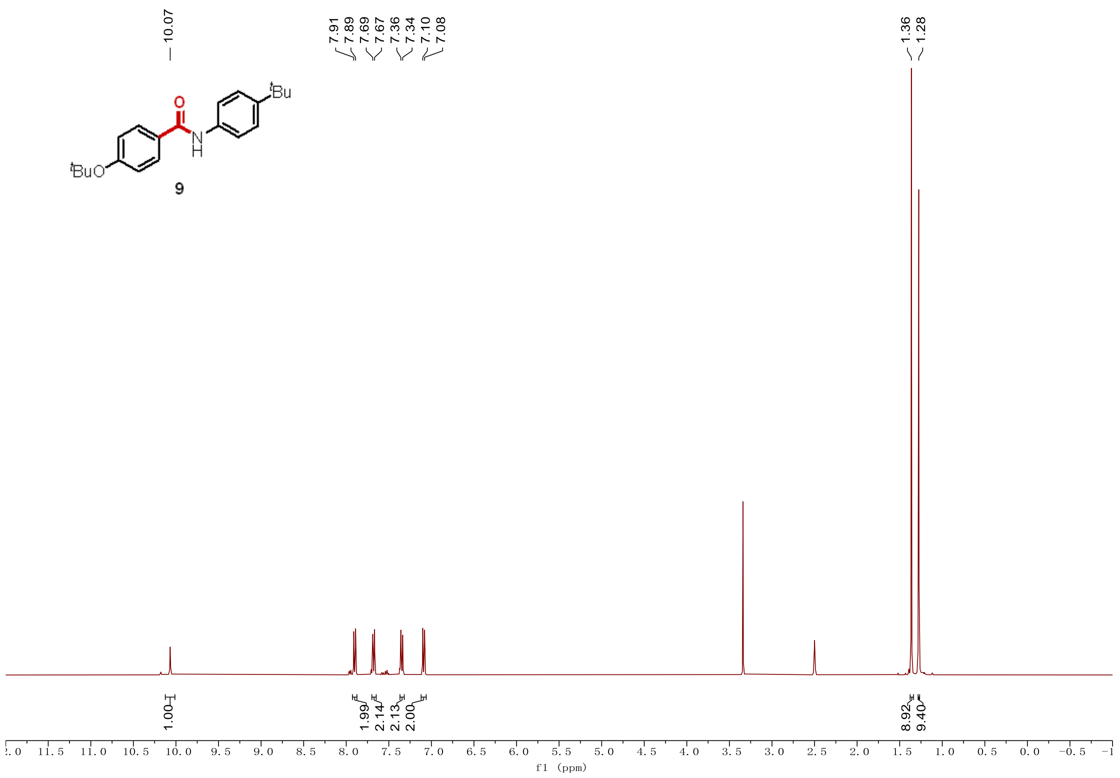

^{1}H NMR spectrum of **9** in DMSO-d_6 (400 MHz)

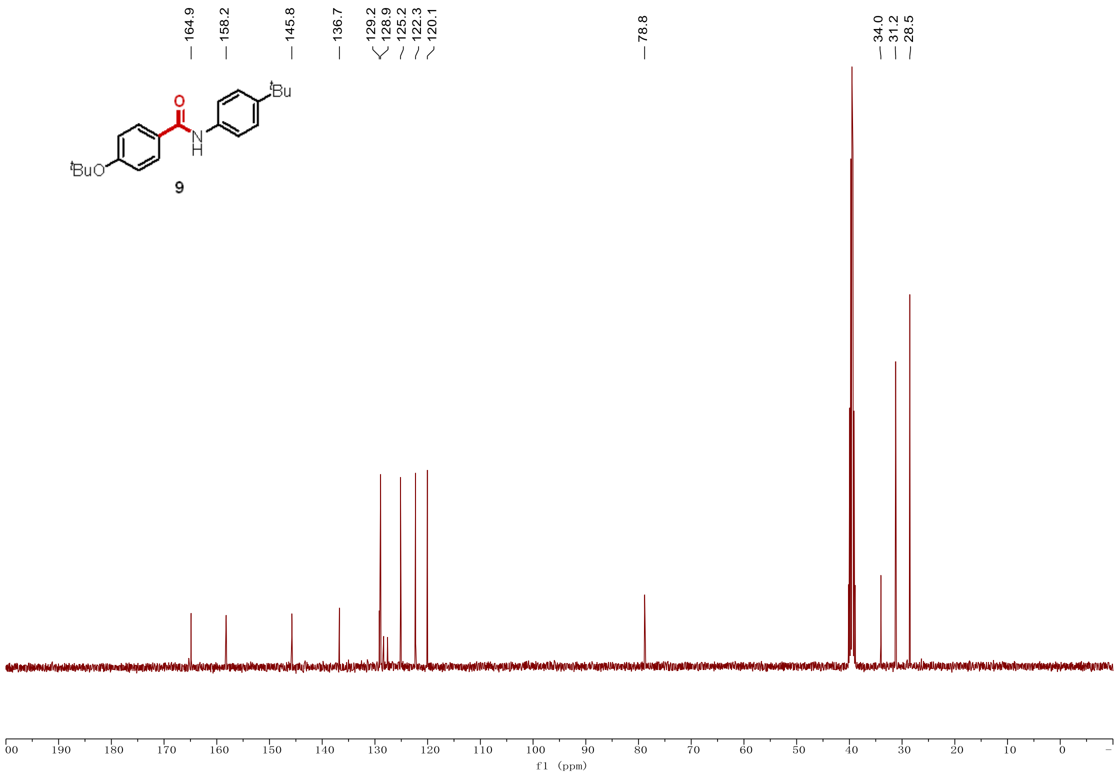

^{13}C NMR spectrum of **9** in DMSO-d_6 (101 MHz)

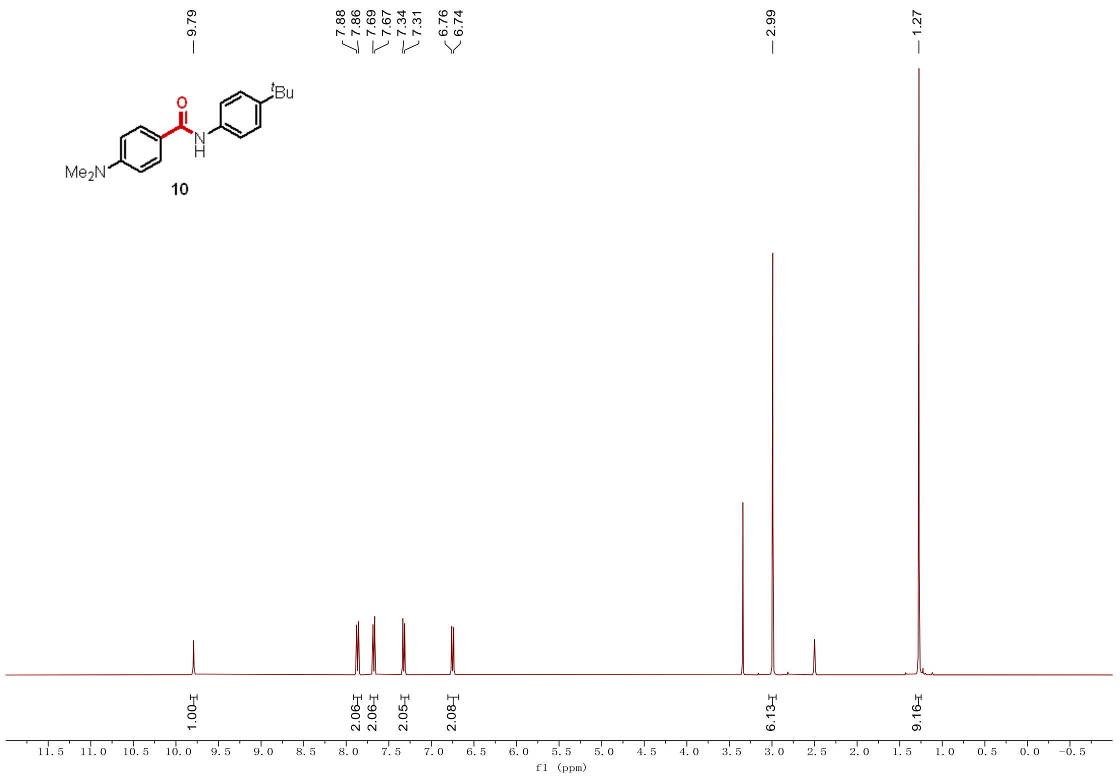

^{1}H NMR spectrum of **10** in DMSO-d_6 (400 MHz)

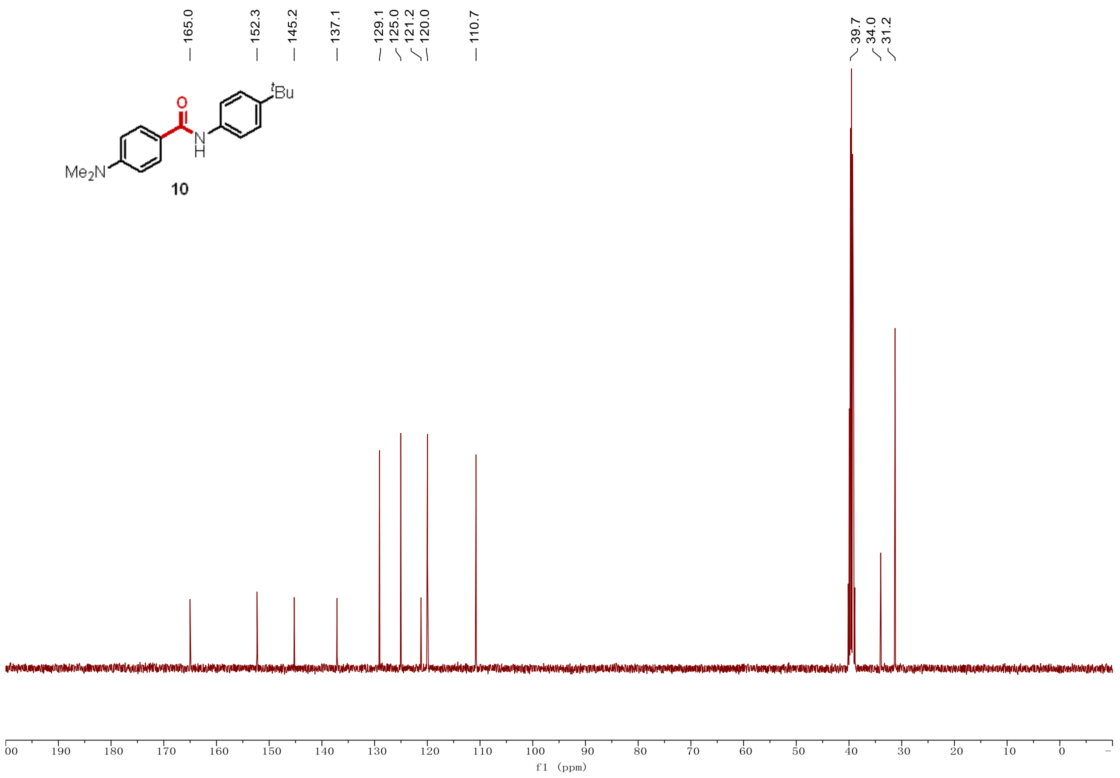

^{13}C NMR spectrum of **10** in DMSO-d_6 (101 MHz)

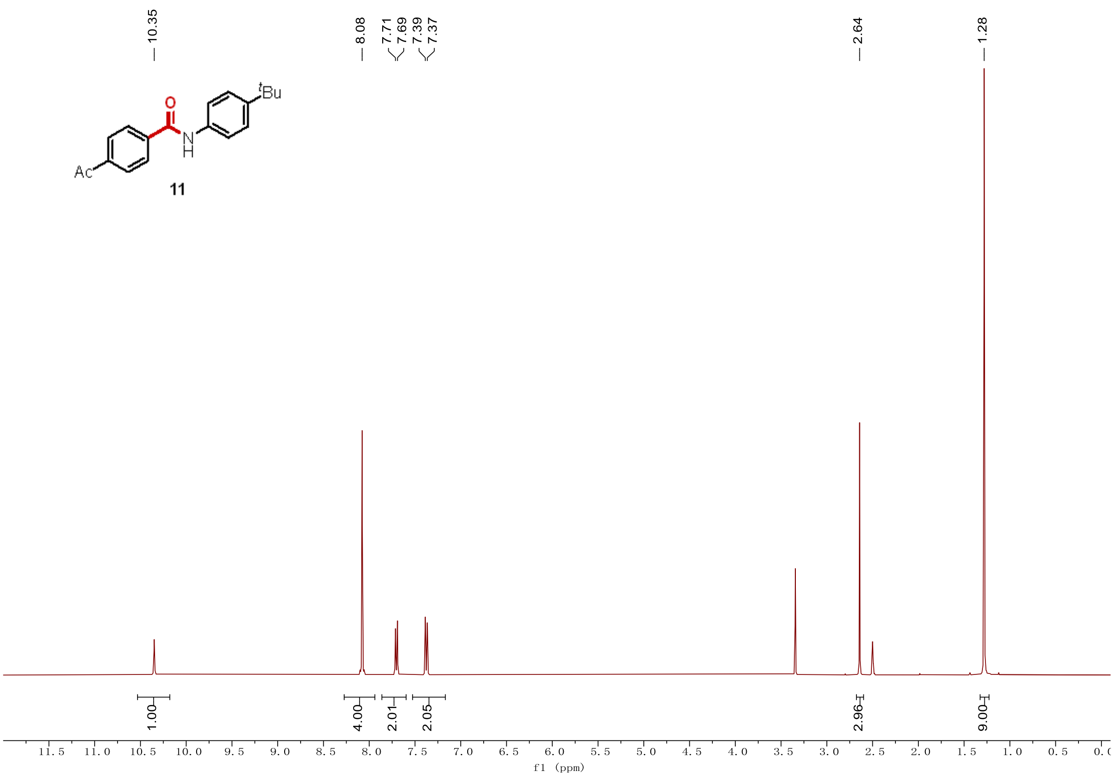

^{1}H NMR spectrum of **11** in DMSO-d_6 (400 MHz)

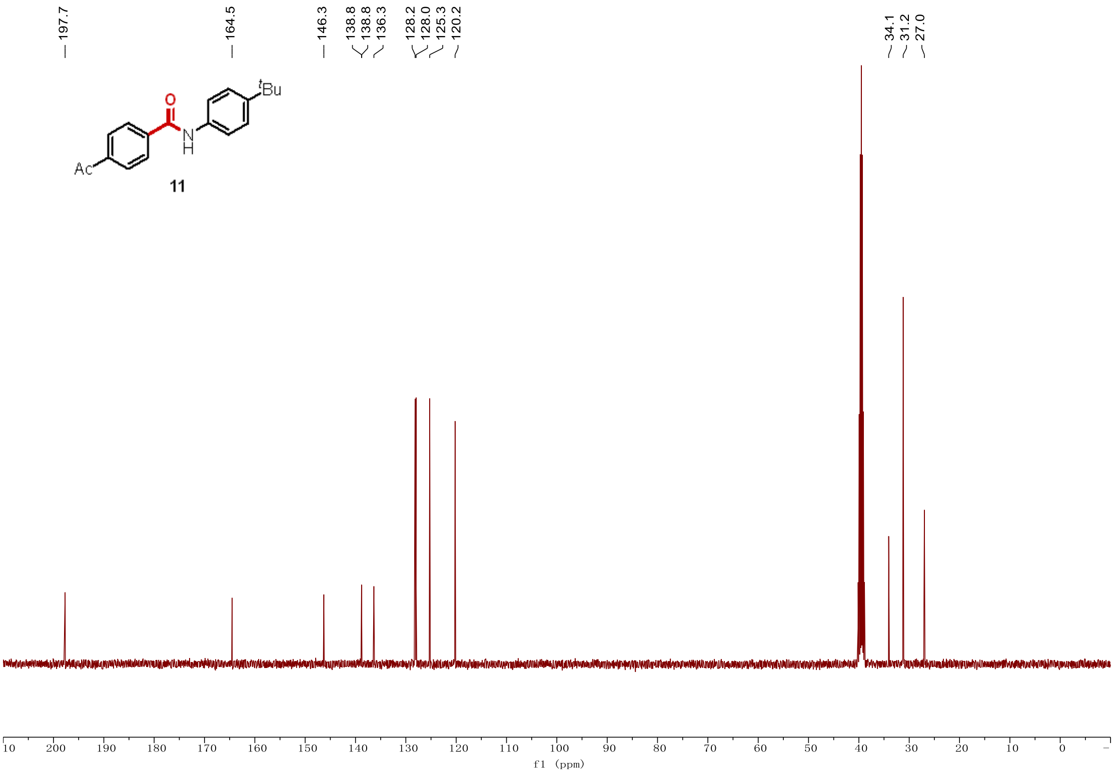

^{13}C NMR spectrum of **11** in DMSO-d_6 (101 MHz)

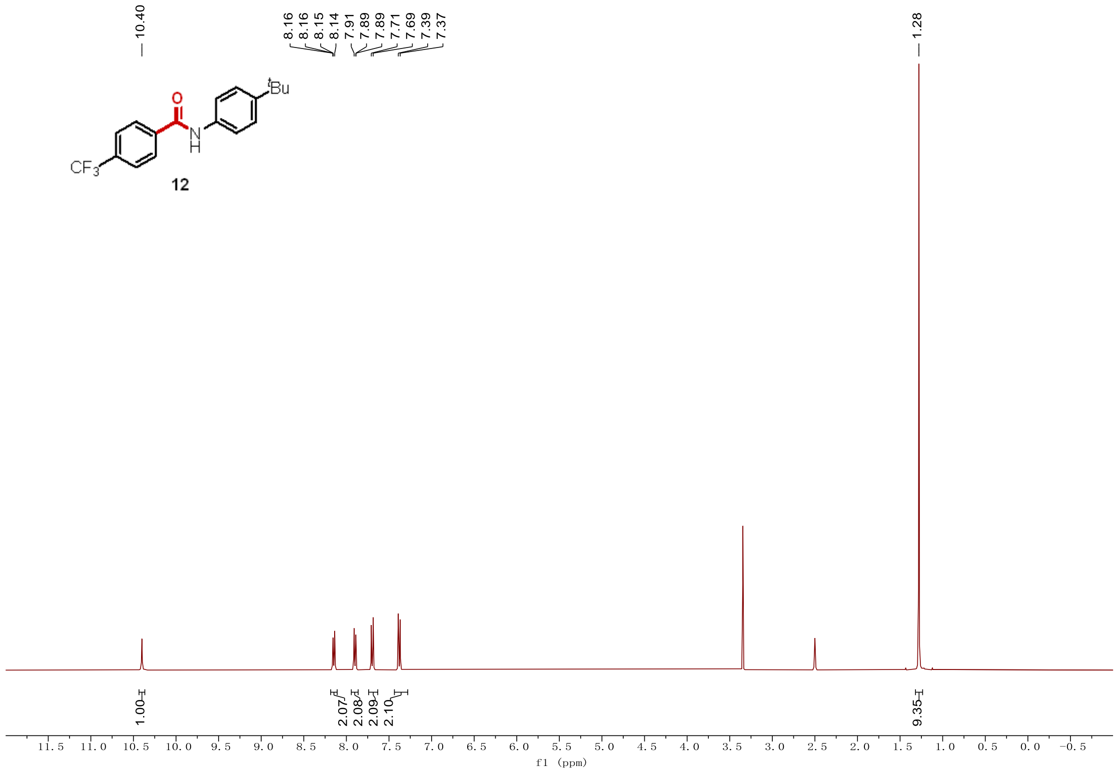

^{13}C NMR spectrum of **12** in DMSO-d_6 (101 MHz)

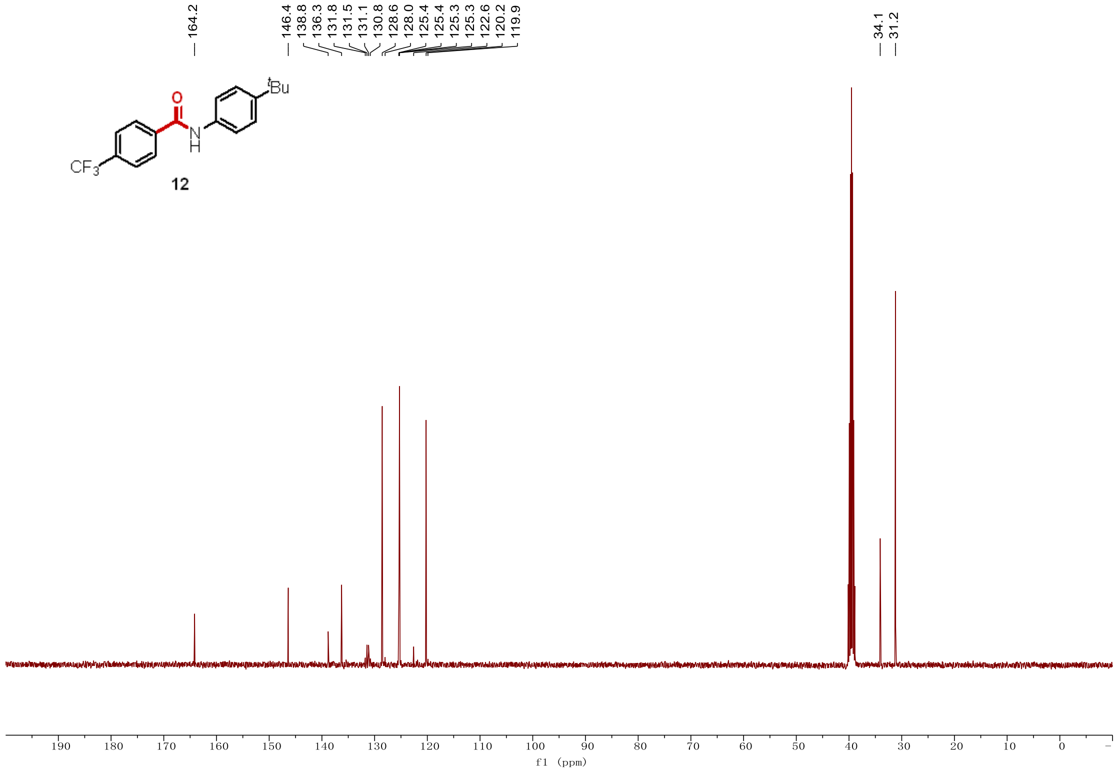

^{13}C NMR spectrum of **12** in DMSO-d_6 (101 MHz)

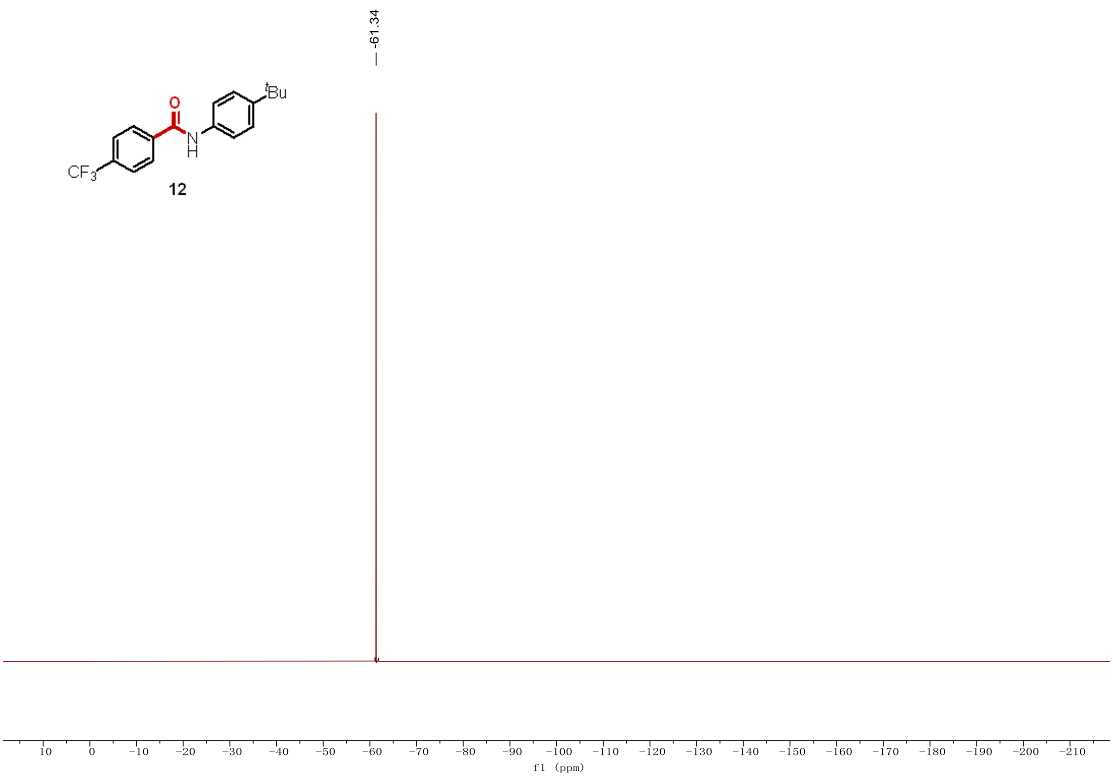

^{19}F NMR spectrum of **12** in DMSO-d_6 (376 MHz)

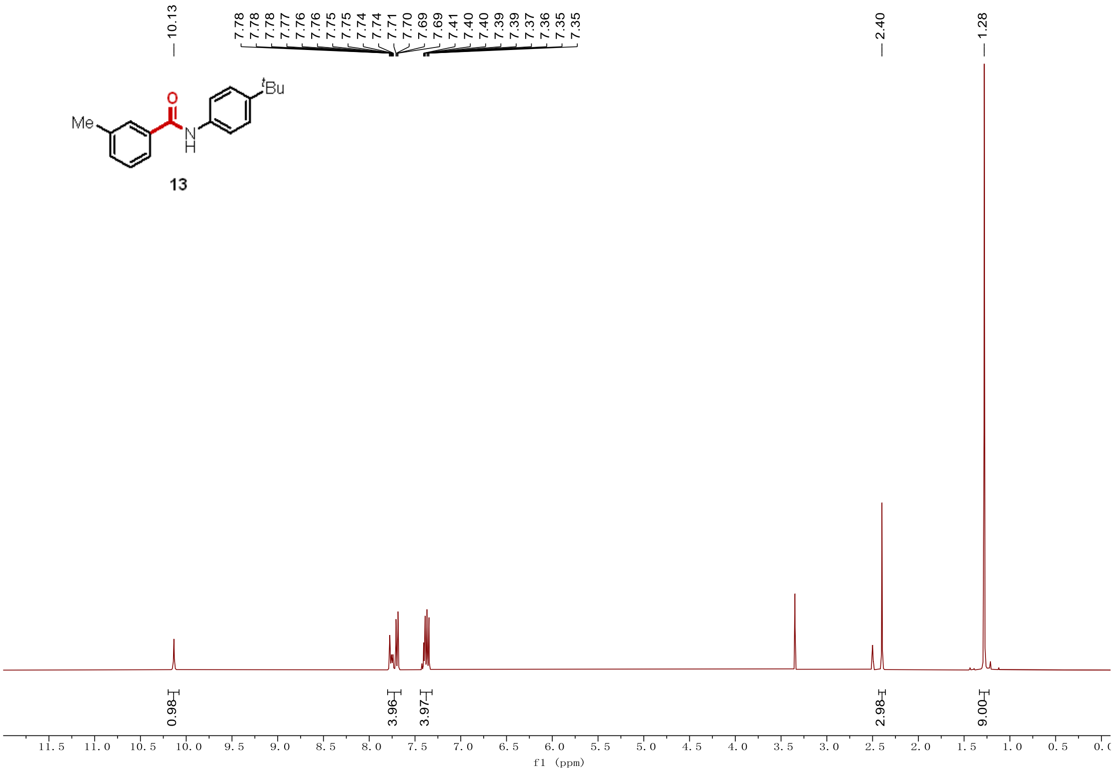

^{1}H NMR spectrum of **13** in DMSO-d_6 (400 MHz)

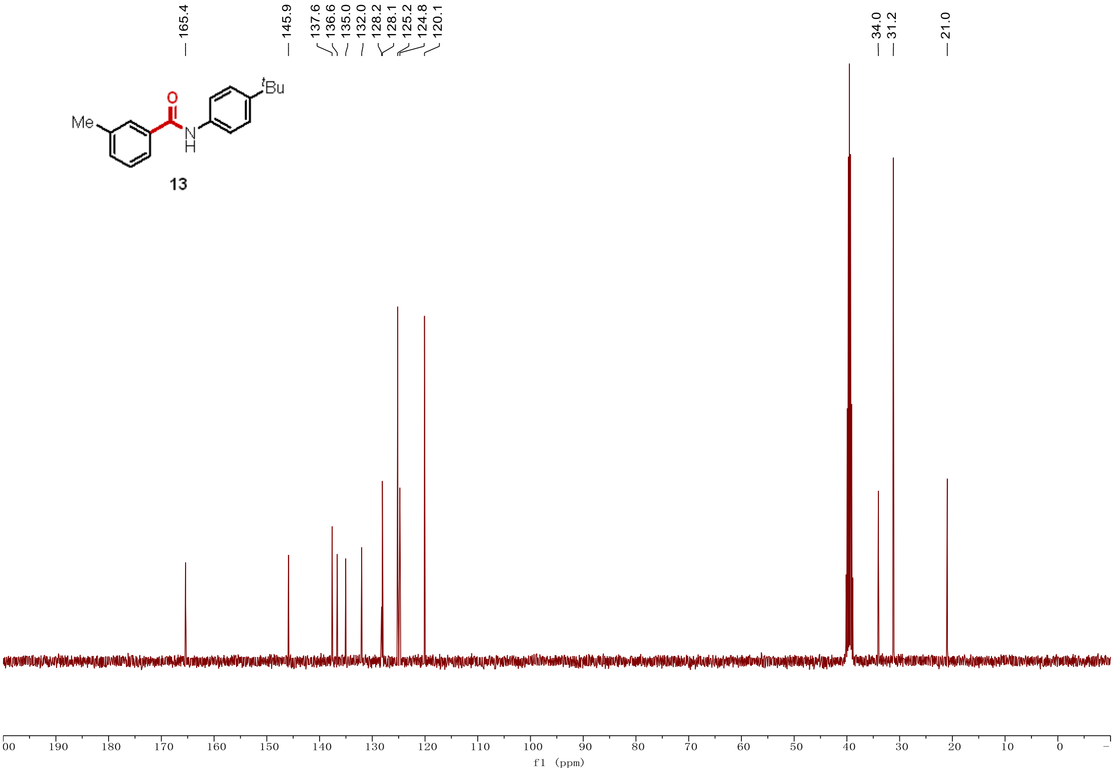

^{13}C NMR spectrum of **13** in DMSO-d_6 (101 MHz)

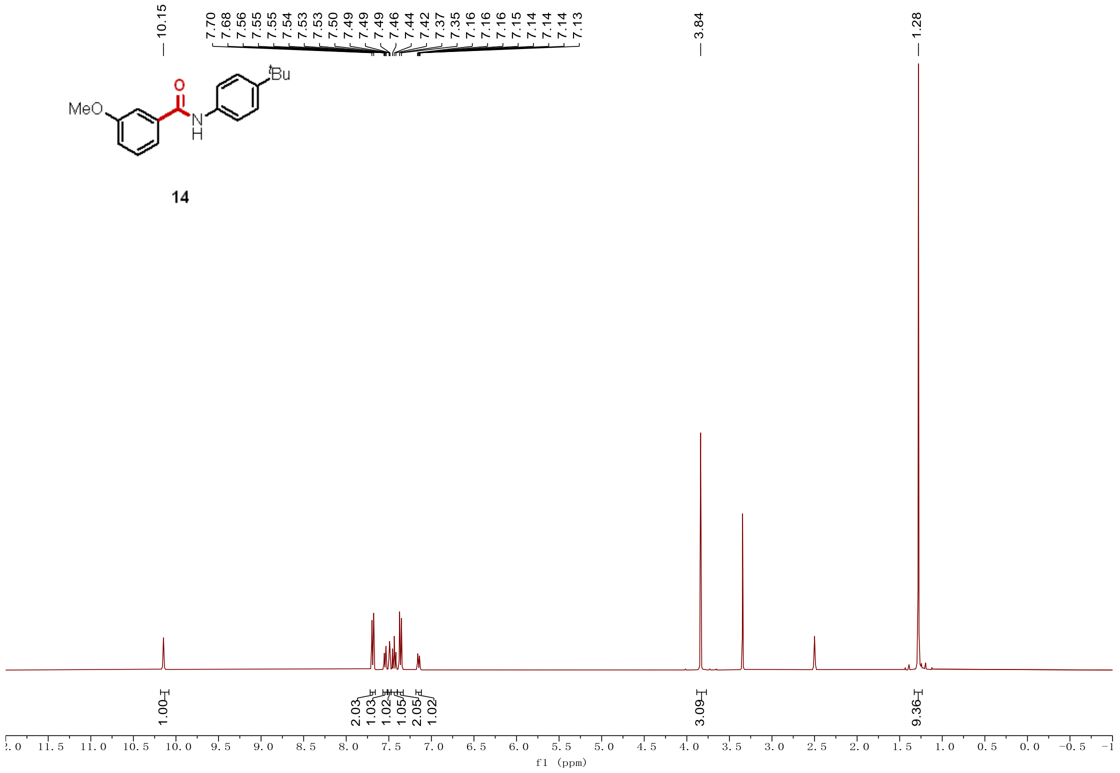

^{1}H NMR spectrum of **14** in DMSO-d_6 (400 MHz)

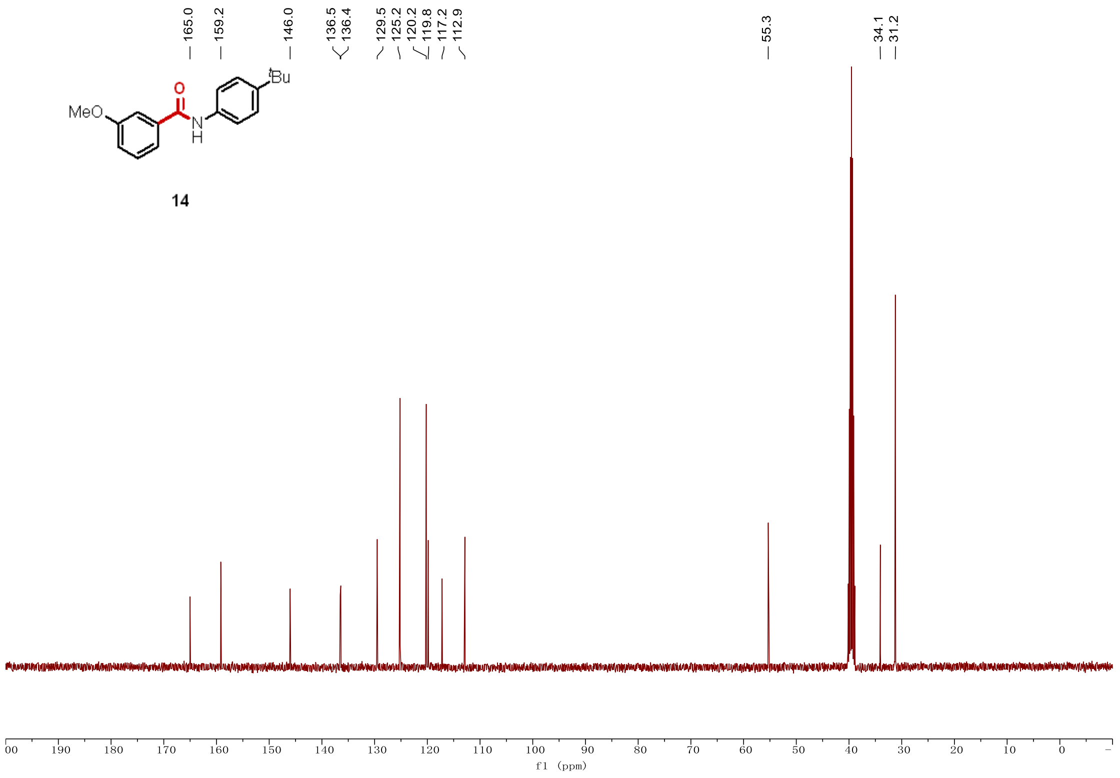

^{13}C NMR spectrum of **14** in DMSO-d_6 (101 MHz)

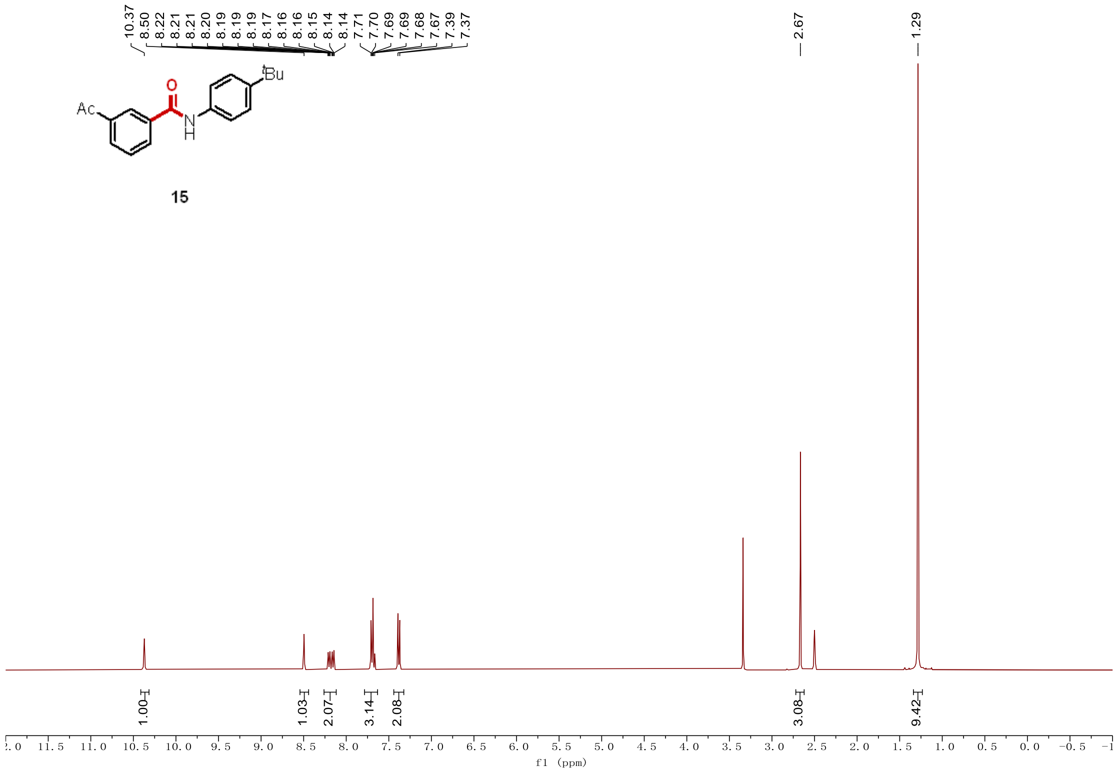

^{1}H NMR spectrum of **15** in DMSO-d_6 (400 MHz)

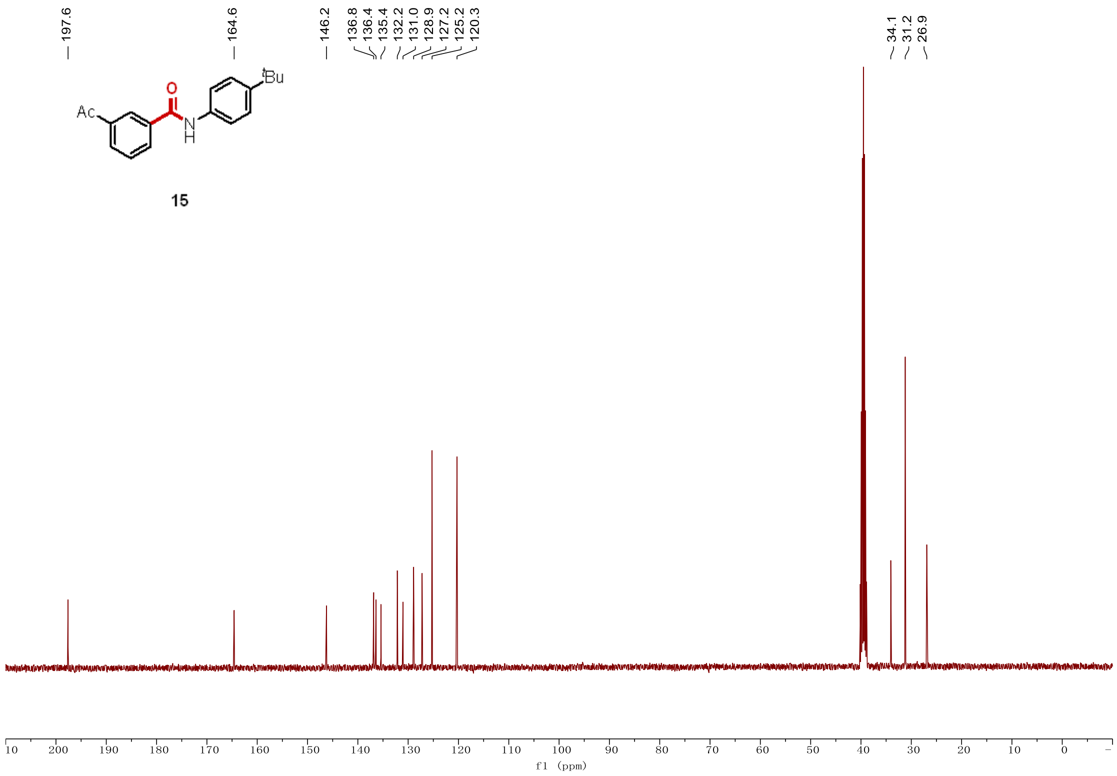

^{13}C NMR spectrum of **15** in DMSO-d_6 (101 MHz)

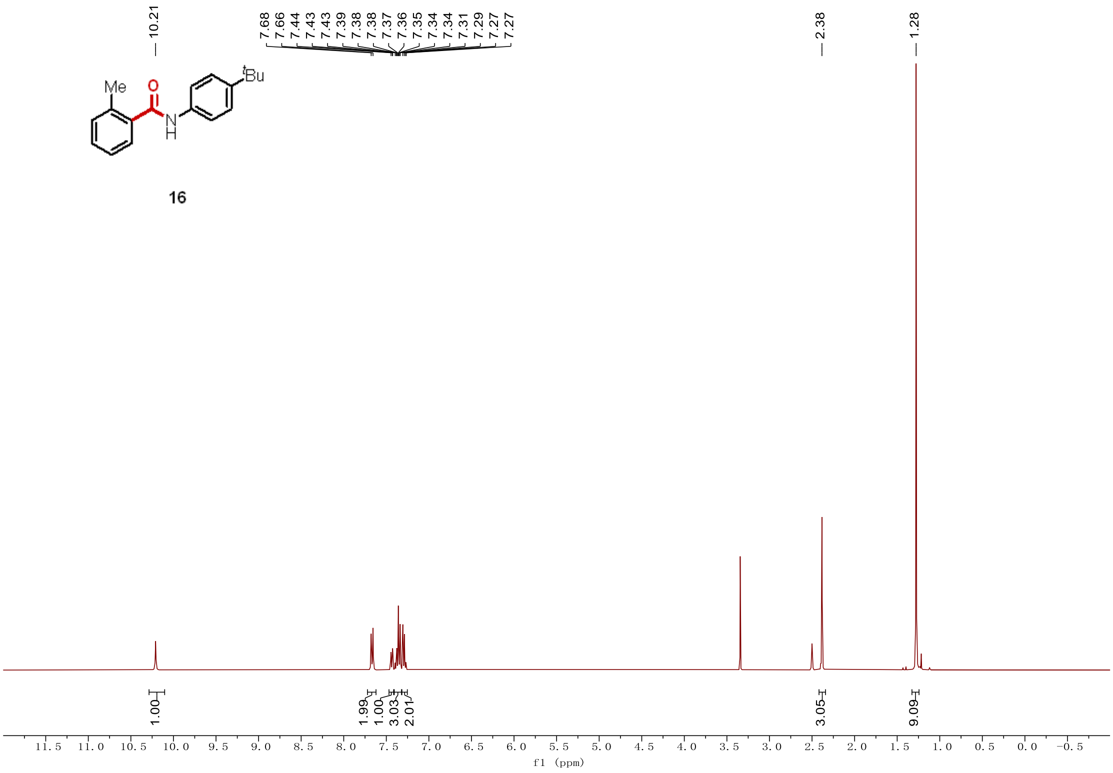

^{1}H NMR spectrum of **16** in DMSO-d_6 (400 MHz)

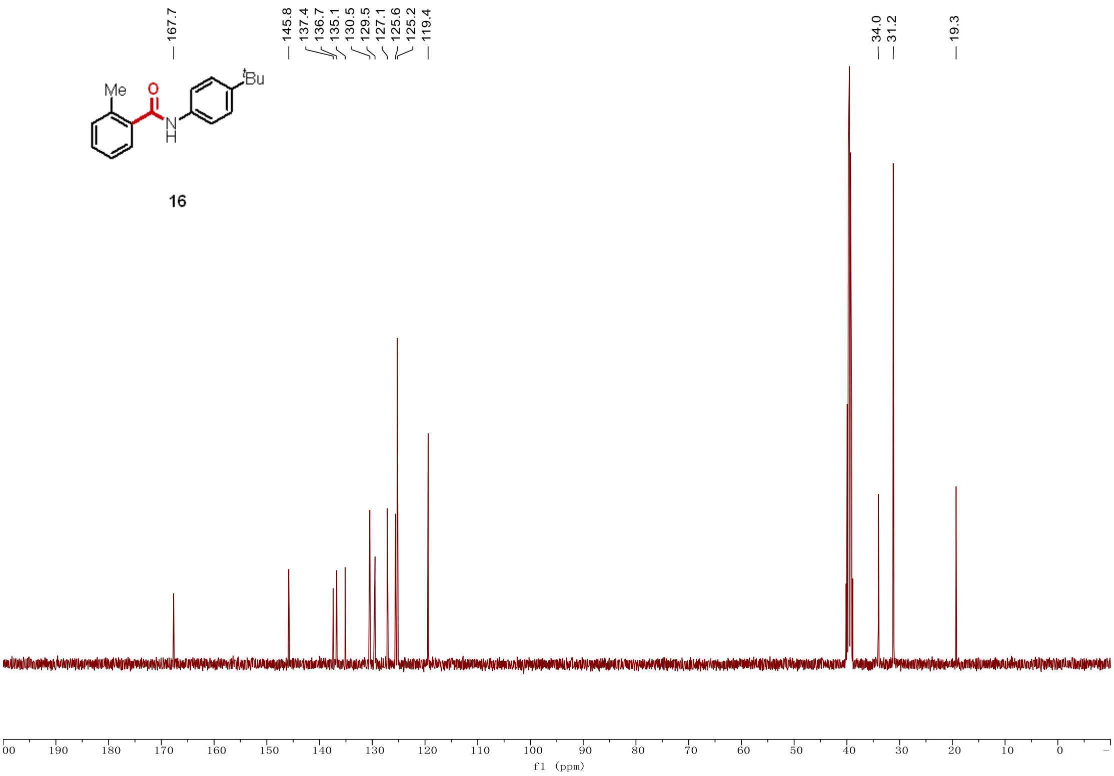

^{13}C NMR spectrum of **16** in DMSO-d_6 (101 MHz)

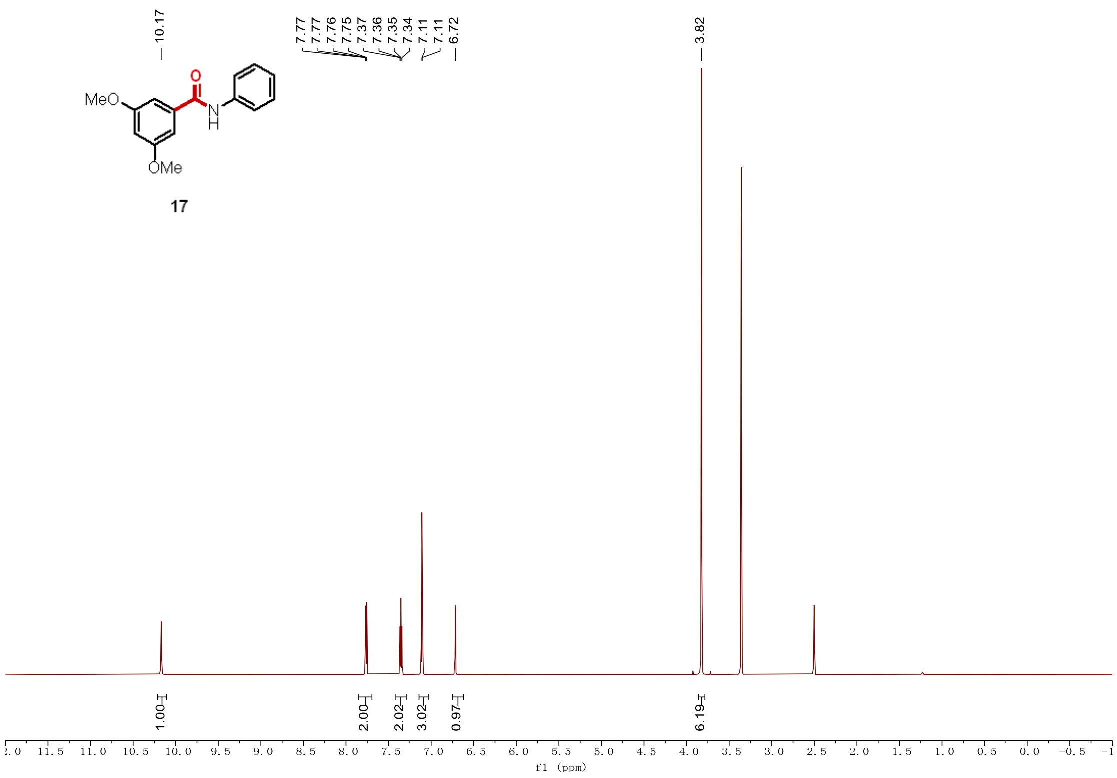

^{1}H NMR spectrum of **17** in DMSO-d_6 (400 MHz)

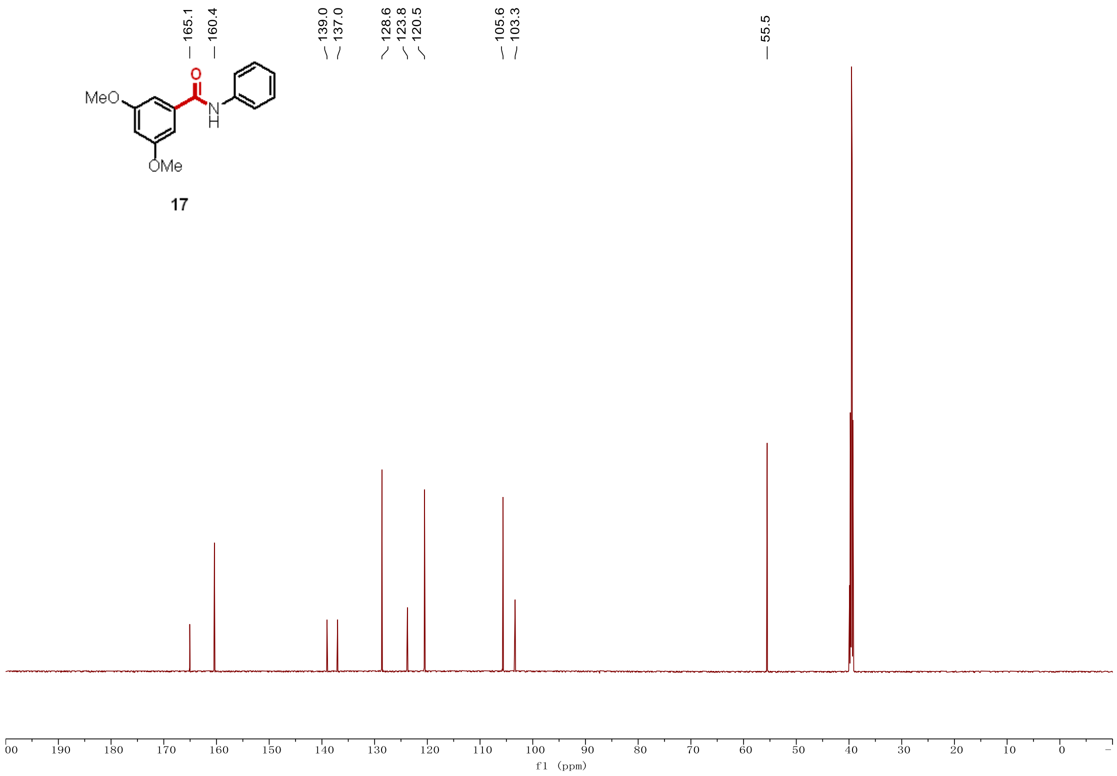

^{13}C NMR spectrum of **17** in DMSO-d_6 (101 MHz)

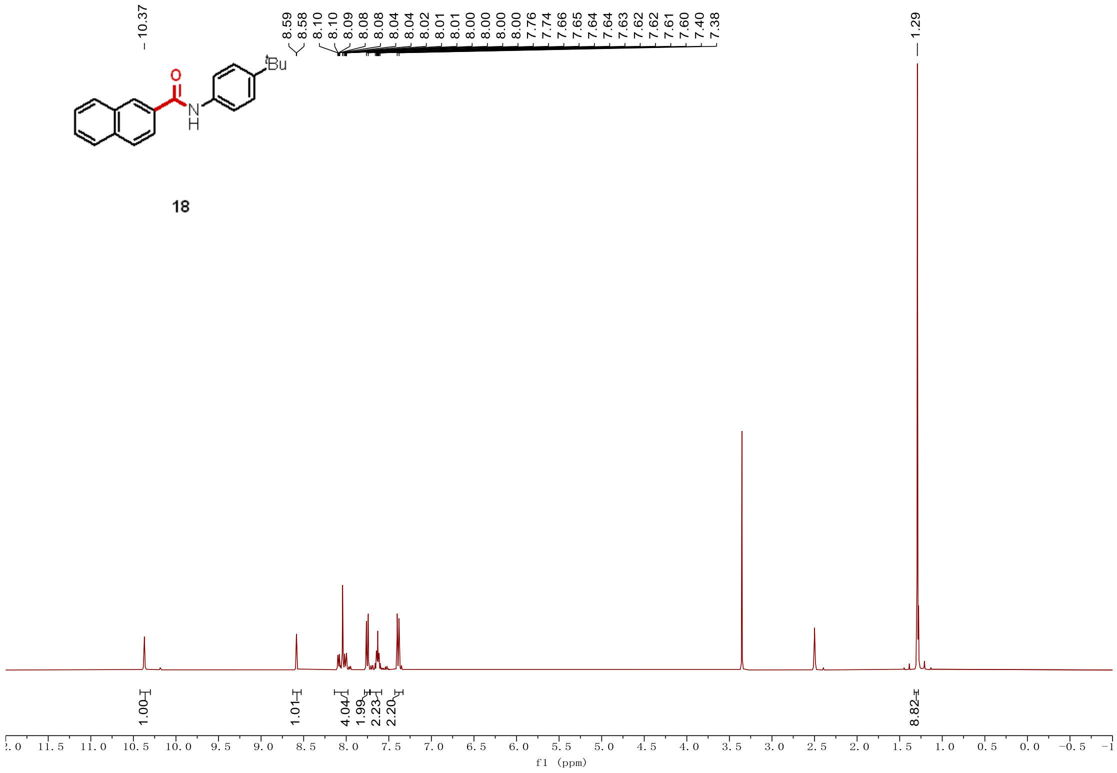

^{1}H NMR spectrum of **18** in DMSO-d_6 (400 MHz)

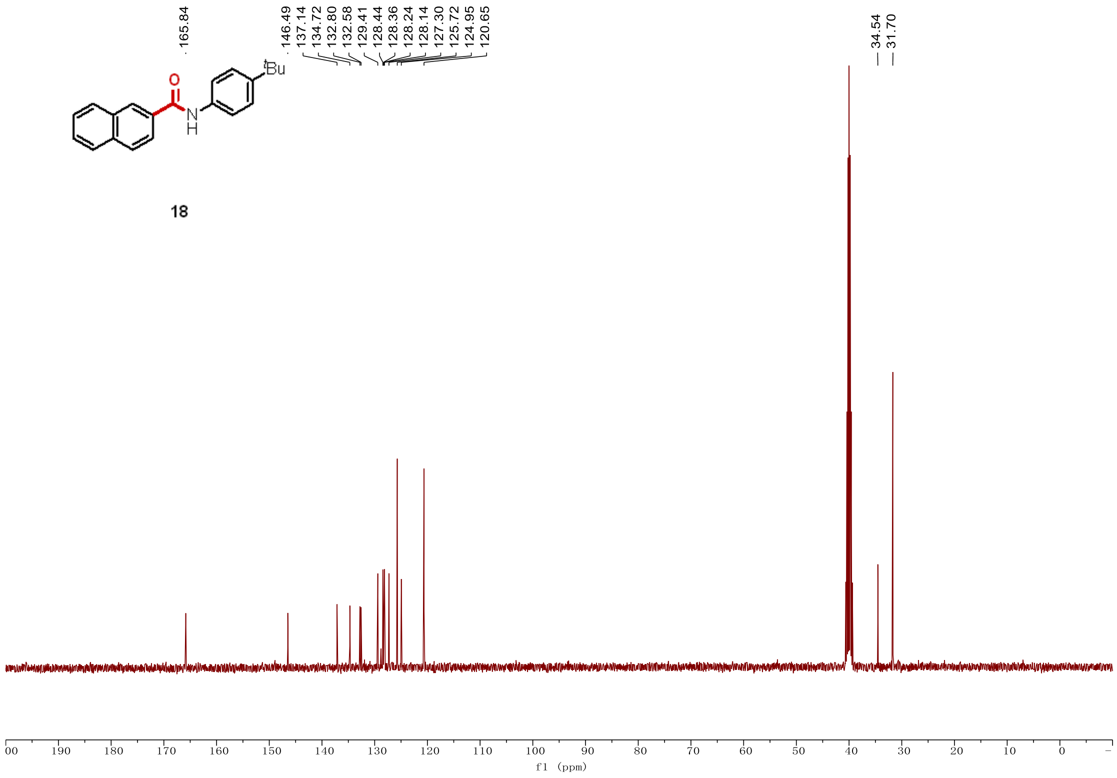

^{13}C NMR spectrum of **18** in DMSO-d_6 (101 MHz)

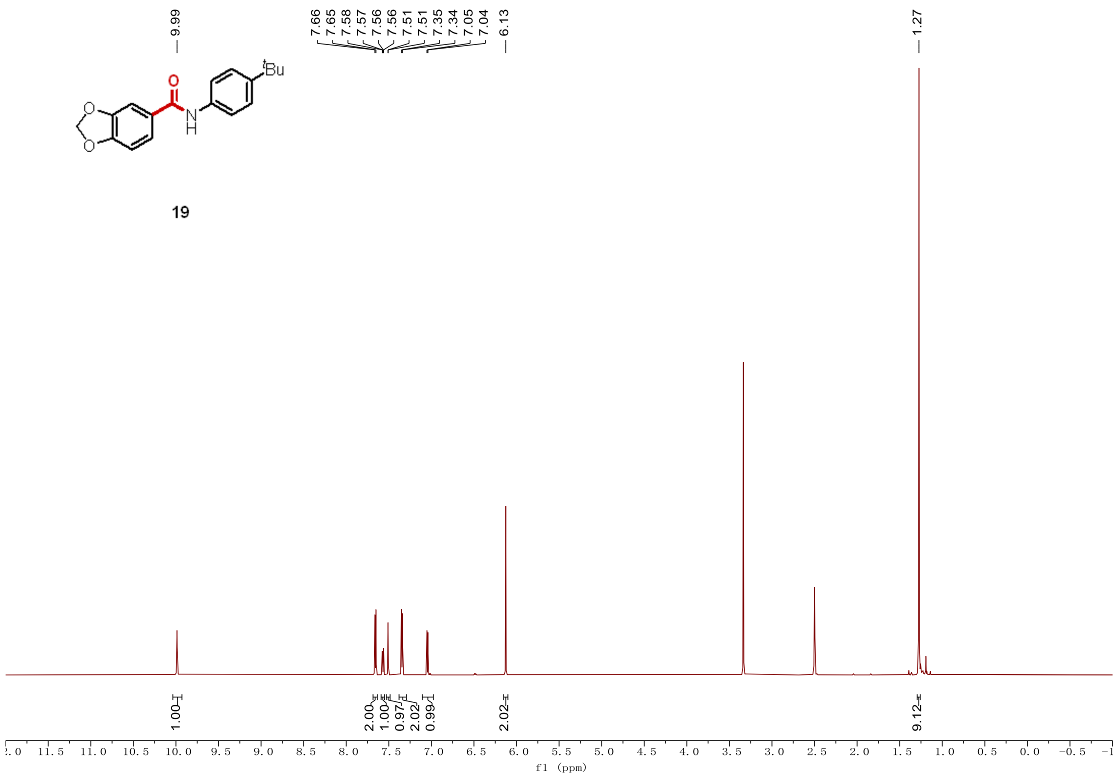

^{1}H NMR spectrum of **19** in DMSO-d_6 (700 MHz)

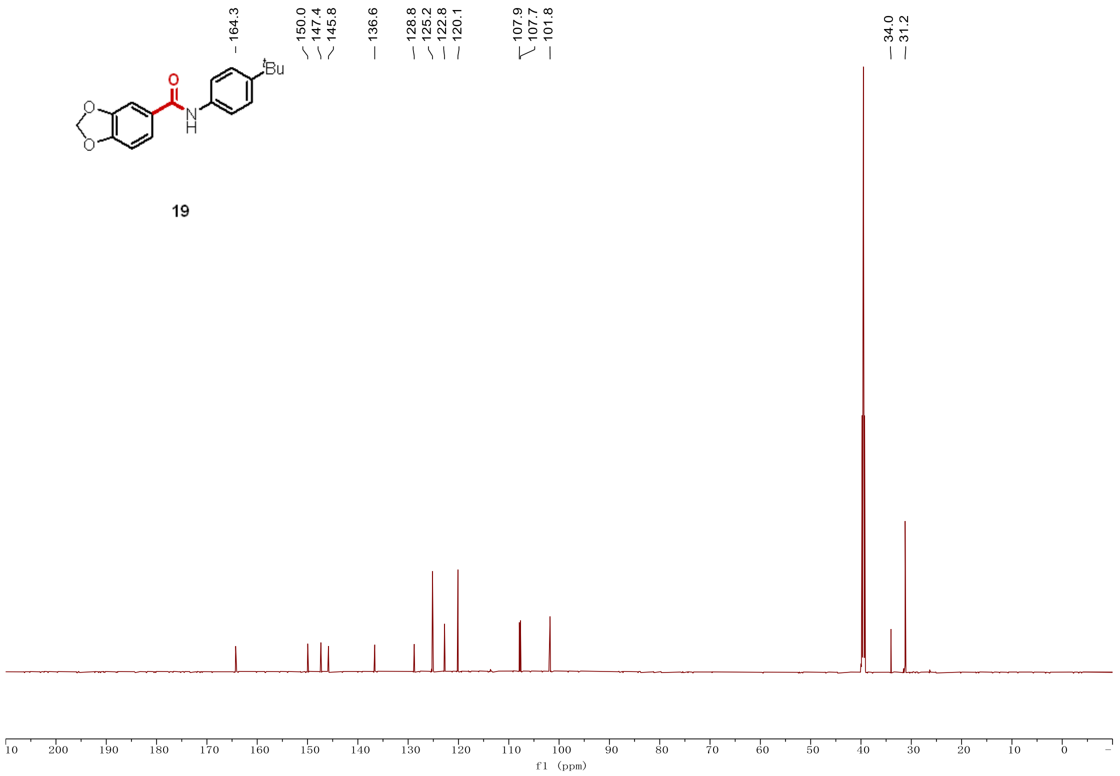

^{13}C NMR spectrum of **19** in DMSO-d_6 (176 MHz)

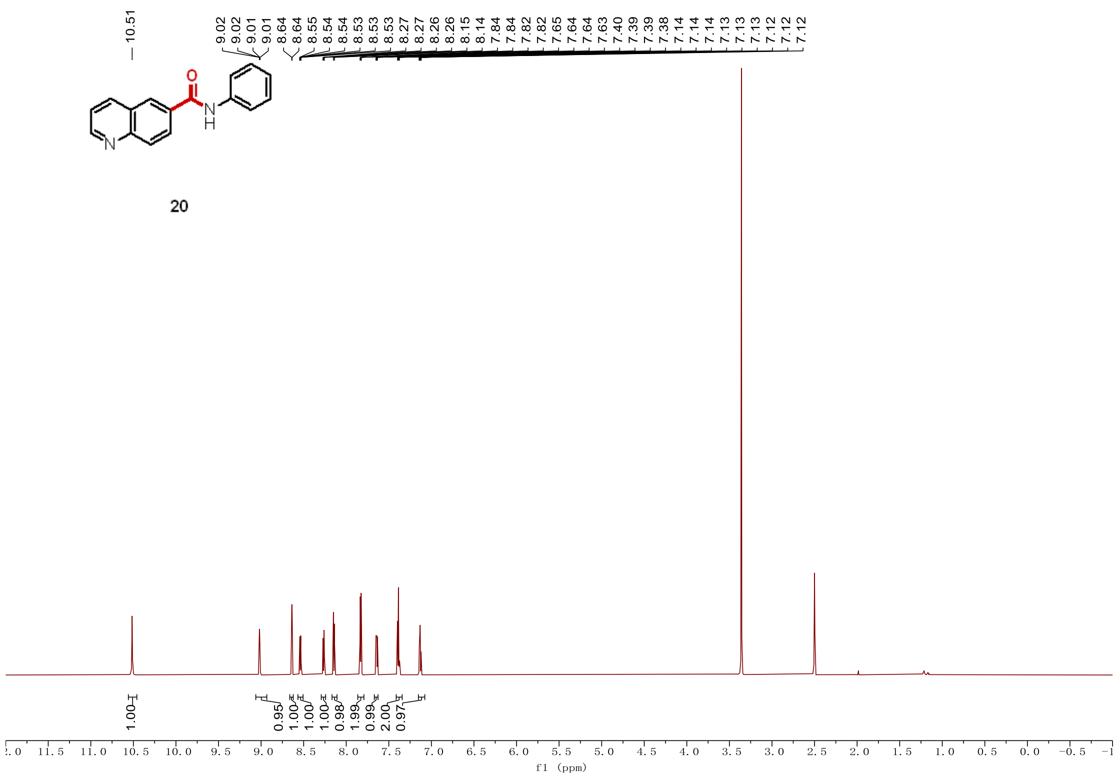

^{1}H NMR spectrum of **20** in DMSO-d_6 (700 MHz)

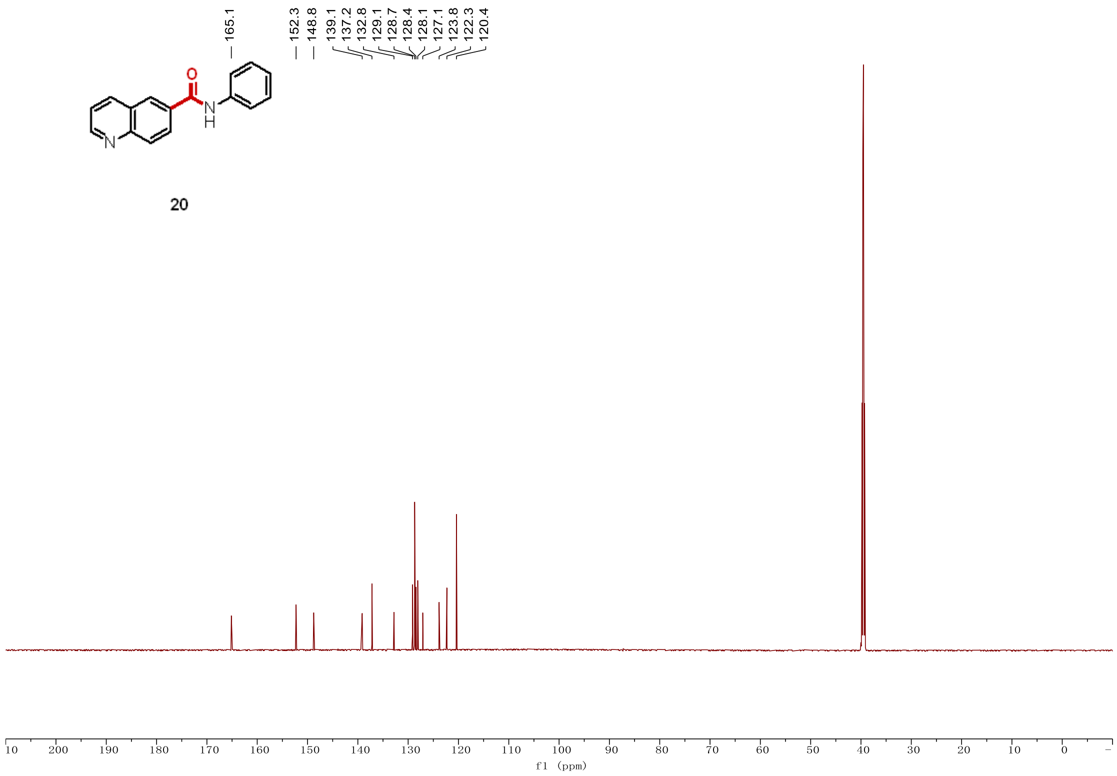

^{13}C NMR spectrum of **20** in DMSO-d_6 (176 MHz)

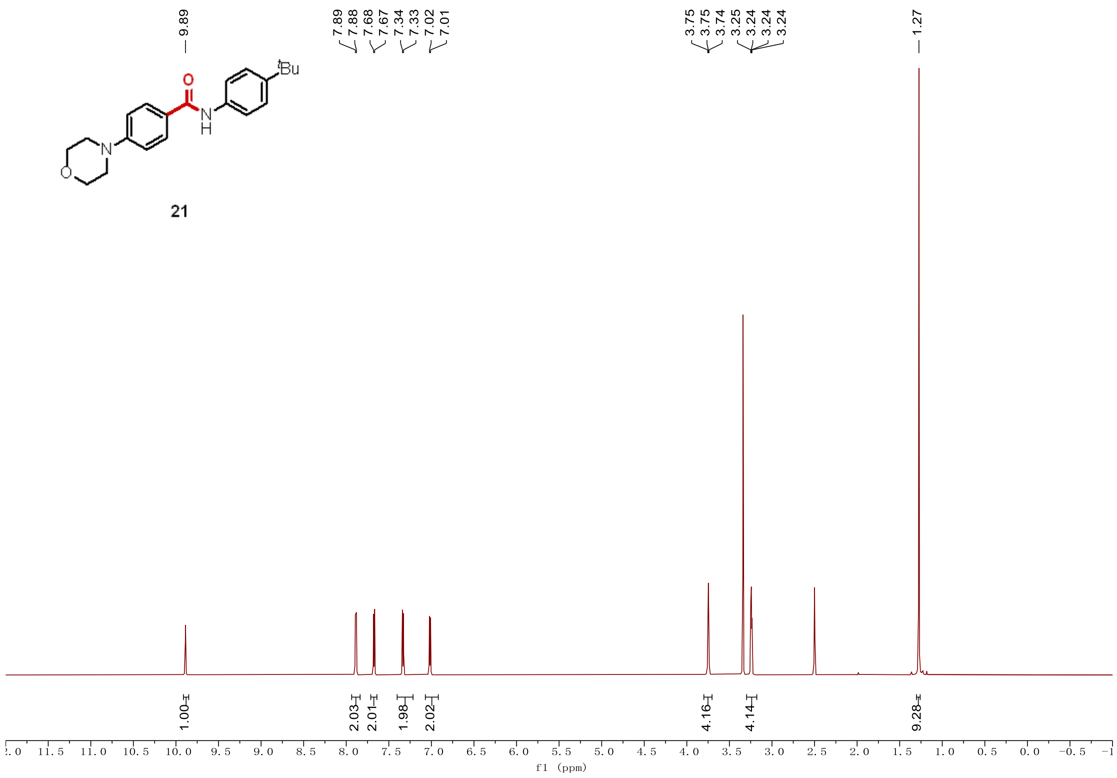

^{1}H NMR spectrum of **21** in DMSO-d_6 (700 MHz)

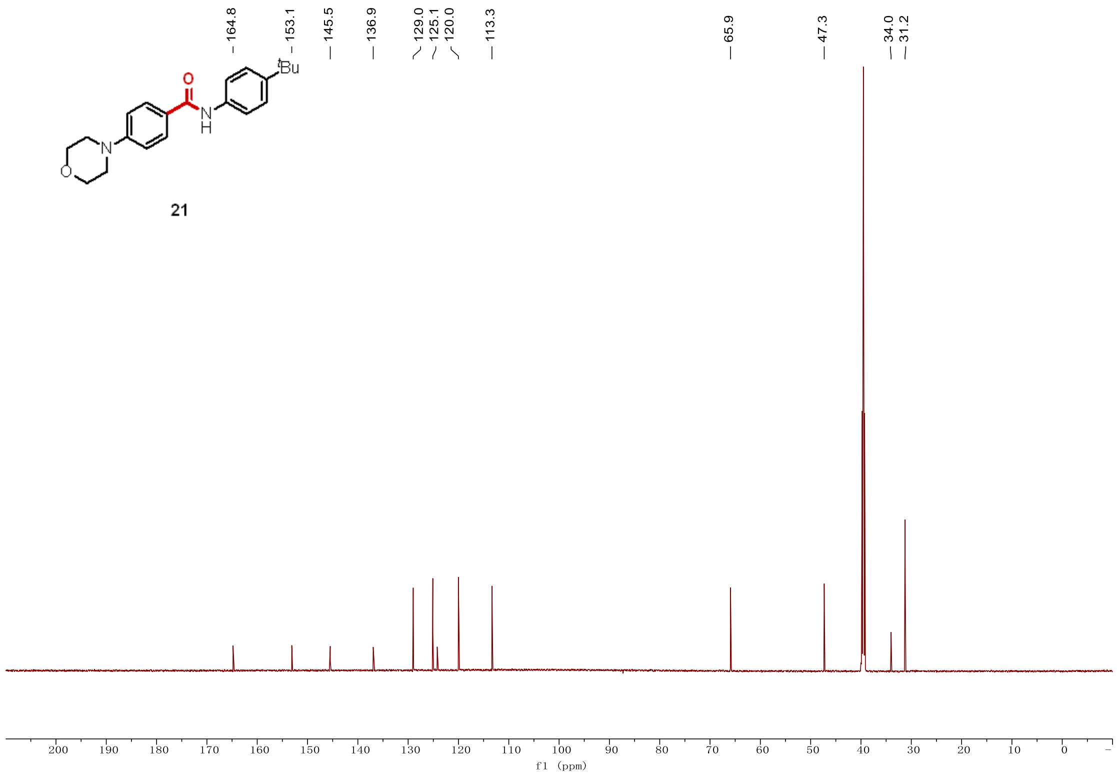

^{13}C NMR spectrum of **21** in DMSO-d_6 (176 MHz)

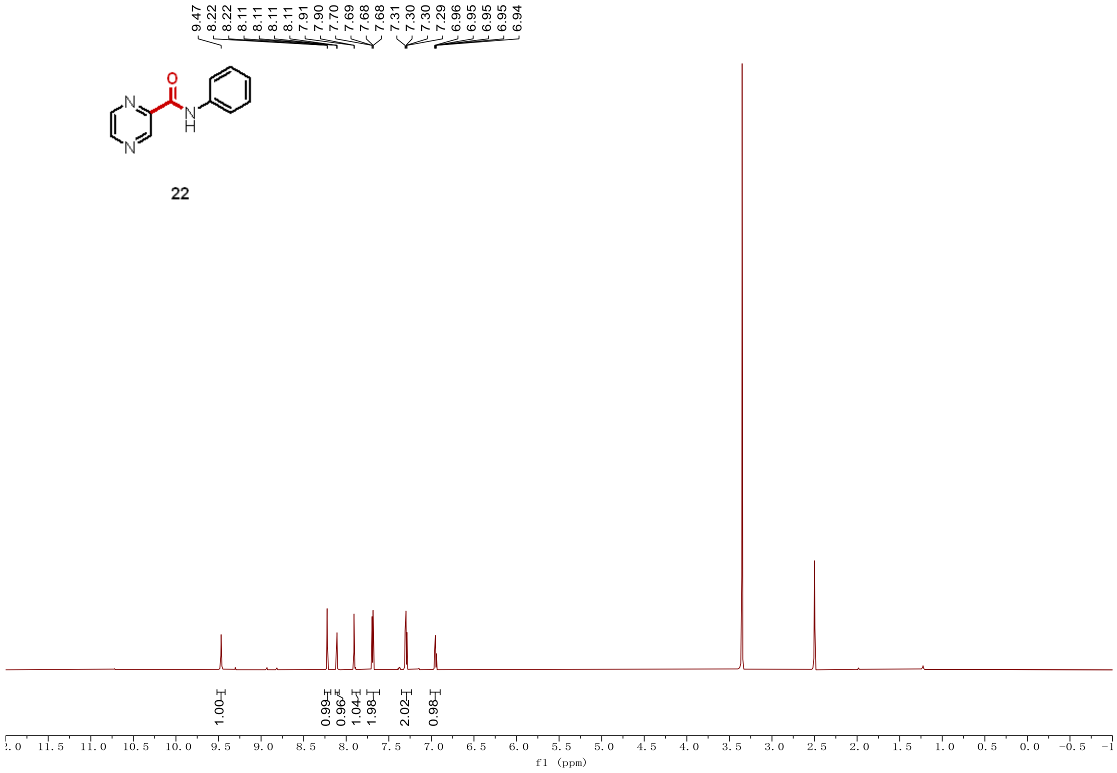

^{1}H NMR spectrum of **22** in DMSO-d_6 (700 MHz)

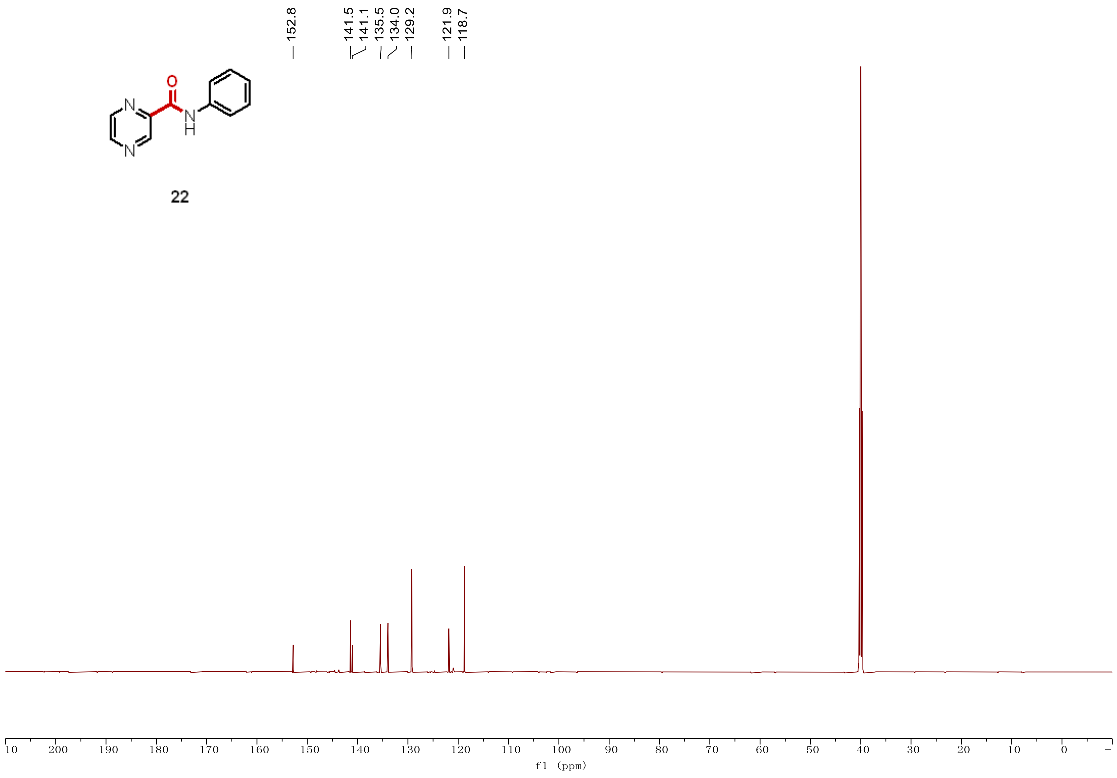

^{13}C NMR spectrum of **22** in DMSO-d_6 (176 MHz)

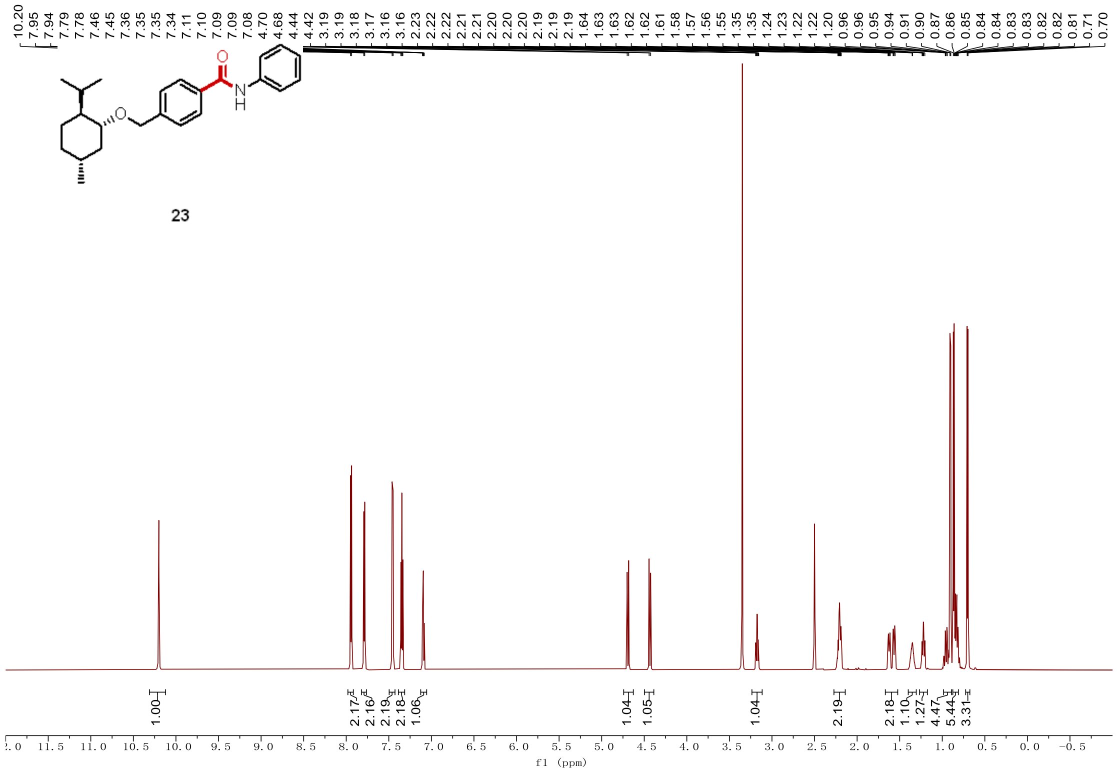

^{1}H NMR spectrum of **23** in DMSO-d_6 (700 MHz)

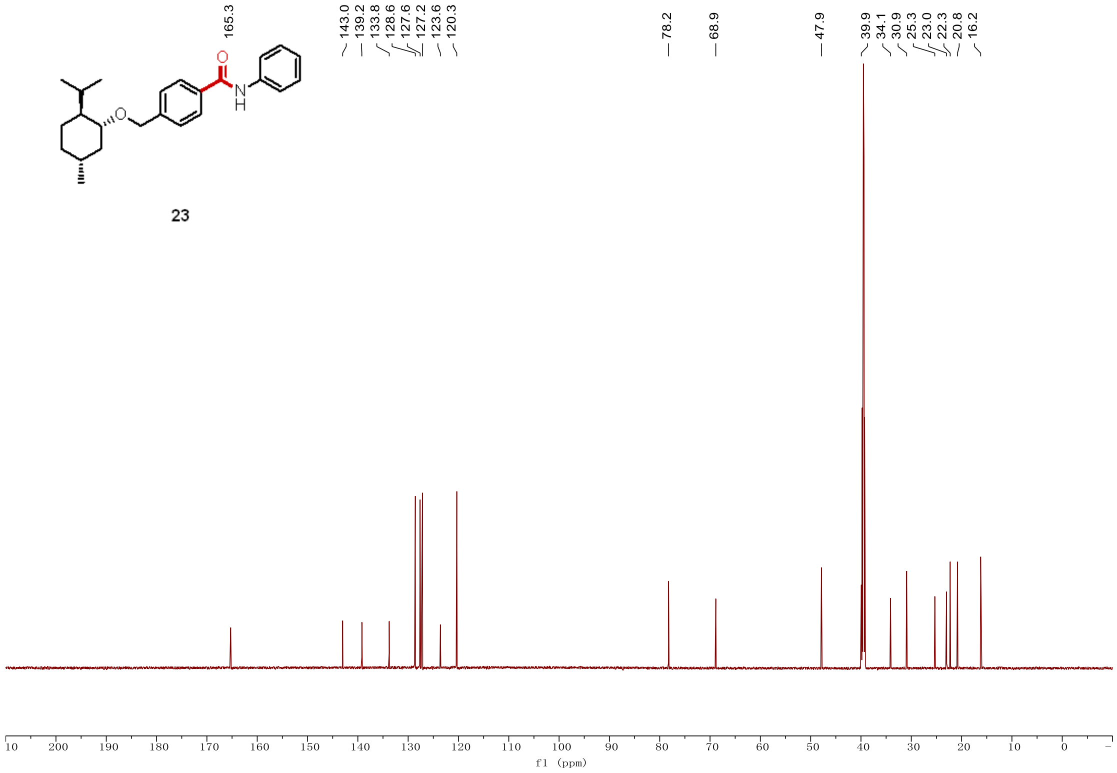

^{13}C NMR spectrum of **23** in DMSO-d_6 (176 MHz)

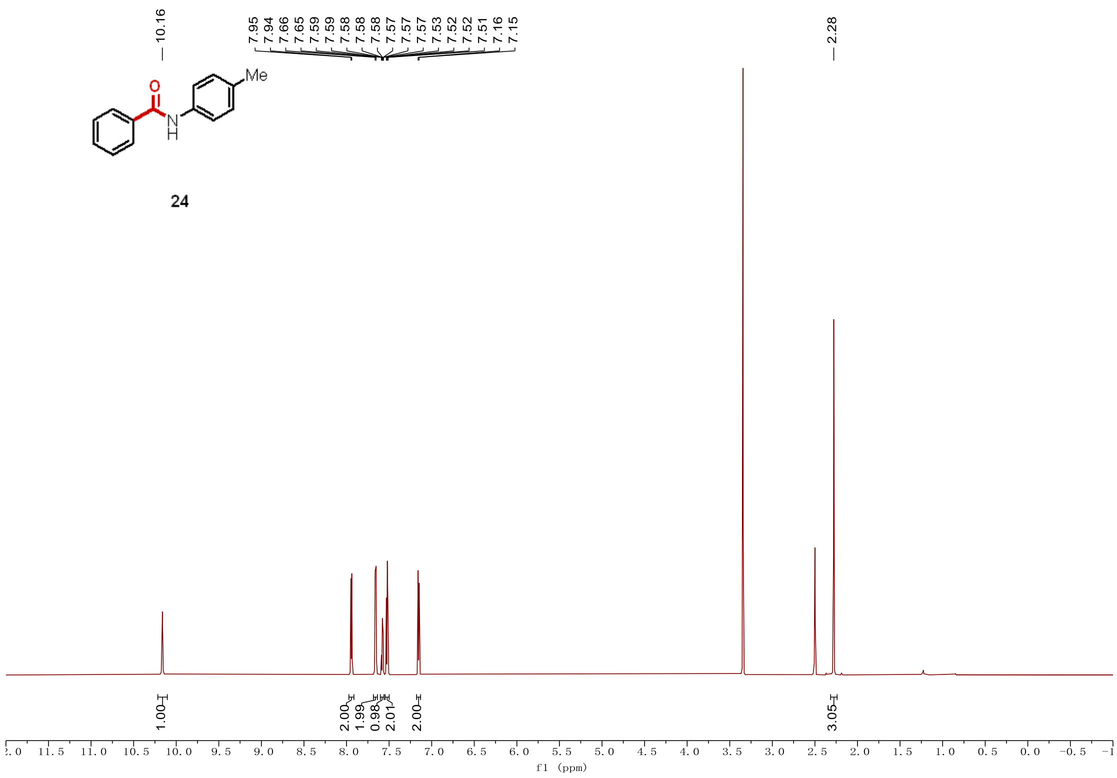

^{1}H NMR spectrum of **24** in DMSO-d_6 (700 MHz)

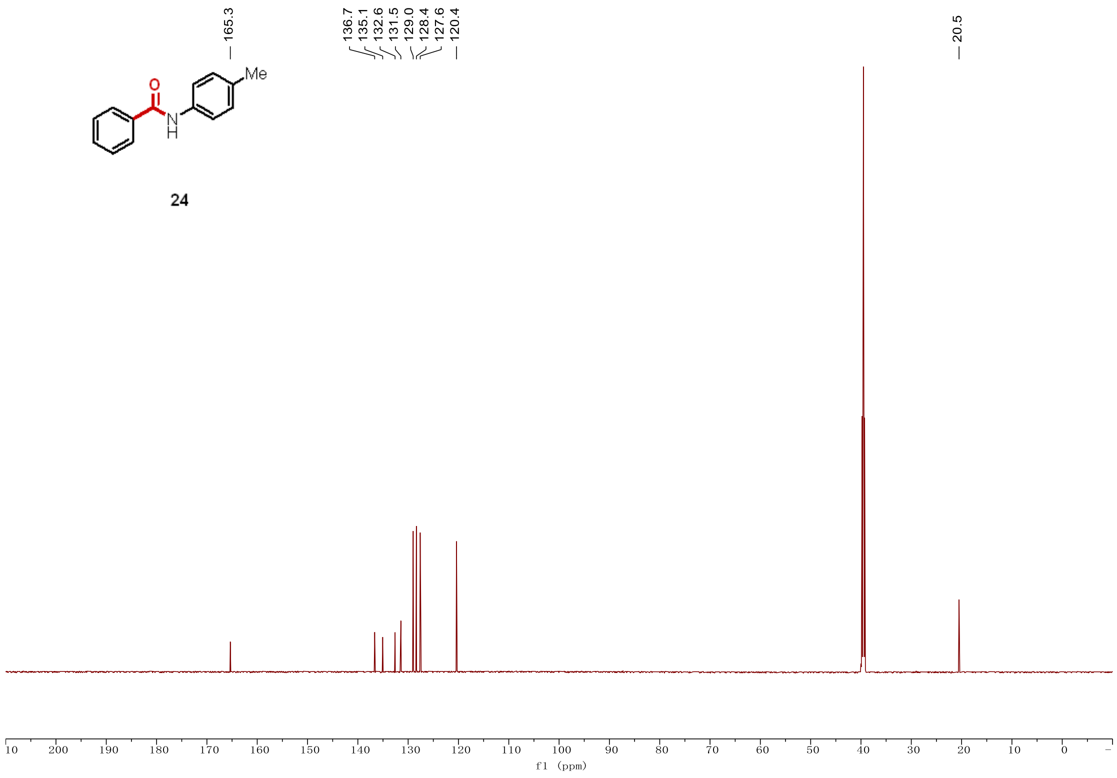

^{13}C NMR spectrum of **24** in DMSO-d_6 (176 MHz)

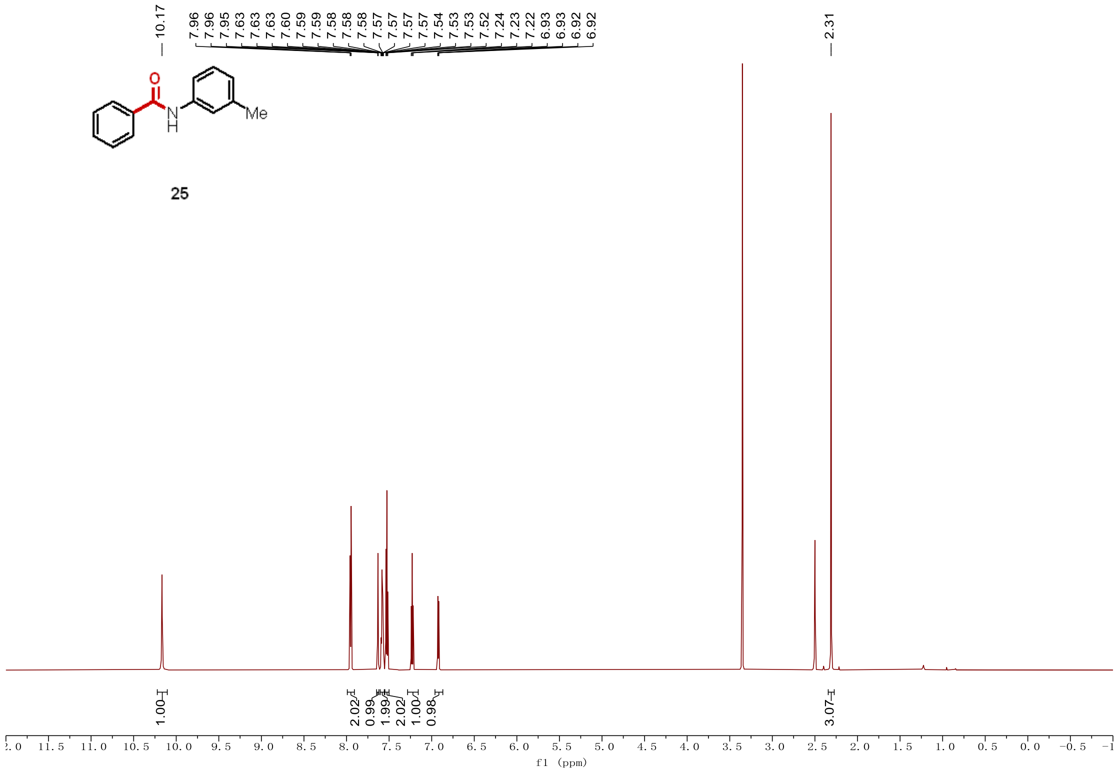

^{1}H NMR spectrum of **25** in DMSO-d_6 (700 MHz)

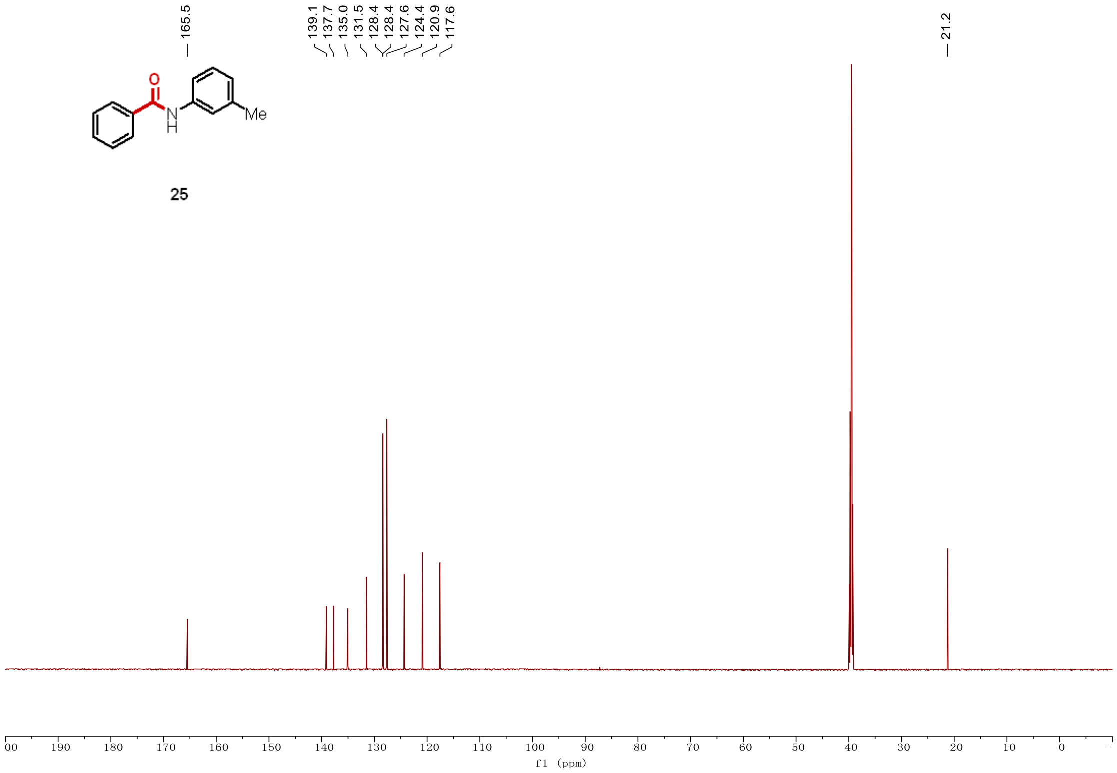

^{13}C NMR spectrum of **25** in DMSO-d_6 (176 MHz)

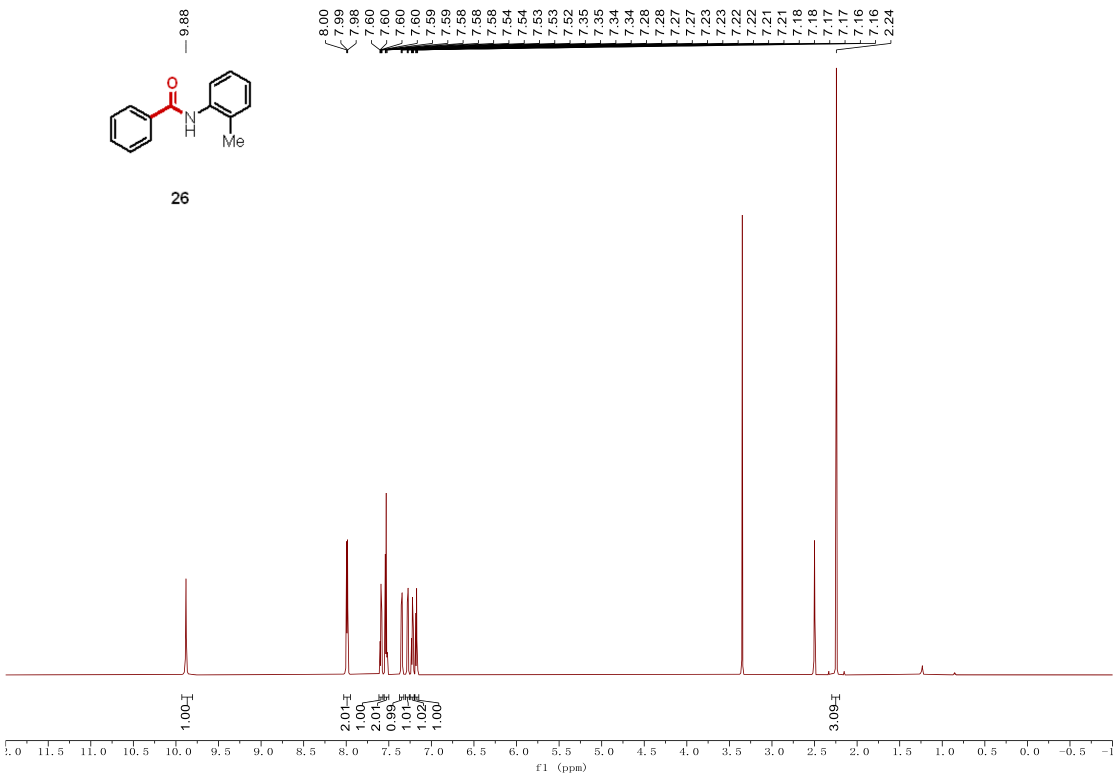

^{1}H NMR spectrum of **26** in DMSO-d_6 (700 MHz)

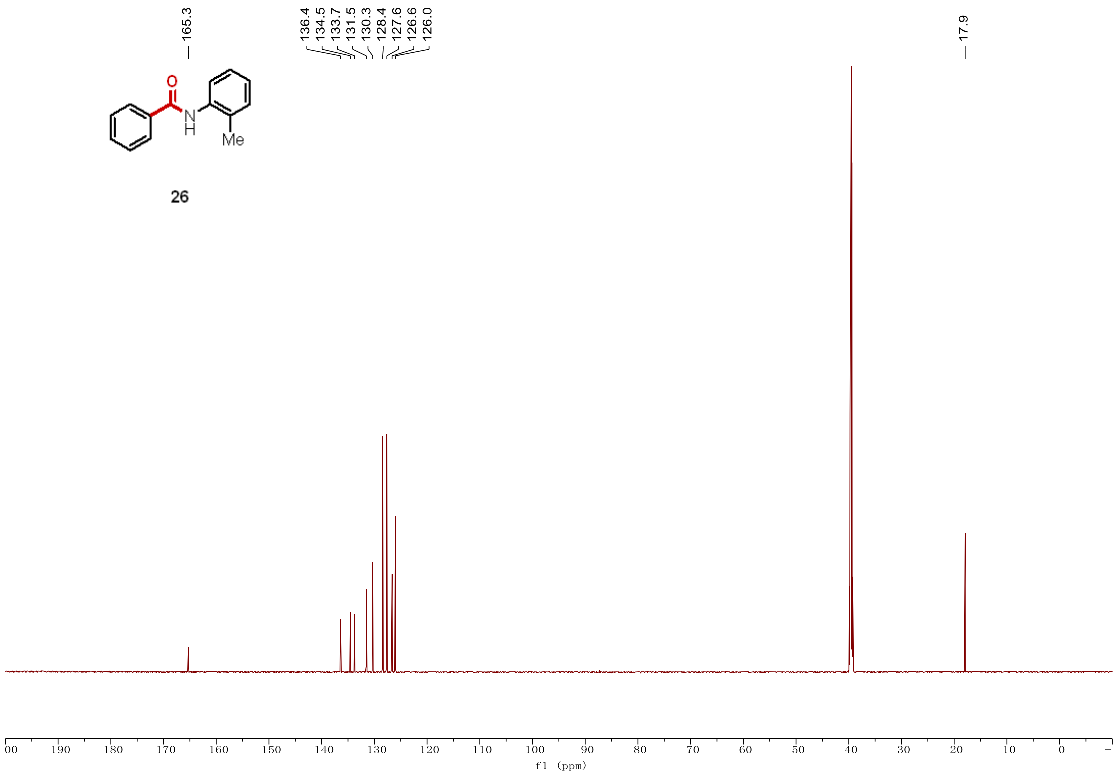

^{13}C NMR spectrum of **26** in DMSO-d_6 (176 MHz)

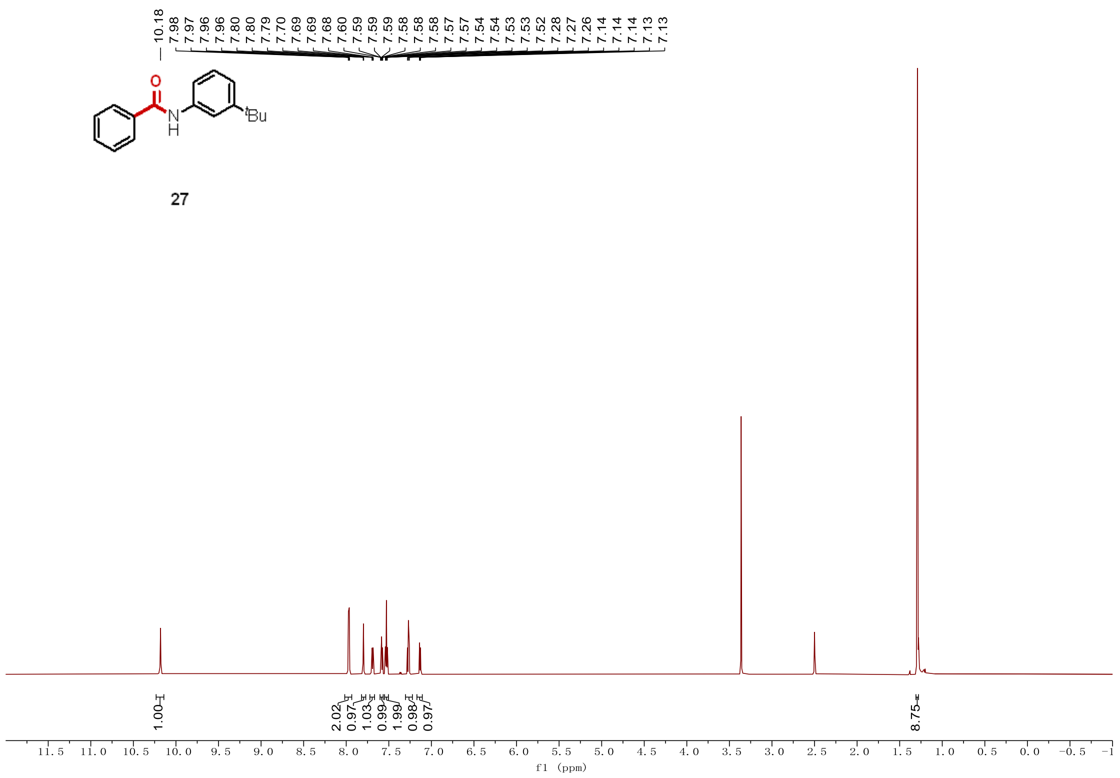

^{1}H NMR spectrum of **27** in DMSO-d_6 (700 MHz)

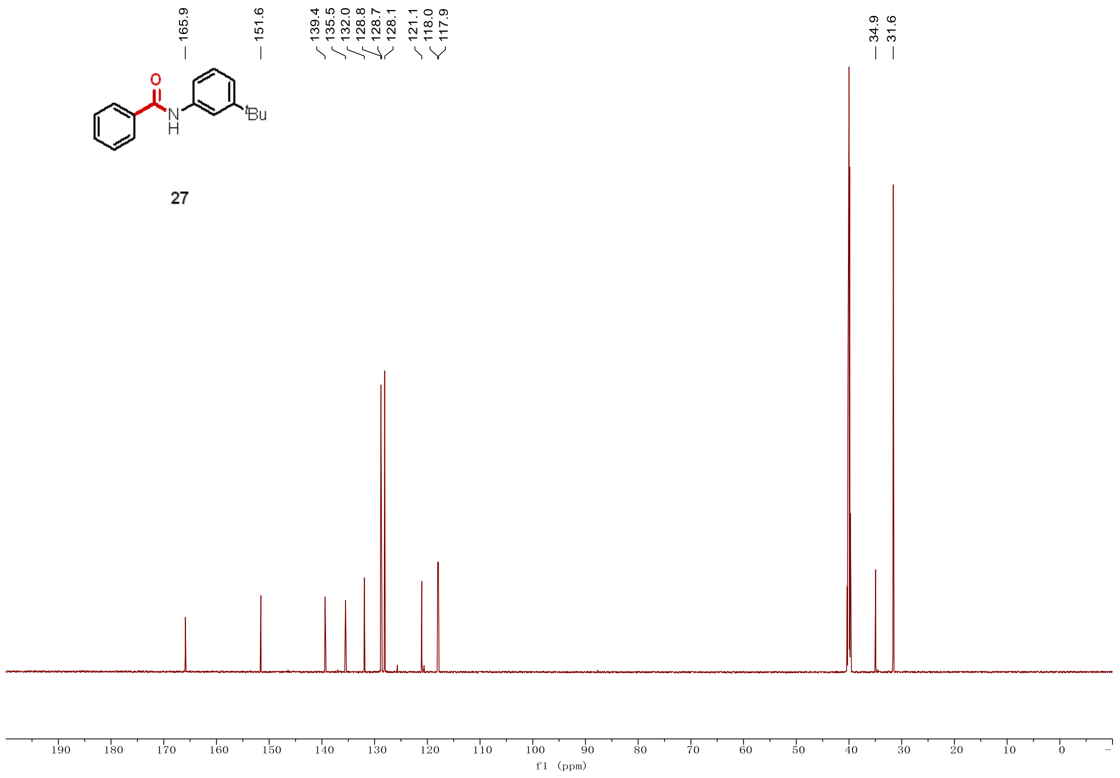

^{13}C NMR spectrum of **27** in DMSO-d_6 (176 MHz)

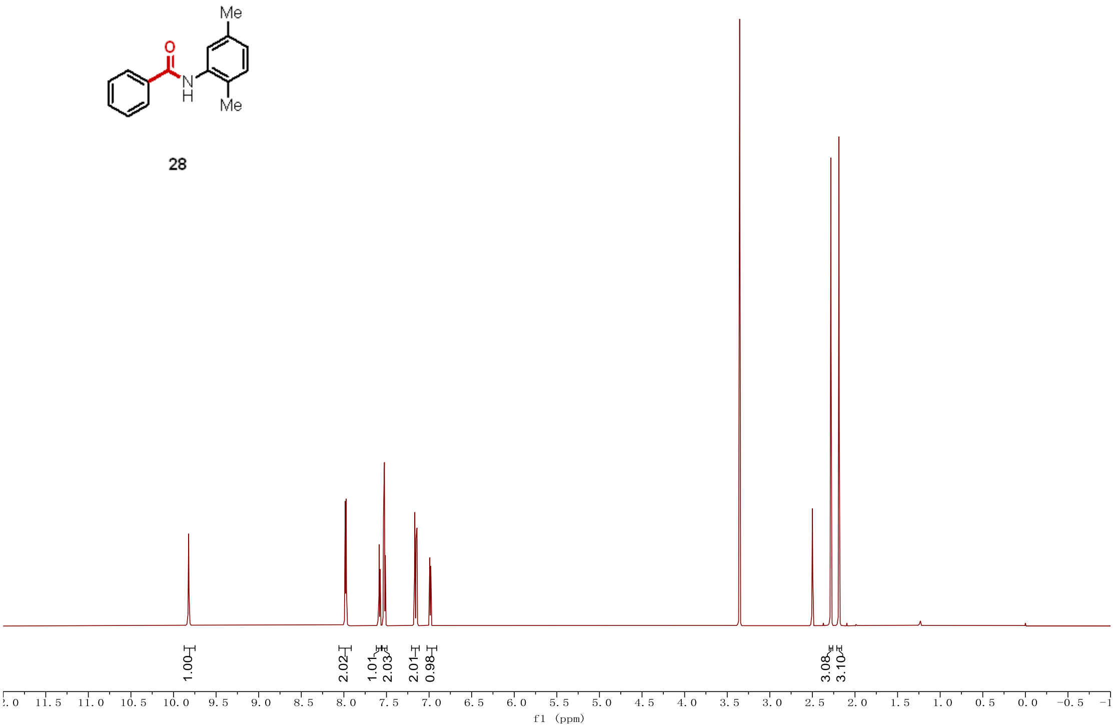

^{1}H NMR spectrum of **28** in DMSO-d_6 (700 MHz)

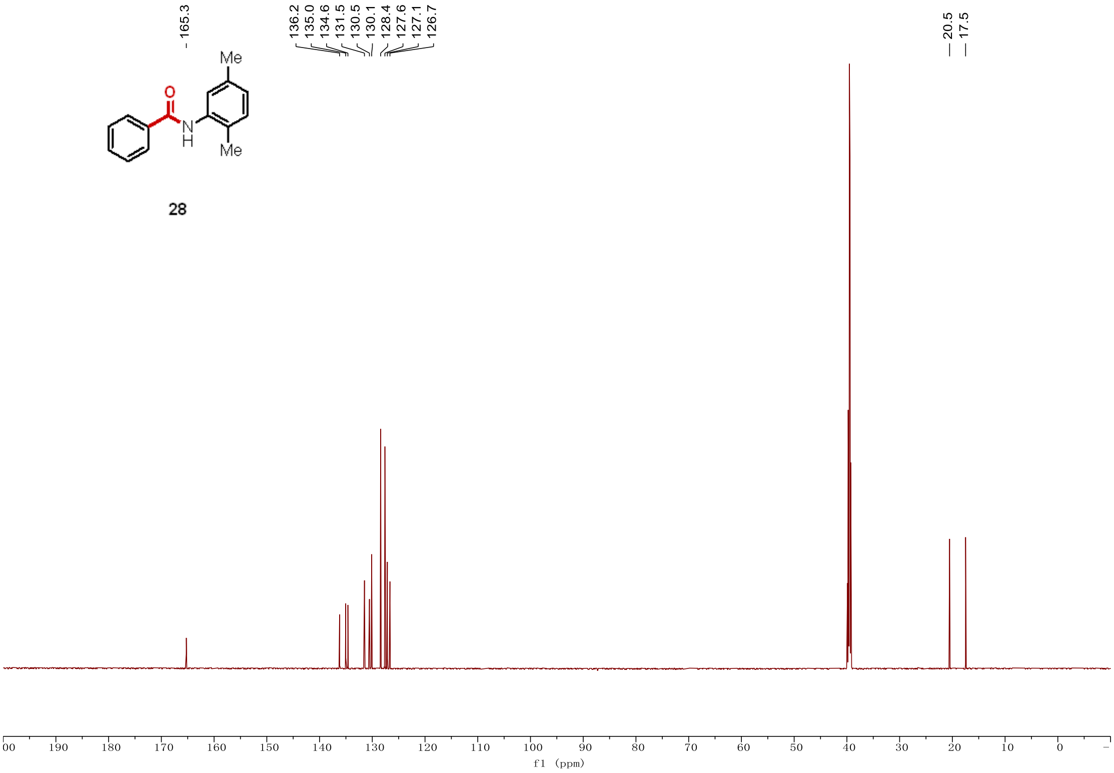

^{13}C NMR spectrum of **28** in DMSO-d_6 (176 MHz)

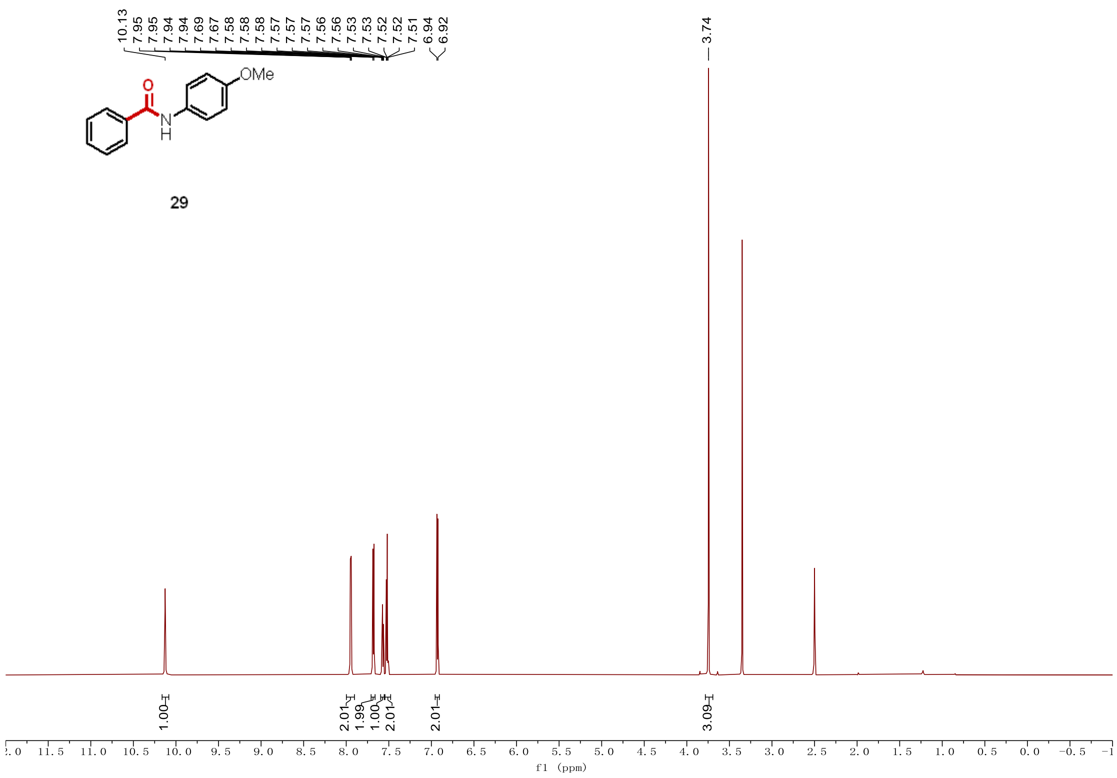

^{1}H NMR spectrum of **29** in DMSO-d_6 (700 MHz)

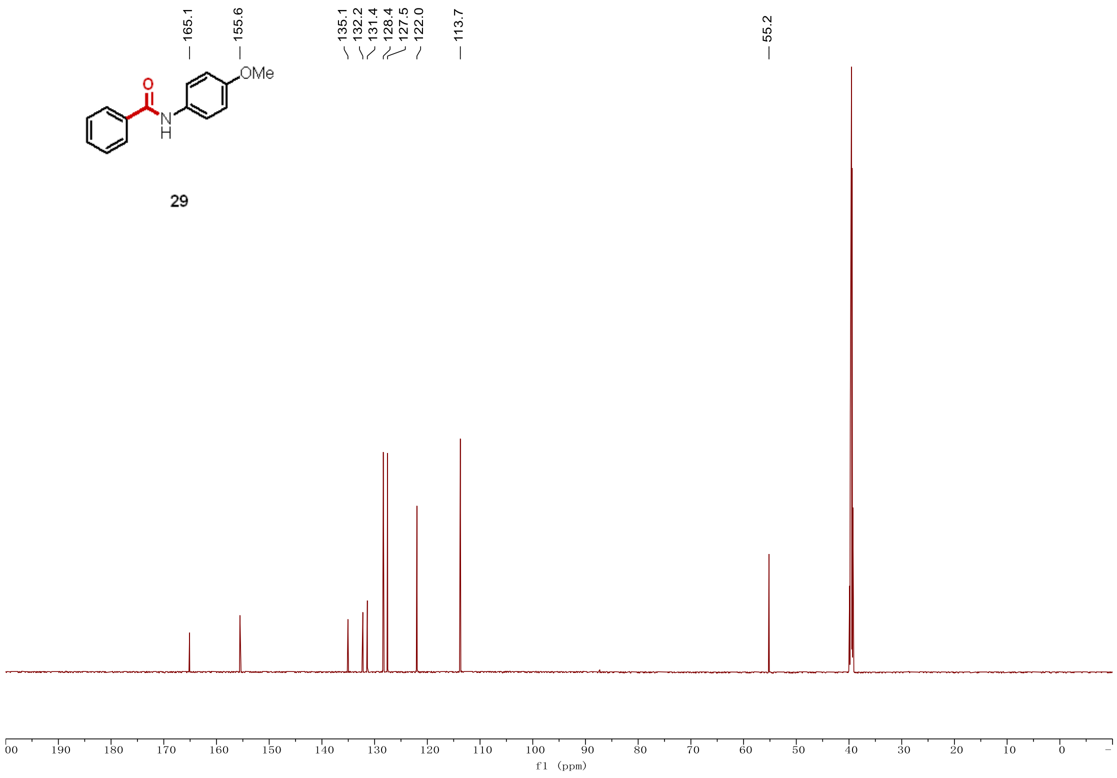

^{13}C NMR spectrum of **29** in DMSO-d_6 (176 MHz)

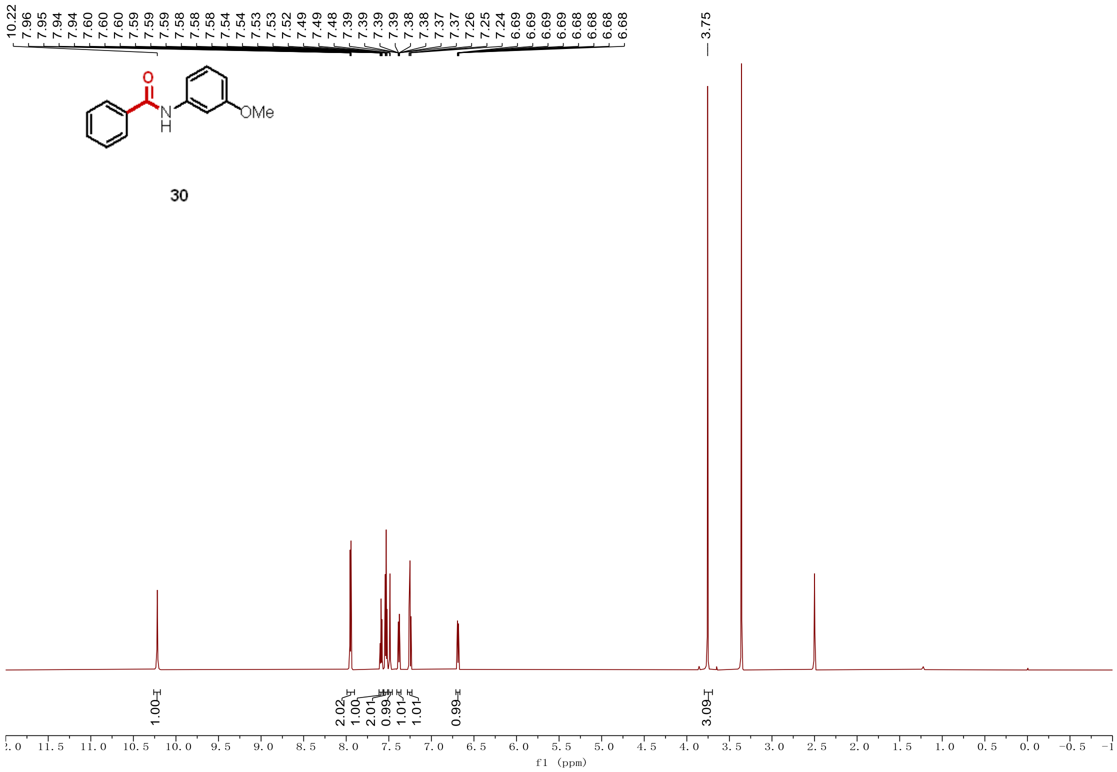

^{1}H NMR spectrum of **30** in DMSO-d_6 (700 MHz)

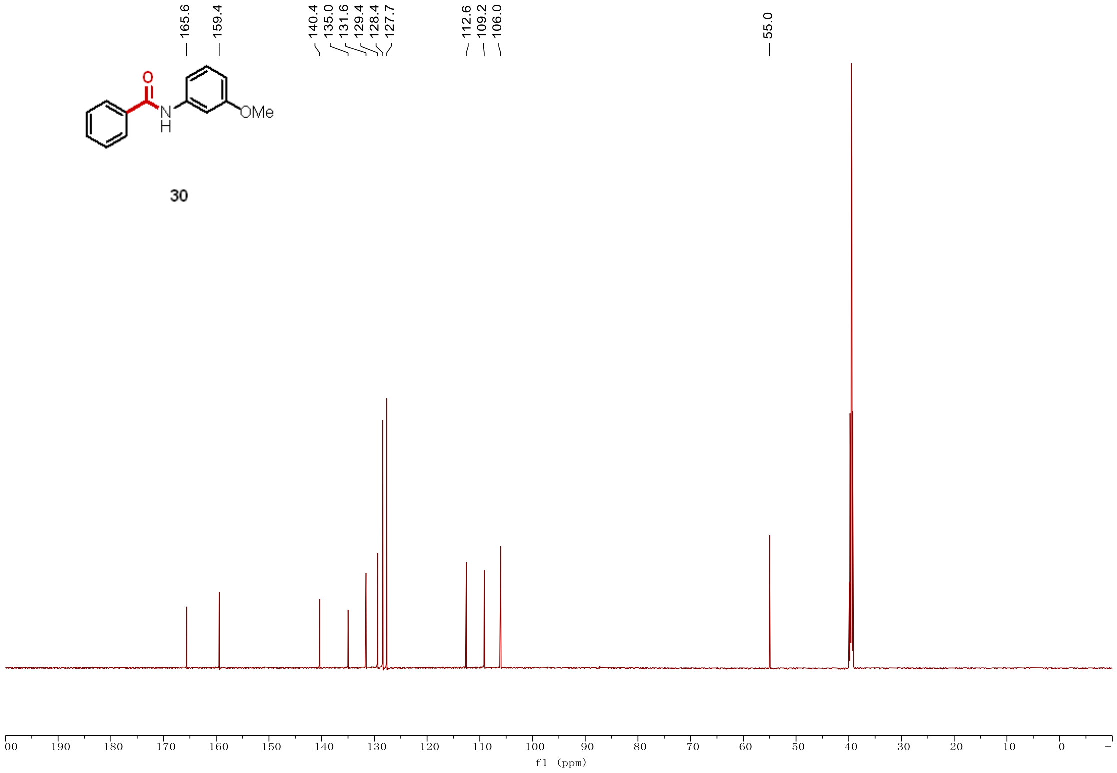

^{13}C NMR spectrum of **30** in DMSO-d_6 (176 MHz)

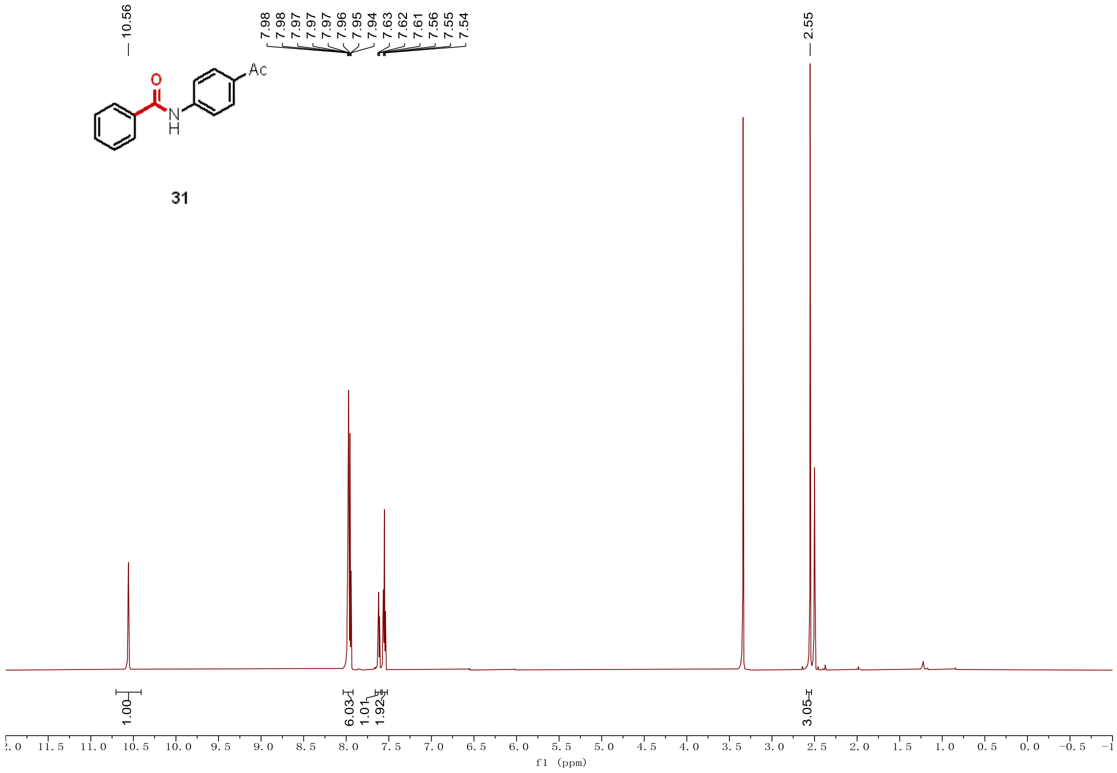

^{1}H NMR spectrum of **31** in DMSO-d_6 (700 MHz)

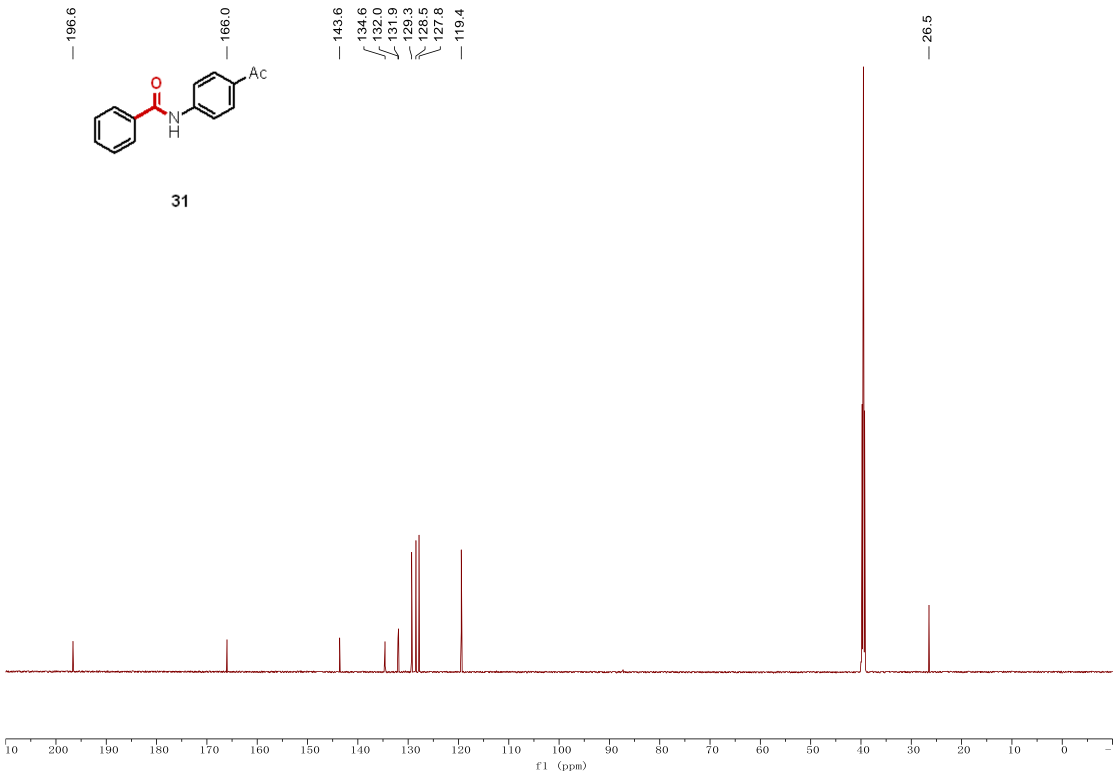

^{13}C NMR spectrum of **31** in DMSO-d_6 (176 MHz)

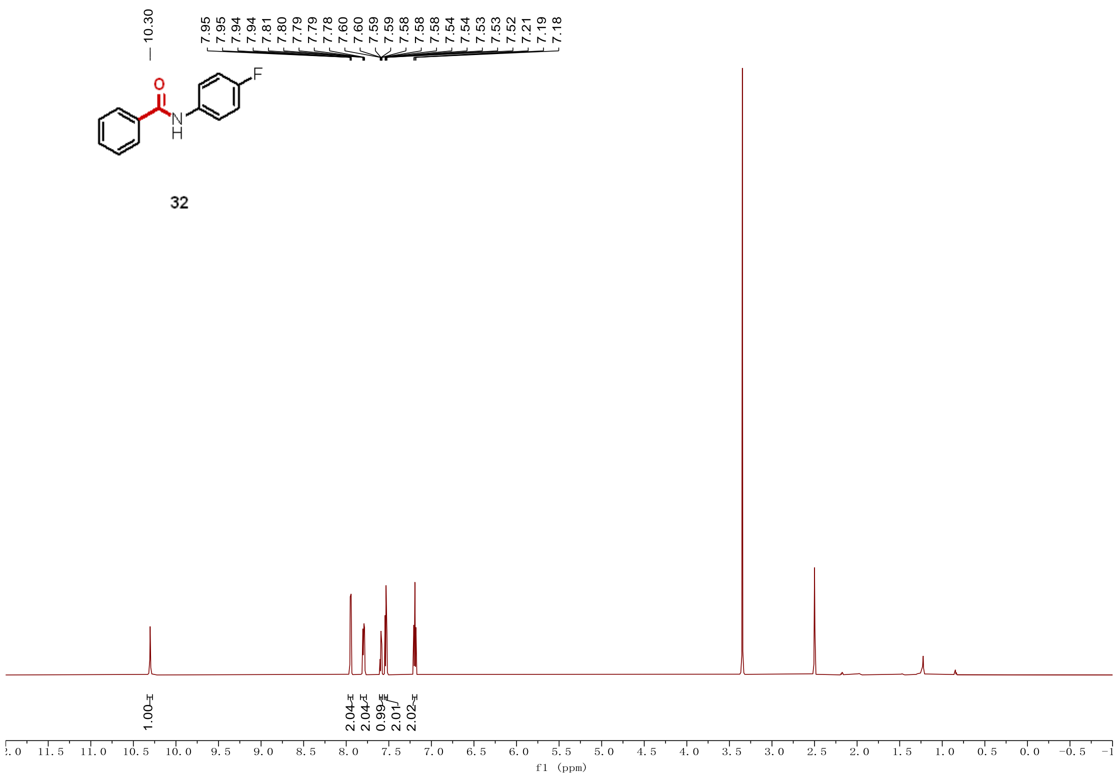

^{1}H NMR spectrum of **32** in DMSO-d_6 (700 MHz)

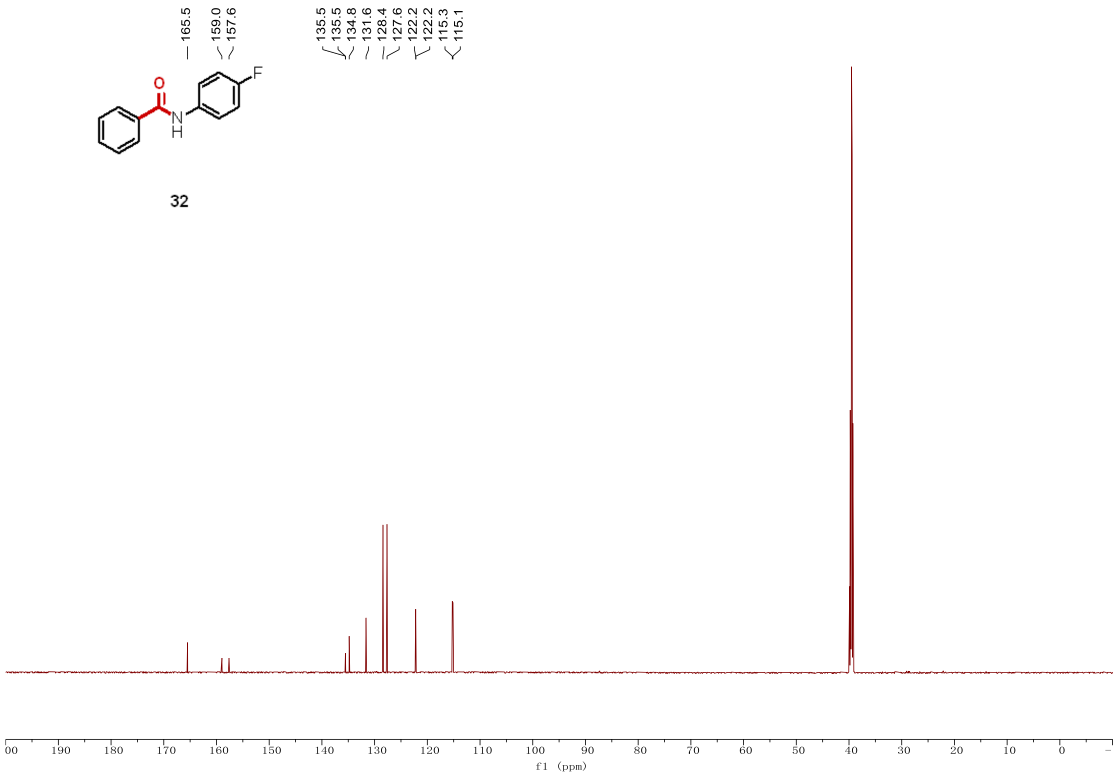

^{13}C NMR spectrum of **32** in DMSO-d_6 (176 MHz)

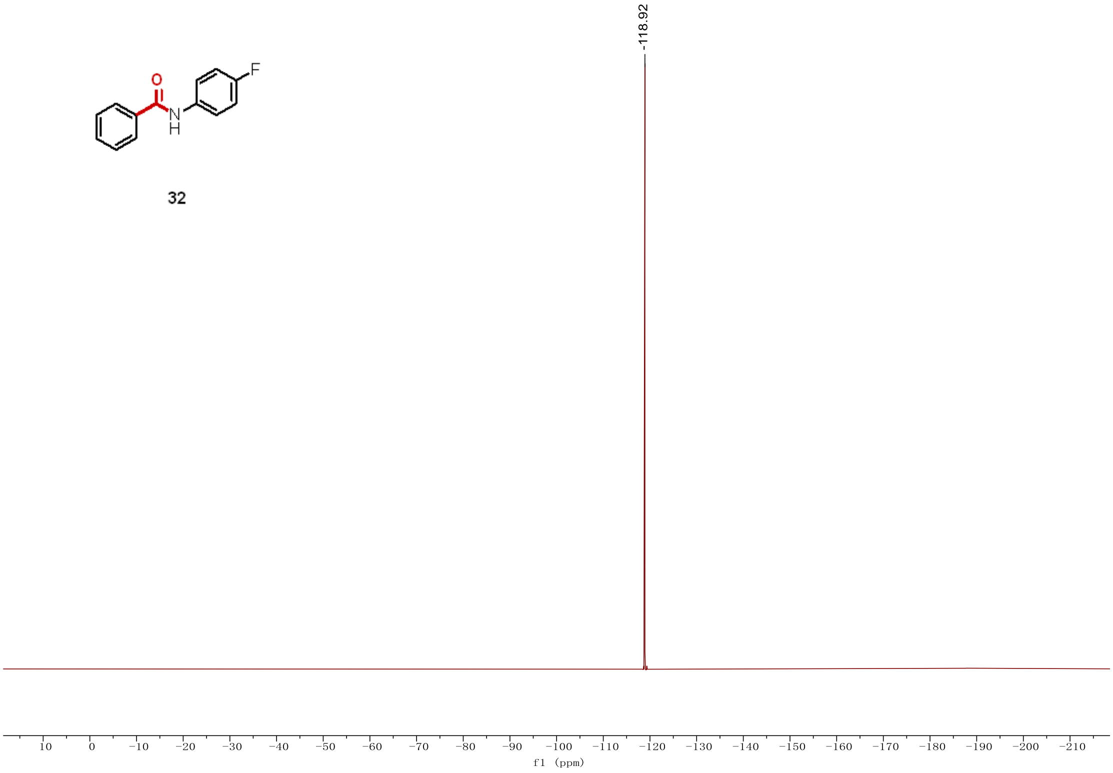

32

^{19}F NMR spectrum of **32** in DMSO-d_6 (376 MHz)

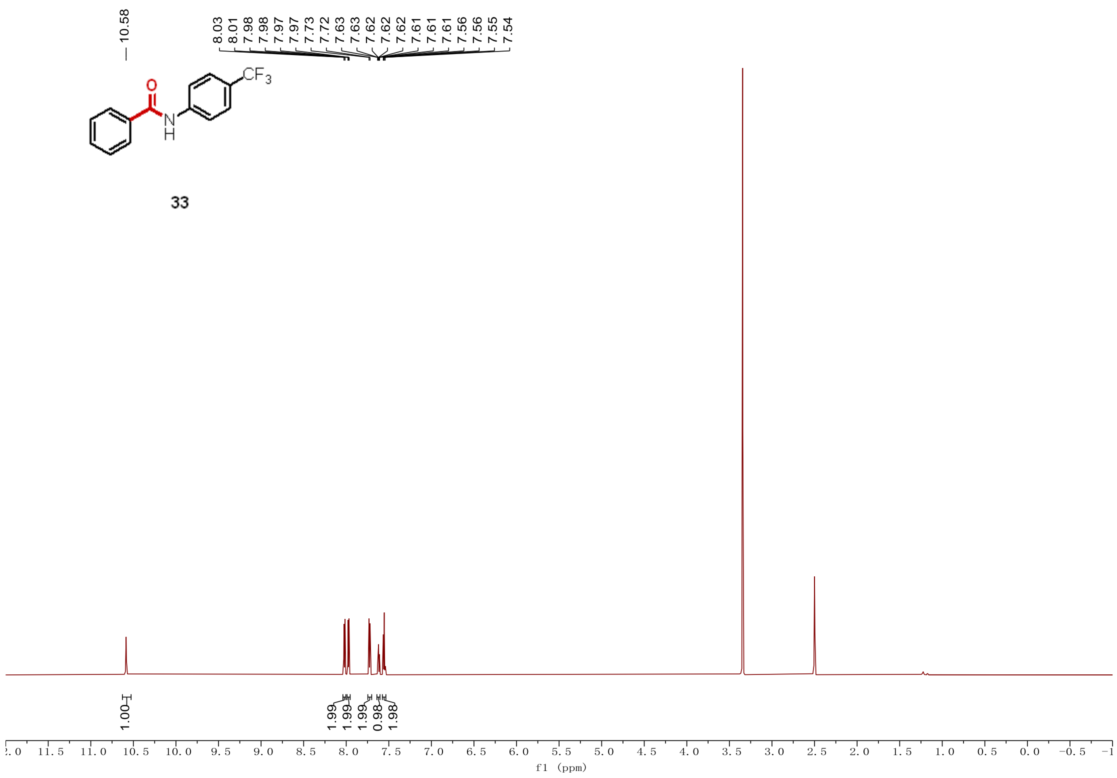

^{1}H NMR spectrum of **33** in DMSO-d_6 (700 MHz)

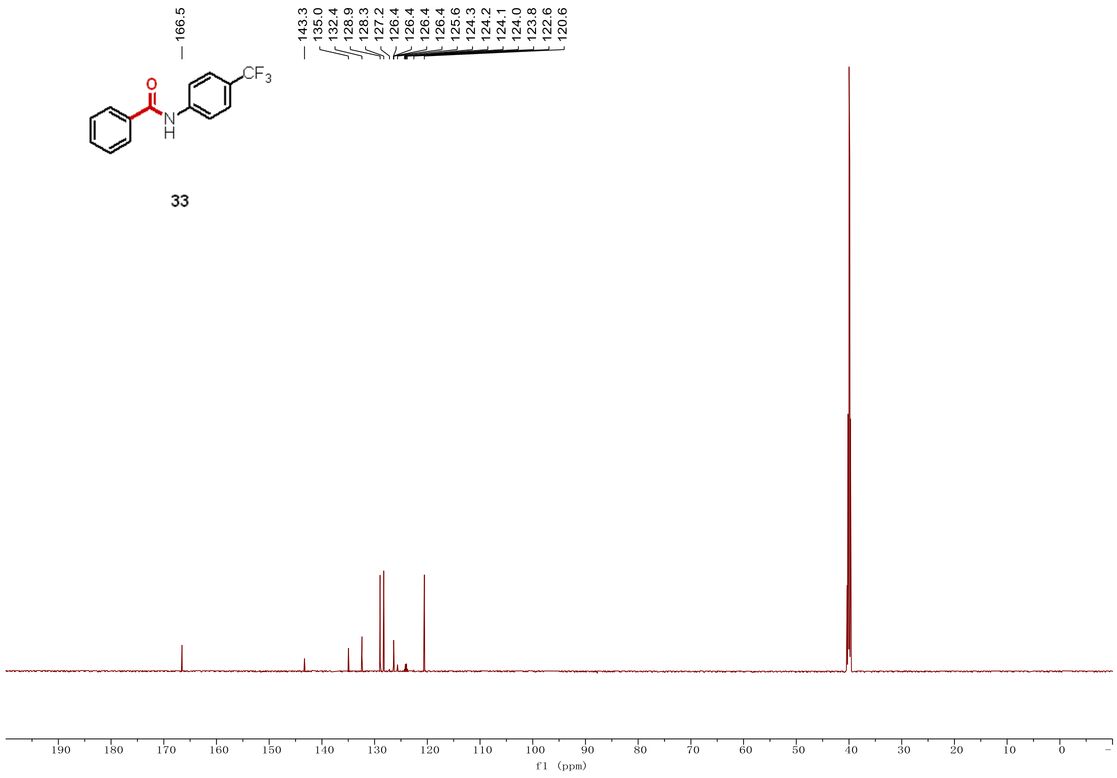

^{13}C NMR spectrum of **33** in DMSO-d_6 (176 MHz)

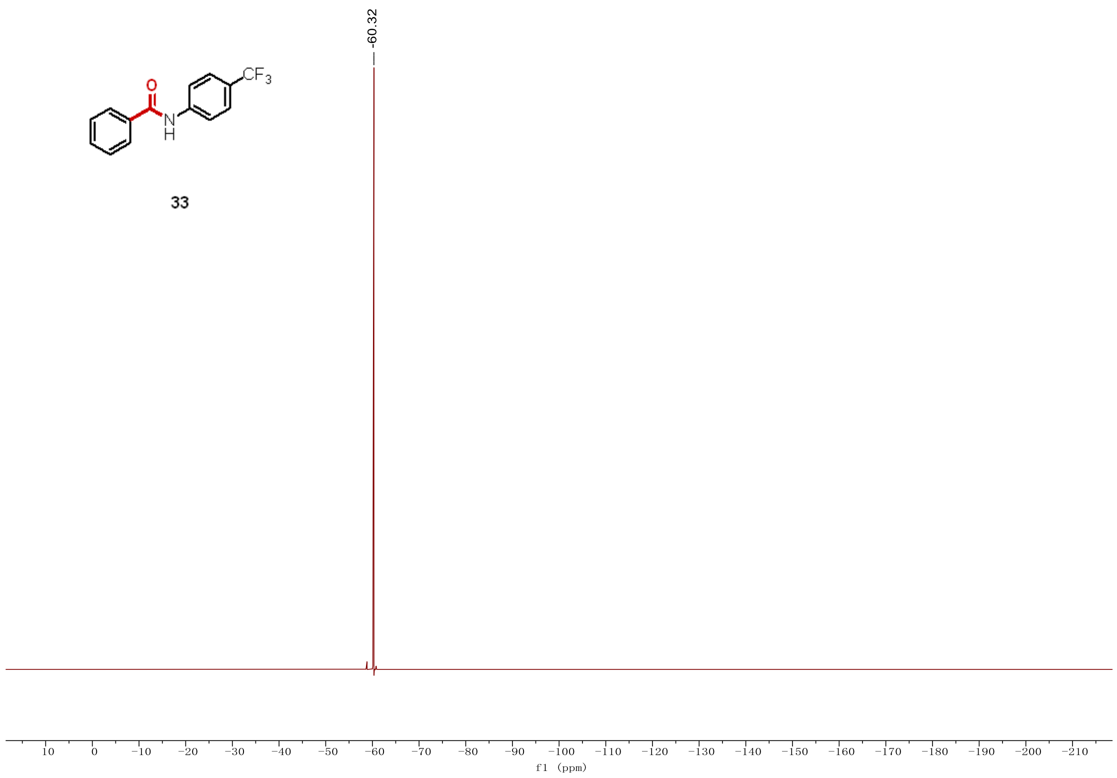

^{19}F NMR spectrum of **33** in DMSO-*d*$_6$ (376 MHz)

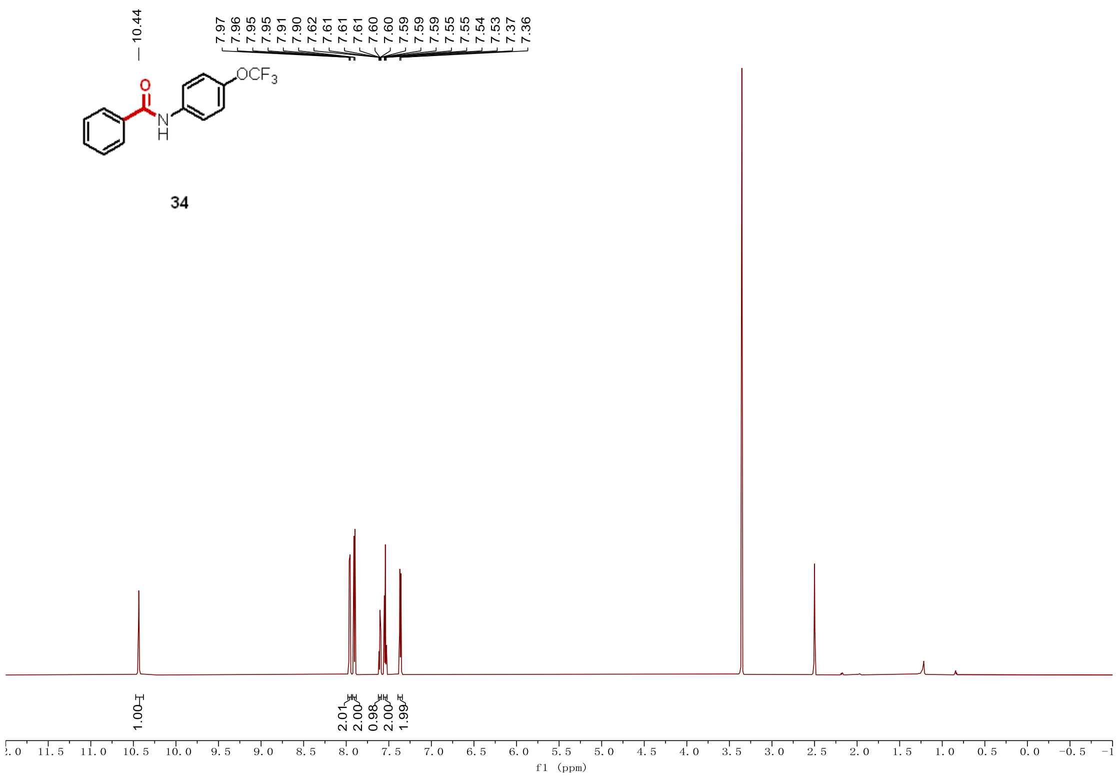

^{1}H NMR spectrum of **34** in DMSO-d_6 (700 MHz)

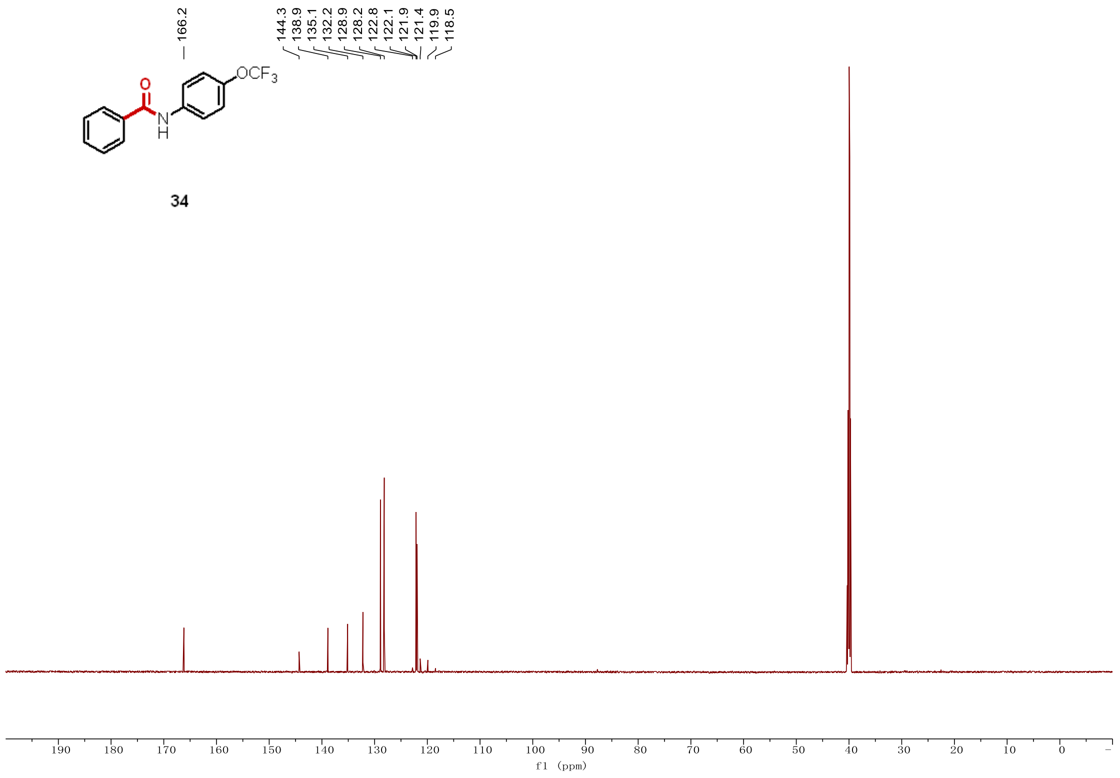

^{13}C NMR spectrum of **34** in DMSO-d_6 (176 MHz)

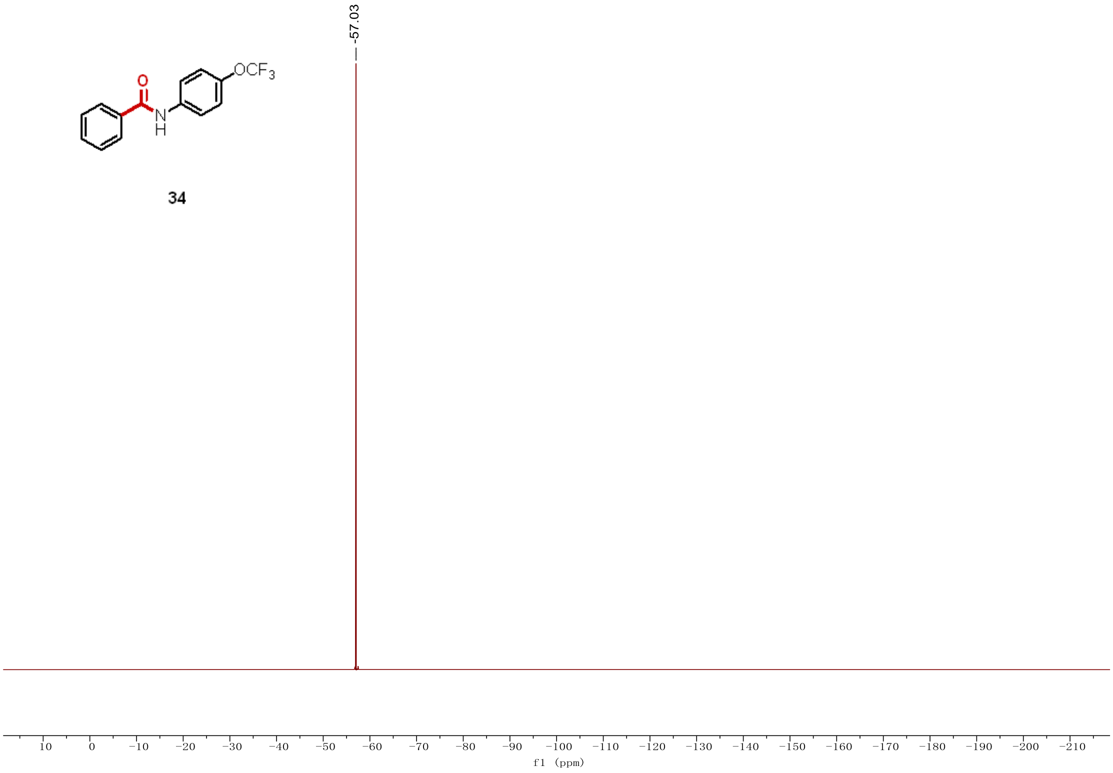

^{19}F NMR spectrum of **34** in DMSO-d_6 (376 MHz)

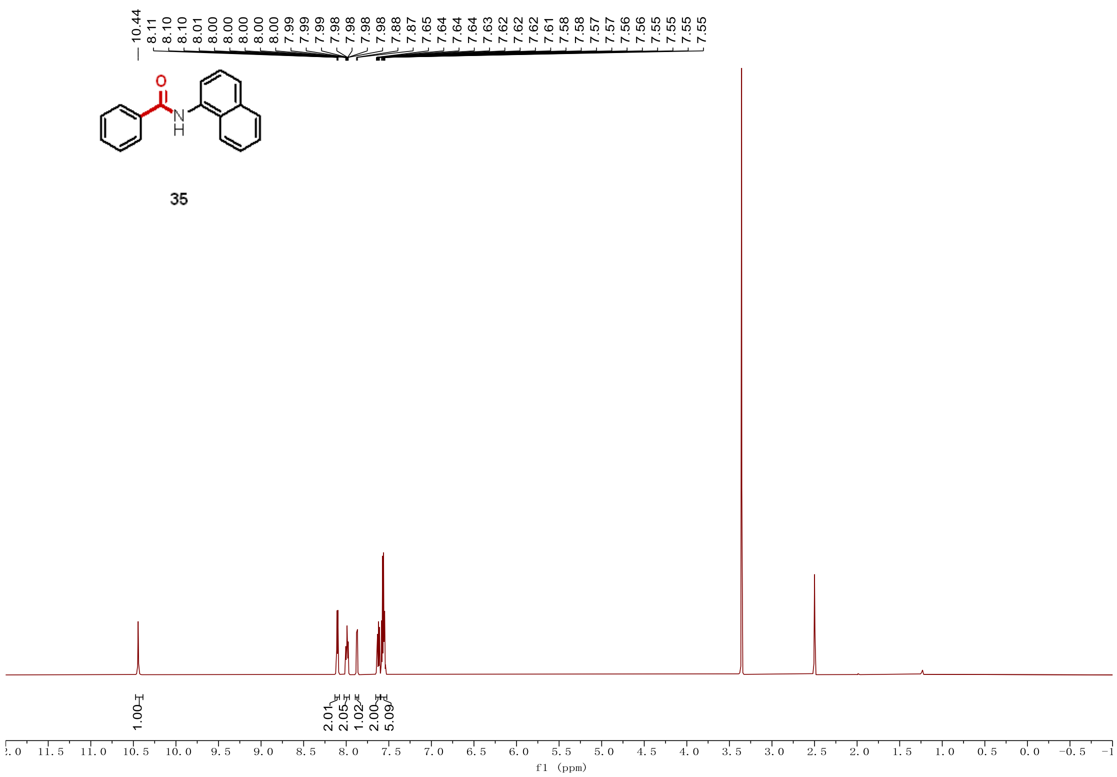

^{1}H NMR spectrum of **35** in DMSO-d_6 (700 MHz)

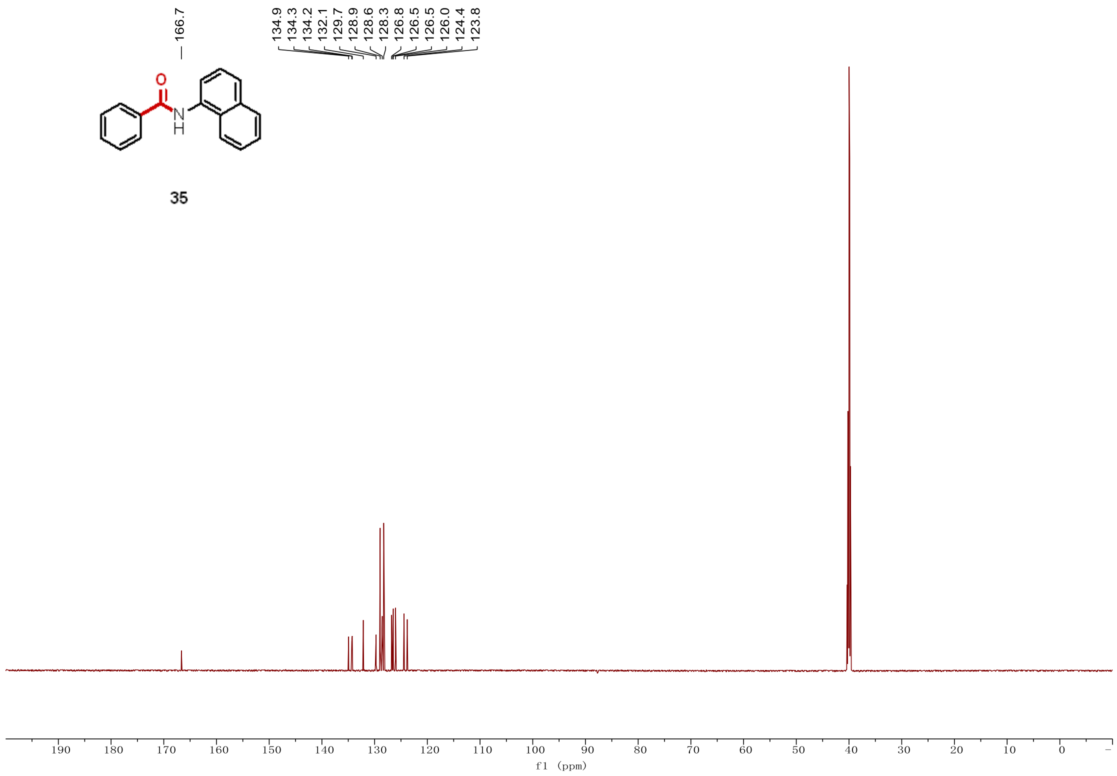

^{13}C NMR spectrum of **35** in DMSO-d_6 (176 MHz)

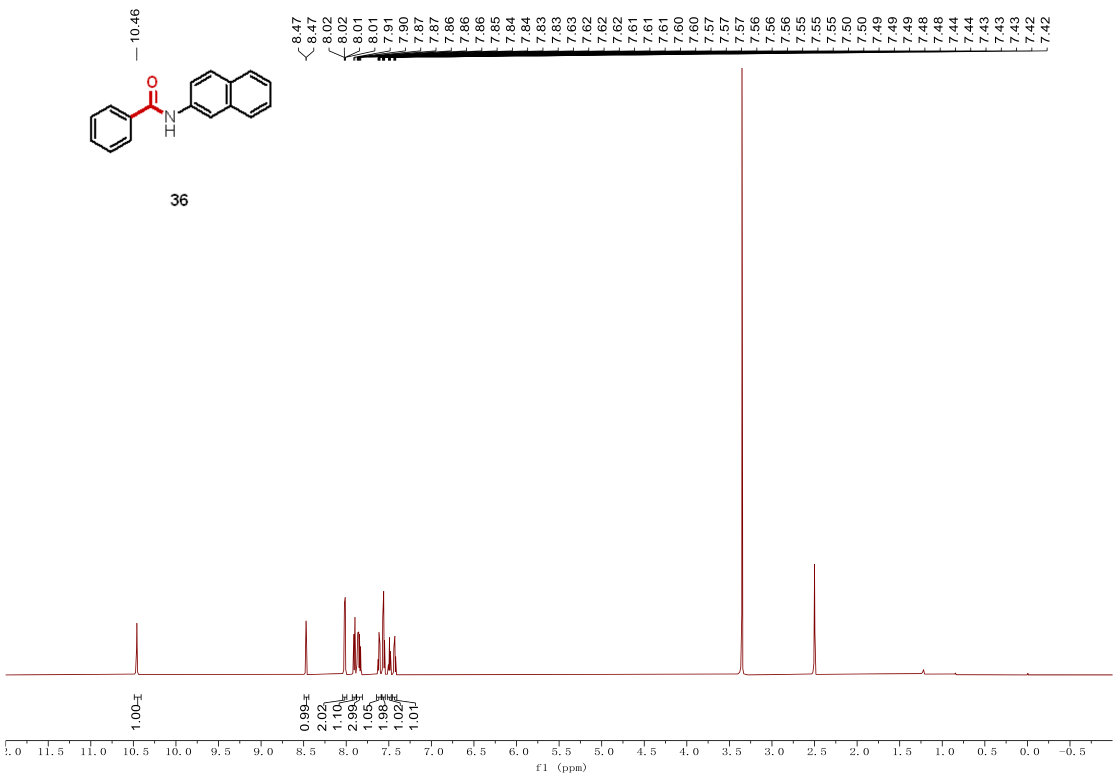

^{1}H NMR spectrum of **36** in DMSO-d_6 (700 MHz)

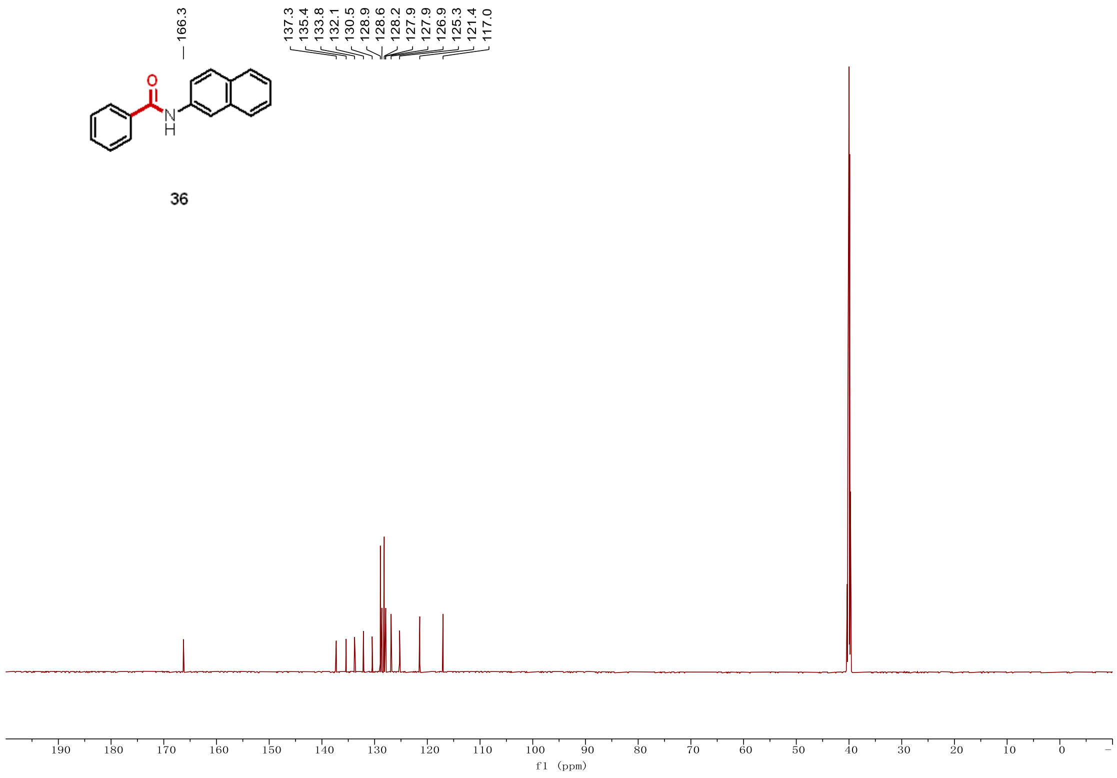

^{13}C NMR spectrum of **36** in DMSO-d_6 (176 MHz)
